# Mental health systems in Pacific island countries and territories: a scoping review of current evidence

**DOI:** 10.1016/j.lanwpc.2026.101856

**Published:** 2026-04-16

**Authors:** Jialing Lin, Xinrui Wei, Peixin Han, Tinghsuan Hsu, Jiaying Sun, Carey Marr, Rong Liu, Rohina Joshi, Minh Cuong Duong, Odille Chang, Xiwen Qin, Jinfeng Ding, Christine Y. Lu, Patricia M. Davidson, Michael Kidd

**Affiliations:** a International Centre for Future Health Systems, University of New South Wales, Sydney, New South Wales, Australia; b China General Practice of Department, Second Affiliated Hospital of Harbin Medical University, Harbin, Heilongjiang, China; c Faculty of Science and Technology, Beijing Normal-Hong Kong Baptist University, Zhuhai, Guangdong, China; d Global Business School for Health, University College London, London, United Kingdom; e University of California, San Diego, La Jolla, CA, USA; f Discipline of Psychiatry and Mental Health, School of Clinical Medicine, University of New South Wales, Sydney, New South Wales, Australia; g Centre for Infectious Diseases and Microbiology - Public Health, Westmead Hospital, Westmead, New South Wales, Australia; h Sydney Infectious Diseases Institute (Sydney ID), Faculty of Medicine and Health, The University of Sydney, Sydney, New South Wales, Australia; i School of Population Health, University of New South Wales, Sydney, New South Wales, Australia; j The George Institute for Global Health, UNSW, Sydney, Australia; k Fiji National University, Suva, Fiji; l Department of Public Health and Preventive Medicine, School of Medicine, Jinan University, Guangzhou, Guangdong, China; m School of Global and Population Health, The University of Western Australia, Perth, WA, Australia; n Xiangya School of Nursing, Central South University, Changsha, Hunan, China; o School of Pharmacy, Faculty of Medicine and Health, The University of Sydney, Sydney, New South Wales, 2006, Australia; p Kolling Institute, The University of Sydney Faculty of Medicine and Health and the Northern Sydney Local Health District, Sydney, New South Wales, Australia; q Nuffield Department of Primary Care Health Sciences, University of Oxford, Oxford, United Kingdom; r College of Health & Medicine, Australian National University, Canberra, Australia; s Department of Family and Community Medicine, University of Toronto, Canada

**Keywords:** Mental health systems, Pacific island countries and territories, Health system performance, Service delivery, Leadership, Workforce, Scoping review

## Abstract

Mental health systems are a critical component of health system performance in Pacific island countries and territories (PICTs), yet evidence remains uneven across settings. This scoping review mapped available evidence on mental health system performance in each PICT using the WHO Health System Building Blocks framework. We searched nine peer-reviewed databases and 13 grey literature sources (2015–2025). Of the 23,574 records identified, 91 studies were included, covering most PICTs. Most studies covered service delivery (n = 84), governance (n = 81), and workforce (n = 79). Findings revealed that systems remain predominantly hospital-centred with severe workforce shortages, limited financing, weak information systems, and fragile access to essential medicines. Although most PICTs have mental health policies, implementation capacity is constrained and fragmented. Some community-based and culturally embedded models exist, but are often poorly integrated into formal systems. Strengthening mental health systems in PICTs requires country-specific, system-wide reforms to address persistent inequities and enhance resilience against climate-related emergencies.

## Introduction

Mental health is essential to achieving health equity in the Pacific, shaping individual well-being, community cohesion, and economic resilience, as emphasised in the World Health Organization (WHO) Regional Framework for the Future of Mental Health in the Western Pacific 2023–2030.^1^ Mental health disorders have been one of the leading causes of morbidity and disability globally for decades but remain under-recognised and under-resourced in many Pacific Island countries and territories (PICTs).^2^ It is estimated that more than 1.7 million people were affected by mental disorders in 2023 in the Pacific, with approximately 252,000 living with disability.^2^ Self-harm, including suicide, also represents a significant burden, affecting over 16,000 people in the region, with 999 living with disability and 607 deaths recorded in 2023.^2^

Despite growing recognition of their importance, mental health systems, defined in line with WHO frameworks as the governance, financing, workforce, and service delivery arrangements that support mental health promotion, prevention, treatment, and recovery,^3^ face persistent challenges in the region, such as chronic underinvestment, limited workforce capacity, fragmented services, and weak integration into primary care and community-based models.^4^, ^5^, ^6^ These structural gaps are further compounded by geographical isolation and fragile infrastructure. Additionally, climate-related emergencies in the Pacific create a dual burden by simultaneously escalating psychological distress and physically damaging the already limited infrastructure required for service delivery.^5^^,^^7^^,^^8^

Evidence on mental health in PICTs remains fragmented and sparse, often derived from small-scale surveys or single-country studies.^4^^,^^6^^,^^8^, ^9^, ^10^ Previous research has largely focused on specific conditions, such as suicide,^9^ or on individual health system components, such as workforce, financing, service delivery, or the impacts of climate change, primarily within peer-reviewed journal articles, with limited integration of grey literature.^4^^,^^6^, ^7^, ^8^^,^^10^ As a result, few comprehensive regional syntheses of mental health system performance exist,^11^ constraining effective planning, monitoring, and resource allocation.

Global initiatives, such as the Sustainable Development Goals and the WHO's Mental Health Gap Action Program (mhGAP), demonstrate that universal health coverage and health equity cannot be realised without stronger, well-resourced, and equitable mental health systems.^12^, ^13^, ^14^, ^15^ These global commitments align with longstanding regional priorities, including the Pacific Healthy Islands Vision, which emphasises holistic wellbeing, equity, and resilient health systems.^16^ Yet in PICTs, scattered and incomplete evidence continues to hinder coordinated regional action and weakens alignment with these global commitments.^4^^,^^6^, ^7^, ^8^, ^9^, ^10^, ^11^ Without a comprehensive understanding of system capacity, PICTs risk falling further behind in achieving both national and international health goals.

This scoping review addresses these critical gaps by systematically mapping and synthesising current evidence on the status of mental health systems across 22 PICTs. By utilising the WHO Health System Building Blocks framework, the primary review question is: “*What is the current state of mental health system performance in each PICT?”* The secondary review question is: *“What gaps exist in the evidence, and what areas require further research or system strengthening to guide equity-focused regional strategies?”* By identifying progress and informing equity-focused regional strategies,^17^ this review seeks to provide a robust evidence base for future regional health strategies, ensuring that mental health system strengthening is grounded in the unique geographical, cultural, social, economic, and environmental realities of the Pacific.

## Methods

This scoping review adhered to the Preferred Reporting Items for Systematic Reviews and Meta-Analyses Extension for Scoping Reviews (PRISMA-ScR) statement to ensure rigorous and transparent reporting of the evidence base (see checklist from the Supplementary Table S1),^18^ and followed a study protocol (Supplementary File 1).

### Data sources

We searched both peer-reviewed publications and grey literature on mental health systems in PICTs, covering literature published from January 2015 to June 2025, aligning with the start of the Sustainable Development Goals era.^15^ A systematic search of peer-reviewed publications was conducted across eight databases: PubMed, CINAHL, EMBASE, PsycINFO, PAIS Index, Web of Science, the WHO Western Pacific Region Index Medicus, and Hyper Articles en Ligne.

To capture valuable policy documents, reports, and studies that may not be available in peer-reviewed journals, we also searched for grey literature from three key sources: Google search, grey literature databases, and targeted website sources. We used Google search to identify relevant grey literature using key words related to mental health systems and PICTs. For grey literature databases, we included Policy Commons, Dimensions, Organisation for Economic Co-operation and Development iLibrary, World Bank Documents and Reports, World Bank Open Knowledge Repository, and WHO Institutional Repository for Information Sharing. Targeted website sources included United Nations International Children's Emergency Fund (UNICEF) Pacific Islands, WHO Mental Health Atlas, Pacific Community, Pacific Island Health Officers Association, and Asian Development Bank.

### Search strategy and selection criteria

We defined mental health as a state of emotional, psychological, cognitive, and social well-being in which individuals can realise their abilities, manage the normal stresses of life, engage in productive work, and contribute to their community. This definition includes a broad spectrum of conditions, including common mental disorders (e.g., depression, anxiety), severe mental illnesses (e.g., schizophrenia, bipolar disorder), neurodevelopmental and behavioural disorders (e.g., autism spectrum disorder, attention-deficit/hyperactivity disorder, eating disorders), and neurocognitive disorders (e.g., dementia). Disorders primarily related to substance use were excluded from this review. The review focused on 22 PICTs, including American Samoa, the Cook Islands, Federated States of Micronesia (FSM), Fiji, French Polynesia, Guam, Kiribati, the Marshall Islands, Nauru, Niue, the Northern Mariana Islands, Palau, Samoa, the Solomon Islands, Tokelau, Tonga, Tuvalu, Vanuatu, the Pitcairn Islands, Wallis and Futuna, New Caledonia, and Papua New Guinea (PNG). Sociodemographic profiles of each PICT were shown in the Supplementary Table S2.

For the peer-review databases, a structured search strategy was developed using a combination of keywords and Medical Subject Headings related to mental health systems and PICTs, applied to titles and abstracts. Tailored search strategies were then developed for each grey literature database and source to optimise retrieval of relevant publications. To enhance the comprehensiveness of our review, we also manually screened the bibliographies of all included articles to identify additional relevant publications. Detailed search strategies are shown in the Supplementary Table S3.

Given the linguistic diversity of PICTs, the search focused on publications in English and French, as these are the official languages. Specifically, French is the official language in New Caledonia, French Polynesia, Vanuatu, and Wallis and Futuna. For searches conducted in these French-speaking PICTs, the English search strategies were appropriately adapted into French and reviewed by two senior experts in mental health, who are native French speakers.

The retrieved peer-reviewed publications were exported into Endnote to remove duplicates and then uploaded into Covidence (Veritas Health Innovation, Melbourne, Australia), a systematic review management software program for further screening. Grey literature sources were extracted separately and organised in a Microsoft Excel spreadsheet.

Retrieved articles were independently screened by two authors at the titles/abstract and full-text stages, with any disagreements resolved through discussion with the lead author (JL). To enhance consistency and methodological rigour, reviewer training sessions were conducted to ensure a shared understanding of the eligibility criteria, and calibration exercises were undertaken in which sample papers were reviewed collaboratively to align interpretations and strengthen decision-making. Both quantitative and qualitative research studies were eligible for inclusion if they reported on mental health systems in PICTs. Guided by the WHO Health System Building Blocks framework,^17^ we included studies addressing one or more of the six domains of mental health systems: leadership and governance, financing, health workforce, service delivery, access to essential medicines, and health information systems. Studies were excluded if they met any of the following criteria: (1) ineligible study types—conference abstracts, research protocols, and pre-prints; (2) publication outside the eligible period (i.e., not published between 2015 and 2025); (3) study settings outside the 22 PICTs; or (4) outcomes not related to the above six health system domains.

### Data extraction

A standardised data extraction form (Supplementary Table S4) was piloted on nine studies and refined through two iterative revisions. Data extraction was performed independently by two authors using a standardised data extraction sheet to capture study characteristics and thematic data, including author, year of publication, study location, study language, study design, study period, and study summary. In addition, data extraction was structured using a pre-defined framework in line with the six-domain WHO Health System Building Blocks as listed in the above section. For each building block, specific themes were charted. For example, under leadership and governance, data were charted across themes including policies and legislation; stakeholders; governance structures; and climate change or emergency-related governance considerations. Similar structured themes were applied to the remaining building blocks (Supplementary Table S4). Climate change and emergencies components were captured as cross-cutting themes across all building blocks. Any discrepancies were resolved through team discussion.

### Risk of bias assessment

Consistent with established guidance for scoping reviews, formal risk of bias or methodological quality assessment was not undertaken.^19^

### Data synthesis and analysis

Given the anticipated heterogeneity in policies and interventions and outcomes, data were synthesised using a narrative approach without meta-analysis, following the Synthesis Without Meta-analysis (SWiM) guidelines.^20^ Study characteristics were synthesised descriptively. The distribution of studies by country of data source and year of publication was visualised using bar plots.

Extracted data were synthesised using a framework-guided thematic approach. The six-domain WHO Health System Building Blocks and their predefined themes provided a deductive structure to guide data organisation. Within these domains and themes, patterns and new themes such as cultural considerations that emerged from the data were captured inductively through iterative review and comparison of study findings. This combined approach enabled a structured yet flexible cross-jurisdictional analysis of mental health system performance and resilience across PICTs.

Additionally, the development of mental health policy, legislative, and regulatory frameworks in PICTs was visualised over time to illustrate system evolution. Mental health workforce interventions were summarised descriptively.

### Ethics statement

This study is a scoping review of publicly available literature and does not involve the collection of primary data from human participants. As such, formal ethical approval was not required.

## Results

### Study selection

A total of 6764 records were identified from peer-reviewed databases. After duplicate removal, 5307 unique records remained, of which 4678 were excluded following title and abstract screening. Full texts of 629 articles were assessed for eligibility, and 50 studies met the inclusion criteria. An additional four studies were identified through bibliography screening, resulting in 54 eligible peer-reviewed studies.

From grey literature sources, 16,810 records were identified. After removing duplicates, 11,537 records remained, of which 11,062 were excluded based on title and abstract screening. The full texts of 475 records were assessed, and 36 studies met the inclusion criteria. One additional study was identified through bibliography screening, yielding 37 eligible grey literature studies.

**Fig. 1:**
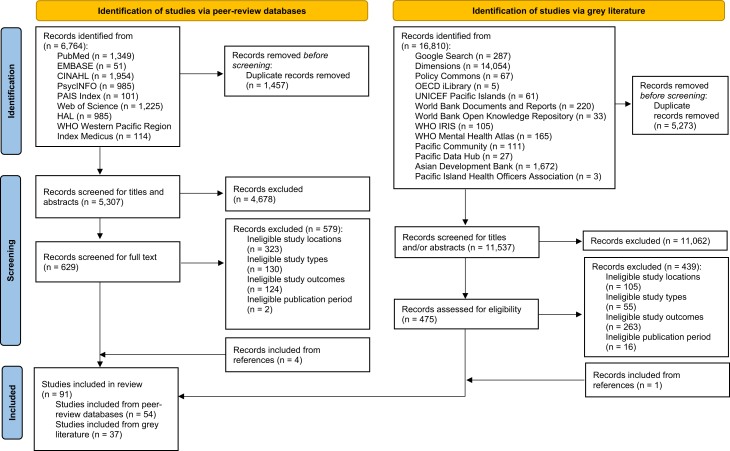
Flow diagram of study selection.

In total, 91 studies were included in the final review (Fig. 1).

### Study characteristics

Of the 91 included studies, most were published in English (n = 89), with two published in French. Forty were reports or position papers, 16 were qualitative studies, 11 were reviews or evidence syntheses, 10 were cross-sectional studies, five were mixed-methods studies, five were case studies, three were quasi-experimental studies, and one was a cohort study.

Most studies focused on Fiji (n = 27), followed by Vanuatu (n = 18), FSM (n = 15), PNG (n = 15), Samoa (n = 15), the Solomon Islands (n = 15), Kiribati (n = 13), the Marshall Islands (n = 12), Tonga (n = 12), the Cook Islands (n = 11), Tuvalu (n = 11), Palau (n = 10), and a few were on other PICTs.

**Fig. 2:**
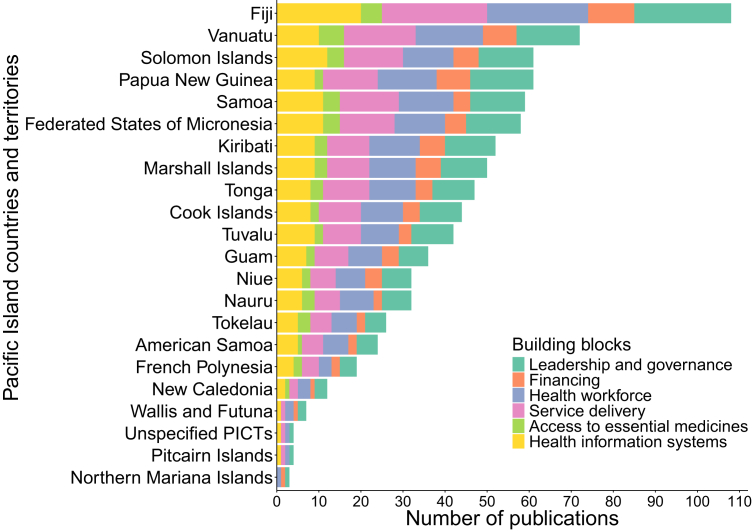
Distribution of publications by WHO Health System Building Blocks and Pacific Island countries and territories. Note: Studies may be included in more than one group.

The most frequently reported health system domain was service delivery (n = 84), followed by leadership and governance (n = 81), health workforce (n = 79), health information systems (n = 71), financing (n = 43), and access to essential medicines (n = 18). This distribution was broadly consistent across PICTs (Fig. 2).

A summary of study characteristics is presented in Table 1, with detailed information provided in the Supplementary Table S5.

**Table 1:** Summary of study characteristics.

Characteristics	Number of studies (% of total)
Total	91 (100.0)
Building blocks^a^	
Service delivery	84 (92.3)
Leadership and governance	81 (89.0)
Health workforce	79 (86.8)
Health information systems	71 (78.0)
Financing	43 (47.3)
Access to essential medicines	18 (19.8)
Study type	
Report or position paper	40 (44.0)
Qualitative study	16 (17.6)
Review and evidence synthesis	11 (12.1)
Cross-sectional study	10 (11.0)
Case study	5 (5.5)
Mixed-methods study	5 (5.5)
Quasi-experimental study	3 (3.3)
Cohort study	1 (1.1)
Study location^a^	
Fiji	27 (29.7)
Vanuatu	18 (19.8)
Federated States of Micronesia	15 (16.5)
Papua New Guinea	15 (16.5)
Samoa	15 (16.5)
Solomon Islands	15 (16.5)
Kiribati	13 (14.3)
Marshall Islands	12 (13.2)
Tonga	12 (13.2)
Cook Islands	11 (12.1)
Tuvalu	11 (12.1)
Palau	10 (11.0)
Guam	9 (9.9)
Nauru	8 (8.8)
Niue	8 (8.8)
American Samoa	6 (6.6)
Tokelau	6 (6.6)
French Polynesia	5 (5.5)
New Caledonia	3 (3.3)
Wallis & Futuna	2 (2.2)
Northern Mariana Islands	1 (1.1)
Pitcairn Islands	1 (1.1)
Unspecified PICTs	1 (1.1)
Language	
English	89 (97.8)
French	2 (2.2)

a Percentages exceed 100% because individual studies may address multiple building blocks or Pacific island countries and territories (PICTs).

### Service delivery

Service delivery for mental health was reported in 84 studies across 19 PICTs, with no data from Wallis & Futuna, New Caledonia, or the Northern Mariana Islands (Supplementary Table S6). Mental health service delivery across PICTs remained predominantly centralised and hospital-centred, with limited evidence of effective decentralisation to primary and community care. Fiji,^21^ PNG,^22^ Kiribati,^23^ the Solomon Islands,^24^ and Tonga^25^ relied heavily on a single national psychiatric hospital or small psychiatric units within general hospitals, typically located in capital cities. Community-based mental health facilities were sparse, particularly outside urban centres.

Geographic isolation substantially constrained access in archipelagic states such as FSM, Kiribati, Tuvalu, Tokelau, and Vanuatu, where outer islands frequently had no on-island mental health services.^26^, ^27^, ^28^ In addition to distance, access was further limited by factors such as scarce transport options, high travel costs, and the time required to leave work or manage household and family responsibilities, which together restricted service utilisation.^13^^,^^29^, ^30^, ^31^, ^32^ Several countries, including Fiji and PNG, reported that fewer than 10% of people requiring mental health care could access appropriate services.^32^^,^^33^

Innovative service delivery models have shown promise in some settings, including community recovery outreach and art-based programmes in Fiji,^34^ Skills for Life Adjustment and Resilience (SOLAR) in Tuvalu,^35^ church- and village-based psychosocial interventions in Samoa and Tonga,^36^ and post-disaster psychological first aid programmes in Vanuatu, Fiji, and the Solomon Islands.^37^ These approaches have enhanced access to culturally and contextually responsive strategies, yet their impact has been constrained by fragmentation, unevenly implementation, and weak integration into formal health systems.

### Leadership and governance

**Fig. 3:**
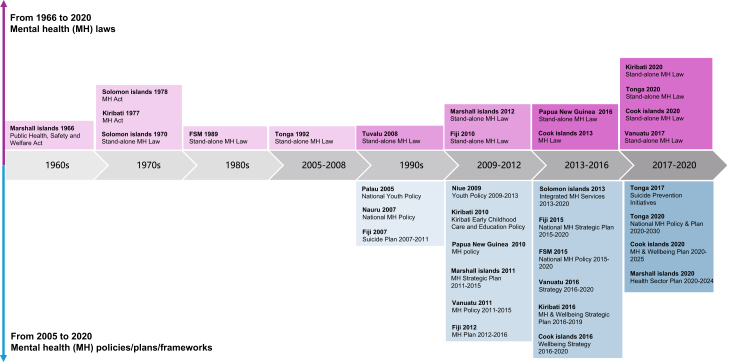
Timeline of the establishment of mental health policy, legislative, and regulatory frameworks in the Pacific Island countries and territories. FSM, Federated States of Micronesia.

Leadership and governance for mental health services were reported in 81 studies across 19 PICTs, with no data from Wallis & Futuna, New Caledonia, or the Northern Mariana Islands (Supplementary Table S6). Mental health governance across PICTs showed substantial variation in legal frameworks, policy maturity, and implementation capacity. Some legal frameworks established date back to the 1960s, while more recent ones have emerged post-2010 (e.g., several in 2020) (Fig. 3). Several PICTs, including the Solomon Islands (1978),^24^^,^^38^ FSM (1989),^29^ Fiji (2010),^21^^,^^32^ the Marshall Islands (2012),^39^ the Cook Islands (2013),^40^ Tuvalu (2017),^41^ Vanuatu (2017),^28^ PNG (2018),^22^ Kiribati (2020),^42^ and Tonga (2020),^43^ reported the existence of stand-alone mental health legislation, although the scope and alignment with contemporary human rights standards differed widely. Fiji's Mental Health Decree (2010) is a notable example of a comprehensive, rights-based law aligned with international standards.^21^

PICTs such as the Nauru (2007),^44^ PNG (2010),^22^ Marshall Islands (2011),^45^ Vanuatu (policy 2011),^28^ Fiji (2015),^21^ FSM (2015),^46^ Kiribati (2016),^23^ Vanuatu (strategy 2016),^28^ the Cook Islands (2020),^40^ the Marshall Islands (2020),^45^ and Tonga (2020)^25^ have developed national mental health policies or strategic plans. Despite the expansion of national strategies, a critical implementation gap remains, as many frameworks lack the operational indicators and secured budgetary allocations necessary for enactment. In Palau and the Solomon Islands, national mental health policies were reported as draft or not formally endorsed, limiting governance authority.^24^^,^^47^ Some specific mental health frameworks were also found such as suicide prevention^48^^,^^49^ and integration of traditional healing.^44^^,^^48^ Some broader health frameworks such as youth policies and wellbeing strategies also involved mental health related content.^30^^,^^50^^,^^51^

Governance structures were predominantly centralised within Ministries of Health, including in Fiji,^21^ Samoa,^52^ Kiribati,^53^ the Solomon Islands,^38^ and Vanuatu.^28^ Traditional leadership systems, extended family networks, and faith-based institutions played a central governance and decision-making role in FSM,^46^ Tonga,^31^^,^^54^ Samoa,^37^ Tokelau,^27^ and Tuvalu,^55^ shaping culturally embedded mental health responses. Independent oversight bodies for mental health legislation were reported but often non-functional or limited in authority, particularly in Kiribati,^42^ Tuvalu,^41^ and Palau.^47^

### Health workforce

A total of 79 studies reported on the health workforce in 20 PICTs, with no data from New Caledonia or the Northern Mariana Islands (Supplementary Table S6). Workforce shortages are universal across the region, characterised not only by low clinical density but also by a severe maldistribution that favours urban centres over remote island populations. PNG reported fewer than ten psychiatrists nationally for a population exceeding ten million,^22^ while Tokelau,^27^ Nauru,^56^ Tuvalu,^41^ and Niue^57^ had no resident psychiatrists, relying on visiting specialists or overseas referrals. Workforce density was lowest in PNG,^22^ the Solomon Islands,^24^ and FSM,^46^ each reporting fewer than three mental health professionals per 100,000 population. In contrast, Palau and Niue reported comparatively higher mental health workforce densities, largely driven by nursing cadres rather than specialist clinicians.^47^^,^^57^ Child and adolescent mental health specialists were largely absent across the region, with limited capacity reported in Fiji,^21^ Tonga,^25^ and Vanuatu.^28^

Interventions were predominantly oriented towards capacity building, task-sharing, and system-level strengthening (Table 2 and Supplementary Table S7). Although the WHO mhGAP training is the dominant workforce intervention and has demonstrated effectiveness,^14^ evidence suggests it remains episodic and lacks integration into formal career pathways or sustained clinical supervision models in several countries such as Fiji, Samoa, Tonga, Vanuatu, Kiribati, Tuvalu, Palau, FSM, and PNG.^12^, ^13^, ^14^^,^^53^ The WHO proMIND initiative has enhanced the effect of mhGAP by supporting workforce and system development through mental health profiling, situational analysis, and policy and service planning in the Solomon Islands, Tonga, Vanuatu, Fiji, and PNG.^58^^,^^59^

**Table 2:** Description of mental health workforce interventions in the Pacific Island countries and territories (PICTs).

Intervention	Organisation	Content	PICTs
mhGAP training	WHO; Ministry of Health	mhGAP-based training incorporating discussions, case studies, and role-play to build capacity among doctors and nurses in the assessment and management of priority mental health conditions.	Fiji, Kiribati, Tonga, Vanuatu, Marshall Islands, Federated States of Micronesia, Palau, Northern Mariana Islands, Solomon Islands, Papua New Guinea, Cook Islands, Samoa, Tuvalu, Nauru
proMIND training	WHO	Development of mental health profiles and situational analyses; support for policy development and mental health system assessment and planning.	Solomon Islands, Tokelau, Tonga, Vanuatu, Fiji, Papua New Guinea, Cook Islands, Samoa, Kiribati, Marshall Islands, Federated States of Micronesia, Palau, Tuvalu, Nauru, Niue, Wallis and Futuna
UNICEF-supported capacity building	UNICEF; WHO; UNFPA; UN Women; IFRC; Pacific Community	Psychosocial support and psychological first aid for children; mental health and psychosocial support (MHPSS); child protection; capacity strengthening for frontline and community-based workers.	Cook Islands, Fiji, Kiribati, Marshall Islands, Federated States of Micronesia, Nauru, Niue, Palau, Samoa, Solomon Islands, Tokelau, Tonga, Tuvalu, and Vanuatu
RANZCP capacity-building	RANZCP	Online Pacific Health Exchange (five-year program); Pasifika Study Group workshops; training in child and adolescent mental health, trauma-informed care, developmental disorders, and youth substance use.	Vanuatu, Kiribati, Samoa, Papua New Guinea, Fiji
Postgraduate diploma in mental health	Fiji National University	One-year full-time postgraduate programme providing clinical psychiatric training and culturally relevant mental health practice; graduates serve as frontline mental health practitioners.	Fiji, Kiribati, Tonga, Vanuatu, Cook Islands, Federated States of Micronesia
Master of Medicine in Psychiatry	University of Papua New Guinea	A postgraduate clinical medical specialist training program designed to train medical doctors to become psychiatrists — specialists in the diagnosis, treatment, prevention, and management of mental health disorders.	Papua New Guinea, Fiji
Leadership in mental health training	James Cook University	Leadership and policy development training focused on mental health service planning and regional collaboration; delivered in Cairns, Australia.	Delegates from multiple Pacific Island countries
St Giles Hospital in-service training and arts-based programmes	St. Giles Psychiatric Hospital; Fiji Ministry of Health	In-service training, mental health awareness activities, and community mental health programmes; provision of specialised services and training support to neighbouring Pacific Island countries.	Fiji; regional Pacific Island countries
Solomon Islands National University mental health training programmes	Solomon Islands National University, School of Nursing and Allied Health Sciences	Pre-service nursing and allied health education incorporating mental health components; established in 2013 within the national university system.	Solomon Islands
OPHELIA training programme	St. Vincent's Mental Health (Australia); Fiji National University; RANZCP Faculty of Child and Adolescent Psychiatry	Online specialised child and adolescent mental health training delivered via telehealth; development of a Pacific-based community of practice; launched June 2020.	Pacific-based health workers from multiple countries
SOLAR programme	Tuvalu Association of Non-Government Organisations; Congregational Christian Church of Tuvalu (Te Ekalesia Kelisiano Tuvalu)	Brief, scalable psychosocial skills-based intervention designed to reduce distress and adjustment difficulties following disasters.	Tuvalu

IFRC, International Federation of Red Cross and Red Crescent Societies; mhGAP, Initiated mental health Gap Action Programme; MHPSS, mental health and psychosocial support; OPHELIA, Optimising Health Literacy and Access; RANZCP, Royal Australian and New Zealand College of Psychiatrists; SOLAR, Skills for Life Adjustment and Resilience; UNFPA, United Nations Population Fund; UNICEF, United Nations Children's Fund programme; UN Women, United Nations Entity for Gender Equality and the Empowerment of Women; WHO, World Health Organization.

Additional workforce strengthening initiatives targeted frontline, community-based, and specialist-adjacent cadres. UNICEF-supported programmes focused on mental health and psychosocial support, psychological first aid, child protection, and disaster response, with capacity-building activities reported across Fiji, Kiribati, Samoa, Tonga, and Vanuatu.^60^ Regionally led initiatives included multi-year training programmes delivered by the Royal Australian and New Zealand College of Psychiatrists, such as the Pacific Health Exchange and Pasifika Study Group workshops, which provided education in child and adolescent mental health, trauma-informed care, developmental disorders, and youth substance use in Fiji, PNG, Samoa, Kiribati, and Vanuatu.^61^ Formal education pathways were reported in some settings, including the postgraduate diploma in mental health at Fiji National University,^62^ which trained frontline practitioners serving Fiji and neighbouring PICTs, and the integration of mental health content into pre-service nursing and allied health curricula at Solomon Islands National University.^38^

Innovative delivery models were also reported within the workforce domain. Telehealth-enabled initiatives, such as the Optimising Health Literacy and Access (OPHELIA) programme, delivered specialised child and adolescent mental health training and supported the development of a Pacific-wide community of practice.^63^ In Fiji, St Giles Psychiatric Hospital contributed to regional workforce development through in-service training, mental health awareness activities, and arts-based community programmes, while also providing training support to neighbouring PICTs.^62^ At the community level, brief, scalable psychosocial interventions such as the SOLAR programme in Tuvalu focused on strengthening local workforce capacity in post-disaster contexts.^35^ Task-sharing extended beyond the formal health sector, with traditional healers, clergy, and faith-based workers reported as part of mental health care pathways in Tonga, Samoa, Guam, the Marshall Islands, and FSM, reflecting locally embedded workforce models.^44^^,^^54^^,^^64^^,^^65^

### Health information systems

Seventy-one studies reported on the health information systems in 16 PICTs, with no data from American Samoa, Wallis & Futuna, Guam, New Caledonia, Niue, or the Northern Mariana Islands (Supplementary Table S6). Mental health information systems in most PICTs were characterised by data fragmentation, lacking the robust surveillance required to track prevalence trends, service utilisation, or longitudinal patient outcomes. Major data gaps affected rural and outer-island populations in Fiji,^66^ PNG,^33^ the Solomon Islands,^38^ Kiribati,^26^ and Vanuatu,^67^ where mental health data were rarely captured in national health bulletins.

Child and adolescent mental health data were especially limited. The Global School-based Student Health Survey represented one of the few consistent data sources in Paula,^30^ Tokelau,^27^ and Vanuatu,^68^ while several PICTs reported no adolescent mental health data. Suicide and self-harm surveillance systems were improving in Fiji, Samoa, the Cook Islands, and the Solomon Islands, largely through WHO-supported initiatives, although regional coverage remained uneven.^1^^,^^9^^,^^69^^,^^70^

### Health financing

Forty-three studies reported on financing in 18 PICTs, with no data from Wallis & Futuna, New Caledonia, the Northern Mariana Islands, or the Pitcairn Islands, suggesting a lack of fiscal transparency in these jurisdictions (Supplementary Table S6). Mental health financing across PICTs was consistently characterised by low levels of public investment and a systemic reliance on volatile external development assistance. Government mental health expenditure ranged from approximately 0.4% of total health spending in the Marshall Islands^39^ to around 1.0–1.5% in Fiji,^21^ Tonga,^25^ PNG,^22^ and Vanuatu.^28^ Kiribati reported a higher proportional allocation (5%), although all expenditure was directed to mental hospitals.^71^

Most PICTs operated tax-based public health systems, with government-funded mental health services and psychotropic medicines provided free at the point of care, including in Fiji,^21^ Samoa,^52^ Tonga,^25^ the Solomon Islands,^38^ Tuvalu,^41^ Kiribati, and Vanuatu.^28^ However, the absence of ring-fenced mental health budgets was common, particularly in Samoa,^52^ Nauru,^56^ FSM,^29^ and Kiribati,^71^ creating a budgetary invisibility that prevents the scaling of community-based services.

Across multiple PICTs, including Fiji,^21^ PNG,^22^ Kiribati,^71^ and Niue,^57^ more than 90% of mental health expenditure was allocated to hospital-based services, leaving limited funding for community-based care, prevention, or psychosocial interventions. Several countries, notably FSM,^29^ the Marshall Islands,^59^ and Nauru,^56^ relied heavily on external development assistance (e.g., the WHO, Australia's Department of Foreign Affairs and Trade, New Zealand Aid, the Asian Development Bank, and the United States), particularly for workforce training and post-disaster responses.

### Access to essential medicines

Access to essential medicines was the least-documented building block, identified in 18 studies from 17 PICTs (19.8% of the total evidence base), highlighting a critical gap in pharmaceutical supply chain transparency. There were no data from American Samoa, French Polynesia, Wallis & Futuna, New Caledonia, or the Northern Mariana Islands (Supplementary Table S6). Most PICTs reported inclusion of essential psychotropic medicines within national formularies, consistent with mhGAP guidance,^14^ including Fiji,^21^ Samoa,^1^ Tonga,^25^ the Solomon Islands,^38^ Tuvalu,^41^ Kiribati,^71^ and Vanuatu.^28^ However, fragmented supply chains frequently lead to treatment discontinuity, with outer island populations facing the highest risk of chronic medication stock-outs.

In jurisdictions such as PNG,^72^ Vanuatu,^67^ the Marshall Islands,^59^ and FSM,^73^ the therapeutic range is constrained by a reliance on older-generation psychotropics and frequent disruptions in the procurement of essential agents. Although medicines were generally provided free at the point of care, interruptions in supply often resulted in treatment discontinuity or private out-of-pocket purchasing.^67^^,^^72^

### Comparative overview of mental health systems across PICTs

Overall, mental health systems across PICTs show substantial variability. Countries like Fiji, Tonga, and Cook Islands have more developed policies, community-based services, and workforce training, while others, such as Nauru, Pitcairn Islands, and Tokelau, face limited workforce, infrastructure gaps, and fragmented services. Common strengths include mhGAP implementation, cultural adaptation, and post-disaster interventions, whereas challenges span workforce shortages, limited medicine access, weak data systems, and funding constraints. The table summarises overall system performance qualitatively, highlighting key strengths, gaps, and cross-cutting issues to inform regional priorities (Supplementary Table S8).

## Discussion

### Principal findings

This scoping review synthesised evidence from 91 peer-reviewed and grey literature sources to map mental health system performance across 22 PICTs using the WHO Health System Building Blocks framework. The findings reveal pervasive and interrelated challenges across all system domains, including service delivery, governance, workforce, financing, information systems, and access to essential medicines. Despite increasing policy attention to mental health and the presence of innovative local initiatives, mental health systems in PICTs remain predominantly hospital-centred, under-resourced, and inequitably distributed. Limited adaptation to epidemiological transition has left health systems ill-equipped to meet growing population and mental health needs, particularly for people living in outer islands, children and adolescents, and communities affected by climate-related emergencies. These findings highlight persistent structural constraints that limit equitable access to mental health care across the region.

### Persistent hospital-centred service delivery and geographic inequities

Across most PICTs, mental health service delivery remains concentrated in national psychiatric hospitals or small psychiatric units within general hospitals, typically located in capital cities. This centralisation, reported in Fiji, PNG, Kiribati, the Solomon Islands, and Tonga, continues to constrain access for populations in rural and outer island settings.^21^, ^22^, ^23^, ^24^, ^25^ These geographic barriers are compounded by climate-related hazards (e.g., cyclones, flooding) and culturally embedded help-seeking practices, which further limit timely access to care, particularly for youth, women, and residents of remote islands.^33^^,^^50^^,^^74^ Geographic dispersion and transport barriers were particularly pronounced in archipelagic states such as FSM, Kiribati, Tuvalu, Tokelau, and Vanuatu, where on-island mental health services were often absent.^26^, ^27^, ^28^ The finding that fewer than 10% of people requiring care are able to access appropriate services in Fiji and PNG underscores the magnitude of the treatment gap in the region.^32^^,^^33^

At the same time, this review identified promising community-based, culturally responsive, and contextually embedded service delivery models, including recovery outreach and art-based programmes in Fiji, church- and village-based psychosocial interventions in Samoa and Tonga, post-disaster SOLAR programmes in Tuvalu, and psychological first aid initiatives in Vanuatu and the Solomon Islands.^34^, ^35^, ^36^, ^37^ While these models appear more acceptable and contextually appropriate, they remain fragmented, unevenly implemented, and weakly integrated into formal health systems. Their limited scale-up reflects broader system constraints, including inadequate financing, workforce shortages, and insufficient governance and coordination mechanisms.^75^

### Governance advances without commensurate implementation capacity

Mental health governance frameworks across PICTs have expanded over the past two decades, with several countries enacting stand-alone mental health legislation or developing national policies and strategies. Fiji's Mental Health Decree (2010) represents a notable example of a comprehensive, rights-based framework aligned with international standards.^21^ However, in many PICTs, mental health legislation and policies lack operational detail, implementation plans, defined indicators, or secured funding. Draft or non-endorsed policies, reported in Palau and the Solomon Islands, further weaken governance authority which likely due to lack of implementation strategies.^24^^,^^47^

Governance structures were largely centralised within Ministries of Health, with limited formalised cross-sectoral coordination. Yet, traditional leadership systems, extended family networks, and faith-based institutions played a central role in shaping mental health responses in FSM, Tonga, Samoa, Tokelau, and Tuvalu.^27^^,^^31^^,^^37^^,^^46^^,^^54^^,^^55^ While these structures offer culturally grounded pathways to care, their integration into formal governance arrangements is inconsistent. Weak or non-functional oversight bodies, particularly in Kiribati, Tuvalu, and Palau, further constrained accountability and rights protection.^41^^,^^42^^,^^47^ Overall, these findings highlight a persistent implementation gap between policy intent and system capacity, reflecting a broader challenge faced by small island health systems with limited administrative, technical, and fiscal space.

### Severe workforce shortages and fragile capacity-building models

Health workforce constraints have emerged as one of the most critical system bottlenecks across all PICTs. Severe shortages of specialised mental health professionals were universal, with pronounced disparities between urban and rural areas, and between capitals and outer islands. Several PICTs report fewer than three mental health professionals per 100,000 population,^22^^,^^24^^,^^46^ and many, such as Tokelau, Nauru, Tuvalu, and Niue, had no resident psychiatrists.^27^^,^^41^^,^^56^^,^^57^ Expertise in child and adolescent mental health was largely absent across the region, despite growing youth mental health needs.^21^^,^^25^^,^^28^

Workforce interventions have predominantly focused on capacity building and task-sharing, most commonly through WHO mhGAP training.^14^ While mhGAP was widely reported, its implementation remained inconsistent and weakly embedded within national health systems. Training initiatives were frequently donor-supported, episodic, and insufficiently linked to supervision, career pathways, or retention strategies.^12^, ^13^, ^14^^,^^53^ Complementary initiatives, including WHO proMIND,^58^^,^^59^ UNICEF-supported psychosocial programmes,^60^ regional psychiatric training collaborations, and postgraduate education pathways in Fiji and the Solomon Islands,^38^^,^^62^ demonstrate the potential of regional and locally anchored workforce development. However, the absence of systematic evaluation of workforce outcomes, beyond training outputs, limits understanding of their effectiveness and sustainability.

Notably, this review highlights the importance of non-traditional and community-based workforce models, including the involvement of traditional healers, clergy, and faith-based workers in mental health care pathways.^44^^,^^54^^,^^64^^,^^65^ In several PICTs, reliance on traditional healers and faith-based actors reflects both clinical workforce scarcity and culturally embedded parallel system that remains largely unrecognised by formal governance structures. While these models provide important culturally grounded responses to workforce gaps, they also raise critical questions regarding quality assurance, supervision, and integration within formal health systems.

### Chronic underfinancing and reliance on external assistance

Mental health financing across PICTs was consistently characterised by low levels of public investment, with most countries allocating less than 2% of total health expenditure to mental health,^21^^,^^22^^,^^25^^,^^28^^,^^39^ below the global median of 2.1%.^76^ Even in countries with relatively higher proportional allocations, such as Kiribati, funding was largely absorbed by mental hospitals, leaving little investment for community-based services, prevention, or psychosocial care.^71^ The absence of ring-fenced mental health budgets in several PICTs further constrained sustainability and service expansion.

Although most PICTs operate tax-funded health systems, providing mental health services and psychotropic medicines free at the point of care, reliance on external development assistance persists, particularly for workforce training and post-disaster responses.^10^^,^^29^^,^^56^^,^^59^ This donor dependence system contributes to persistent fragmentation, as episodic donor-funded projects frequently bypass national health systems, limiting the development of long-term, sustainable infrastructure. The predominance of hospital-based expenditure mirrors global patterns but is especially problematic in geographically dispersed island contexts where community-based care is essential for equitable access.

### Weak health information systems and limited data for planning

Mental health information systems across PICTs were consistently weak or fragmented. Major gaps in routine data on prevalence, service utilisation, and outcomes, particularly for rural populations and outer islands. Child and adolescent mental health data were especially scarce, with the Global School-based Student Health Survey representing one of the few consistent data sources.^27^^,^^30^^,^^68^ While suicide and self-harm surveillance has improved in some PICTs through WHO-supported initiatives, coverage remains uneven.^1^^,^^9^^,^^69^^,^^70^

These data limitations constrain service planning and resource allocation, and explain the predominance of descriptive and cross-sectional studies in literature. The lack of longitudinal, implementation, and outcome-focused research reflects broader system weaknesses and underscores the need for investment in mental health surveillance and locally led research capacity.

### Access to essential medicines remains fragile

Although most PICTs include essential psychotropic medicines in national formularies and provide them free at the point of care, supply chain disruptions, stock-outs, and limited medication ranges were common, particularly in outer islands.^59^^,^^67^^,^^72^^,^^73^ Reliance on older psychotropic agents and interruptions in supply risk discontinuity and undermine quality of care.^59^^,^^67^^,^^72^^,^^73^ These challenges further highlight the vulnerability of small island supply chains and the need for stronger procurement and distribution systems.

### Implications for policy and practice

By providing PICT-specific evidence across the WHO Health System Building Blocks, this review offers a strong foundation for context-sensitive mental health system reform in the Pacific. The findings indicate that fragmented, hospital-centred, and donor-driven models remain inadequate, underscoring the need to shift toward integrated, community-oriented, and resilient systems of care through international collaboration.^77^ These conclusions align with recent calls for action from Pacific leaders and WHO to address widening mental health needs, through concrete system-level reforms and coordinated investments.^78^

To translate these findings into actionable policy, PICT governments and regional partners should focus on embedding mental health within primary health care and universal health coverage reforms by establishing routine screening, clear referral pathways, and supervision structures across formal and informal care networks. They should strengthen sustainable financing by introducing ring-fenced budgets for mental health, including resources for workforce training, essential medicines, and community-based programmes. Workforce capacity should be expanded through structured mhGAP training, formal career pathways, ongoing supervision, and regional mentorship programmes, including engagement with traditional healers, clergy, and faith-based community workers where culturally appropriate.

Importantly, policy and planning should explicitly recognise the role of informal care networks and culturally embedded actors, including traditional leadership systems, extended family networks, and faith-based institutions, in mental health support. These actors often provide first-line care, guidance, and psychosocial support, particularly in rural and outer-island communities where formal services are limited. Integrating these informal systems into formal health system planning and community-based models can enhance accessibility, acceptability, and cultural appropriateness of mental health services.

Culturally grounded, community-based models, should be scaled and integrated into health system planning, aligned with the Healthy Islands Vision 2050 and WHO Western Pacific Region mental health priorities.^1^^,^^16^ Interventions should also reflect local explanatory models of mental health and illness, stigma-reduction strategies, and culturally informed help-seeking practices, ensuring that services resonate with community values and expectations.

Given the disproportionate exposure of PICTs to climate change and disasters, policy and planning should integrate mental health into disaster preparedness and climate resilience frameworks, recognising climate change as an immediate system stressor rather than a future risk. Resilient systems must include decentralised supply chains and disaster-ready community networks capable of functioning when centralised hospital services are disrupted.

The review also highlights uneven evidence across PICTs. Several settings, including French Polynesia, New Caledonia, Wallis & Futuna, the Northern Mariana Islands, and the Pitcairn Islands, remain under-represented in the literature, limiting visibility of system performance and population needs. Targeted investment in routine mental health data systems should capture both formal and informal care pathways, supported by locally led research and regional academic partnerships, to ensure that regional mental health reforms are inclusive, culturally relevant, and equitable rather than concentrated in a few better-documented countries.

### Strengths and limitations

This review draws on a large and diverse body of peer-reviewed and grey literature and applies a structured, system-level analytical framework. A key strength is the country-specific mapping of evidence for each PICT, enabling more tailored and contextually relevant insights for policy and practice. The use of the WHO Health System Building Blocks framework also facilitates comparability across settings and system domains.

However, several limitations should be noted. The evidence base was unevenly distributed across PICTs, with substantial variability in both the volume and depth of available studies. Many included studies were descriptive in nature, limiting the ability to draw conclusions about intervention effectiveness or causal relationships. As a scoping review, no formal quality appraisal was undertaken, and publication bias towards donor-supported initiatives is likely. Additionally, although French-language databases were searched, the small number of French-language sources identified (n = 2) suggests a potential language bias in the indexed literature.

Finally, our focus on studies published in the last decade means that earlier system-strengthening initiatives, often led by local champions with support from regional or international organisations,^79^ are not fully captured, despite providing valuable lessons for policy and practice. Broader determinants of mental health, such as urbanisation, migration, and gender-based violence, were also not consistently addressed in the included literature and thus represent important contextual factors to consider when interpreting our findings.

## Conclusions

Despite the expansion of national mental health policies across the Pacific, a profound implementation gap persists. Mental health systems remain largely centralised and hospital-centred, leaving them structurally ill-equipped to respond to the unique geographical dispersion and cultural diversity of PICT populations. By synthesising evidence for each PICT, this review provides a foundation for context-appropriate, system-wide reforms. Strengthening mental health systems in the Pacific requires a decisive shift from episodic, donor-dependent initiatives toward resilient system architectures that prioritises ring-fenced financing, decentralised and integrated service delivery, and the formal integration of culturally embedded community models. These changes are essential to advancing long-term equity and ensuring mental health system resilience in a region increasingly vulnerable to climate-related emergencies.

## Contributors

JL contributed to conceptualisation, project administration, formal analysis, and writing—original draft. JL, XW, PH, TH, JS, CM, and RL contributed to literature search and screening. JL, XW, PH, TH, and JS contributed to data curation. JL and XW contributed to data analysis and visualisation. MK contributed to supervision. JL, XW, PH, TH, JS, CM, RL, RJ, MCD, OC, XQ, JD, CYL, PMD, and MK contributed to methodology and writing—review & editing. JL, XW, PH, TH, JS, CM, RL, RJ, MCD, OC, XQ, JD, CYL, PMD, and MK approved the final version of the manuscript.

## Data sharing statement

Data sharing is not applicable to this article as no datasets were generated during the current study.

## Declaration of interests

The authors declare no competing interests.

## Appendix A Supplementary data

Supplementary Materials

Study Protocol
