# Supplementary material for: Mental health systems in Pacific island countries and territories: a scoping review of current evidence
Source: Lancet Reg Health West Pac. 2026 Apr 16;69:101856. doi: 10.1016/j.lanwpc.2026.101856 (PMC13101603; doi:10.1016/j.lanwpc.2026.101856)
Supplement: Supplementary Materials [file mmc1.docx]

**Mental health systems in Pacific island countries and territories: a scoping review of current evidence**

**Supplementary material**

[**Supplementary File 1** Strengthening mental health systems in Pacific island countries and territories: A scoping review protocol 1](#_Toc222775626)

[INTRODUCTION 1](#_Toc222775627)

[METHODS 2](#_Toc222775628)

[Search strategy 2](#_Toc222775629)

[Peer-review databases 2](#_Toc222775630)

[Grey literature 4](#_Toc222775631)

[Study selection 7](#_Toc222775632)

[Data synthesis and analysis 7](#_Toc222775633)

[References 8](#_Toc222775634)

[**Supplementary Table 1** Preferred Reporting Items for Systematic reviews and Meta-Analyses extension for Scoping Reviews (PRISMA-ScR) Checklist 9](#_Toc222775635)

[**Supplementary Table 2** Country profile 12](#_Toc222775636)

[**Supplementary Table 3** Search strategy 14](#_Toc222775637)

[**Supplementary Table 4** Data extraction form 24](#_Toc222775638)

[**Supplementary Table 5** Included studies 25](#_Toc222775639)

[**Supplementary Table 6** Mental health system performance across the six building blocks by Pacific Island countries and territories (PICTs) 32](#_Toc222775640)

[**Supplementary Table 7** Mental health workforce interventions by Pacific Island countries and territories (PICTs) 49](#_Toc222775641)

[**Supplementary Table 8** Summary of mental health system performance across Pacific Island countries and territories (PICTs) 50](#_Toc222775642)

**Supplementary File 1** Strengthening mental health systems in Pacific island countries and territories: A scoping review protocol

# INTRODUCTION

Mental health is a critical component of overall well-being, yet it remains a neglected area in many Pacific Island Countries and Territories (PICTs) [1]. The region is characterised by diverse cultures, languages, and socioeconomic contexts, which contribute to complex and varied mental health needs [2, 3]. Climate change further intensifies these challenges, as rising sea levels, extreme weather events, and other environmental disruptions displace communities, disrupt livelihoods, and threaten cultural continuity [4]. Such stressors increase psychological distress and place additional pressure on already limited mental health systems. While multiple, interconnected barriers to care exist, comprehensive information on system capacity, resources, and service delivery remains scarce.

Although efforts have been made to strengthen mental health systems across PICTs, progress has been uneven, and many countries remain underprepared to respond to emerging challenges, particularly during crises or emergencies. Limited data and research further constrain the development of effective policies and context-specific programmes, and formal health systems often operate alongside traditional leadership structures, extended family networks, and faith-based institutions, which play central roles in shaping community responses to mental health needs.

This scoping review synthesises available evidence on mental health systems across 22 PICTs, including American Samoa, Cook Islands, Federated States of Micronesia, Fiji, French Polynesia, Guam, Kiribati, Marshall Islands, Nauru, Niue, Northern Mariana Islands, Palau, Samoa, Solomon Islands, Tokelau, Tonga, Tuvalu, Vanuatu, Pitcairn Islands, Wallis and Futuna, New Caledonia, and Papua New Guinea. The review applies the World Health Organization (WHO) Health Systems Building Blocks framework to examine:

1. Leadership and governance – policies, strategic planning, regulation, and coordination of mental health services.
2. Service delivery – availability, quality, and accessibility of mental health care.
3. Health workforce – distribution, training, and competency of mental health personnel.
4. Financing – funding arrangements, resource allocation, and sustainability of services.
5. Medines – access to essential medications and therapeutic interventions.
6. Health information systems – availability and use of data to guide planning, monitoring, and evaluation.

The influence of climate change and emergencies will be considered across all building blocks to highlight their pervasive effect on mental health systems.

# METHODS

This review will be conducted adhere to the Preferred Reporting Items for Meta-Analysis and Systematic Reviews Extension for Scoping Reviews (PRISMA-ScR) statement [5].

##

## Search strategy

The review will identify both peer-reviewed and grey literature addressing the mental health systems in PICTs. Given the linguistic diversity of the region, the search will focus on English- and French-language publications. French is an official language in New Caledonia, French Polynesia, Vanuatu, and Wallis and Futuna; for these countries, English search terms will be adapted into French to capture relevant literature.

### Peer-review databases

A systematic search will be conducted across multiple bibliographic databases, including PubMed, CINAHL, EMBASE, Web of Science, PsycINFO, PAIS Index, and the WHO Western Pacific Region Index Medicus (WPRO). These databases were selected to cover a wide spectrum of mental health and health system literature relevant to PICTs. The search will include studies published from 2015 to the present, reflecting the period following the launch of the Sustainable Development Goals (SDGs) and capturing contemporary policies, challenges, and innovations in mental health care within the region.

A structured search strategy combining keywords and Medical Subject Headings (MeSH terms) will be applied to titles and abstracts (see Table 1). To ensure comprehensiveness, reference lists of included articles will also be hand-searched for additional relevant studies. Searches will be conducted in both English and French, with French-language searches applied to databases covering French-speaking PICTs. Additional French-language databases may be consulted in collaboration with French-speaking researchers to maximise coverage. All retrieved records will be managed using Covidence software for screening.

**Table 1 Search string for peer-review databases**

| **Search** | **Query** |
| --- | --- |
| #1 | “mental health”[MeSH Terms] OR “mental disorders”[MeSH Terms] OR “psychological well-being”[MeSH Terms] OR “anxiety”[MeSH Terms] OR “depression”[MeSH Terms] OR “euphoria”[MeSH Terms] OR “stress, psychological”[MeSH Terms] OR “self-Injurious behavior”[MeSH Terms] OR “epilepsy”[MeSH Terms] OR mental disorder*[tiab] OR mental disease*[tiab] OR mental illness*[tiab] OR mental health[tiab] OR mental well*being[tiab] OR psychiatric disorder*[tiab] OR psychiatric disease*[tiab] OR psychiatric illness*[tiab] OR psychiatric health[tiab] OR psychiatric well-being[tiab] OR psychosis[tiab] OR psychological disorder*[tiab] OR psychological disease*[tiab] OR psychological illness*[tiab] OR psychological health[tiab] OR psychological well*being[tiab] OR psychotic disorder*[tiab] OR psychotic disease*[tiab] OR psychotic illness*[tiab] OR psychotic health[tiab] OR psychotic well*being[tiab] OR developmental disorder*[tiab] OR developmental disease*[tiab] OR developmental illness*[tiab] OR developmental disabilit*[tiab] OR emotional disorder*[tiab] OR emotional disease*[tiab] OR emotional illness*[tiab] OR emotional health[tiab] OR behavioral disorder*[tiab] OR behavioral disease*[tiab] OR behavioral illness*[tiab] OR behavioral health[tiab] OR behavioral disabilit*[tiab] OR anxiety[tiab] OR depressive disorder*[tiab] OR depression[tiab] OR schizophrenia[tiab] OR bipolar disorder*[tiab] OR self-injur*[tiab] OR self-harm[tiab] OR suicide[tiab] OR autism[tiab] OR asperger[tiab] OR attention-deficit*hyperactivity disorder[tiab] OR ADHD[tiab] OR epilepsy[tiab] OR dementia[tiab] |
| #2 | “pacific islands”[MeSH Terms] OR pacific island*[tiab] OR pacifik island*[tiab] OR south pacific[tiab] OR south pacifik[tiab] OR samoa[tiab] OR cook island*[tiab] OR micronesia[tiab] OR fiji[tiab] OR french polynesia[tiab] OR guam[tiab] OR i-kiribati[tiab] OR kiribati[tiab] OR marshall island*[tiab] OR nauru[tiab] OR niue[tiab] OR northern mariana island*[tiab] OR palau[tiab] OR solomon island*[tiab] OR tokelau[tiab] OR tonga[tiab] OR tuvalu[tiab] OR vanuatu[tiab] OR pitcairn island*[tiab] OR new caledonia[tiab] OR papua new guinea[tiab] OR melanesia[tiab] OR (wallis[tiab] AND futuna[tiab]) OR gilbertese[tiab] OR gilbert island*[tiab] OR makin[tiab] OR butaritari[tiab] OR marakei[tiab] OR abaiang[tiab] OR tarawa[tiab] OR maiana[tiab] OR abemama[tiab] OR kuria[tiab] OR aranuka[tiab] OR nonouti[tiab] OR tabiteuea[tiab] OR beru[tiab] OR nikunau[tiab] OR onotoa[tiab] OR tamana[tiab] OR arorae[tiab] OR marshallese*[tiab] OR marshall island*[tiab] OR papuan*[tiab] OR choiseul*[tiab] OR shortland island*[tiab] OR new georgia island*[tiab] OR russell island*[tiab] OR tulagi*[tiab] OR malaita[tiab] OR maramasike[tiab] OR ulawa[tiab] OR owaraha[tiab] OR makira[tiab] OR guadalcanal[tiab] OR tongan*[tiab] OR ellice island*[tiab] OR nanumanga[tiab] OR niutao[tiab] OR niulakita[tiab] OR vanuatu*[tiab] |
| #3 | #1 AND #2 |
| #4 | Restrict to 2015 onwards |

### Grey literature

Grey literature will be identified through three complementary approaches: (1) Google search, (2) grey literature databases, and (3) targeted websites.

**Google search**

We will establish a search string for the PICTs of interest and put that string into the Google search box and then run (Table 2). As French is the official language in French Polynesia, New Caledonia, Vanuatu, and Wallis & Futuna, we will also use French key words to search for these four PICT.

**Table 2 Search string for Google search**

| (mental OR psychological OR psychiatric OR psychosis OR suicide) (pacific island OR pacifik island OR samoa OR cook island OR micronesia OR fiji OR french polynesia OR guam OR kiribati OR marshall island OR nauru OR new caledonia OR niue OR northern mariana island OR palau OR papua new guinea OR pitcairn island OR solomon island OR tokelau OR tonga OR tuvalu OR vanuatu OR wallis OR futuna) after:2015-01-01 (filetype:pdf OR filetype:ppt OR filetype:pptx OR filetype:doc OR filetype:docx) |
| --- |

**Grey literature databases**

We will systematically search grey literature databases that index policy reports, government documents, and institutional publications. The following databases and repositories will be included in our search: Policy Commons, Dimensions, OECD iLibrary, World Bank Documents and Reports, World Bank Open Knowledge Repository, and WHO Institutional Repository for Information Sharing (IRIS). To optimize search precision, we will develop tailored search strings specific to each database, ensuring that relevant documents related to mental health in PICTs are identified (see Table 3).

**Table 3 Search string for grey literature databases**

| **Grey literature databases** | **Search string** |
| --- | --- |
| Policy Commons | summary:("maladie mentale" OR "trouble mental" OR "troubles mentaux" OR "trouble psychiatrique" OR "santé mentale" OR "suicide" OR "psychose" OR "psychologique" OR "psychiatrique") AND summary:("Polynésie française" OR "Vanuatu" OR "Wallis-et-Futuna" OR "Nouvelle-Calédonie") |
| Dimensions | ("maladie mentale" OR "trouble mental" OR "troubles mentaux" OR "trouble psychiatrique" OR "santé mentale" OR "suicide" OR "psychose" OR "psychologique" OR "psychiatrique") AND ("Polynésie française" OR "Vanuatu" OR "Wallis-et-Futuna" OR "Nouvelle-Calédonie") |
| OECD iLibrary | ("maladie mentale" OR "trouble mental" OR "troubles mentaux" OR "trouble psychiatrique" OR "santé mentale" OR "suicide" OR "psychose" OR "psychologique" OR "psychiatrique") AND ("Polynésie française" OR "Vanuatu" OR "Wallis-et-Futuna" OR "Nouvelle-Calédonie") |
| World Bank Documents and Reports | ("maladie mentale" OR "trouble mental" OR "troubles mentaux" OR "trouble psychiatrique" OR "santé mentale" OR "suicide" OR "psychose" OR "psychologique" OR "psychiatrique") AND ("Polynésie française" OR "Vanuatu" OR "Wallis-et-Futuna" OR "Nouvelle-Calédonie") |
| World Bank Open Knowledge Repository | ("maladie mentale" OR "trouble mental" OR "troubles mentaux" OR "trouble psychiatrique" OR "santé mentale" OR "suicide" OR "psychose" OR "psychologique" OR "psychiatrique") AND ("Polynésie française" OR "Vanuatu" OR "Wallis-et-Futuna" OR "Nouvelle-Calédonie") |
| WHO IRIS | (Regional Office for the Western Pacific) AND [2015 TO 2025] AND French AND (“santé mentale” [MeSH] OR “troubles mentaux” [MeSH]) AND ("Polynésie française" OR "Vanuatu" OR "Wallis-et-Futuna" OR "Nouvelle-Calédonie") |

**Targeted website sources**

We will systematically search targeted website sources. The following sources will be included in our search: United Nations International Children's Emergency Fund (UNICEF) Pacific Islands, WHO Mental Health Atlas, Pacific Community, Pacific Island Health Officers Association (PIHOA), and Asian Development Bank. To optimize search precision, we will develop tailored search strings specific to each database, ensuring that relevant documents related to mental health in PICTs are identified (Table 4).

**Table 4 Search string for targeted website sources**

| **Targeted website sources** | **Search string** |
| --- | --- |
| WHO Mental Health Atlas | Latest atlas reports after 2015-01-01 in PICTs (“American Samoa” OR “Cook Islands” OR “Federated States of Micronesia” OR “Fiji” OR “French Polynesia” OR “Guam” OR “Kiribati” OR “Marshall Islands” OR “Nauru” OR “Niue” OR “Northern Mariana Islands” OR “Palau” OR “Samoa” OR “Solomon Islands” OR “Tokelau” OR “Tonga” OR “Tuvalu” OR “Vanuatu” OR “Pitcairn Islands” OR “Wallis and Futuna” OR “New Caledonia” OR “Papua New Guinea”) |
| UNICEF Pacific Islands | Mental health after 2015-01-01 |
| Pacific Community | (“mental ill” OR “mental illness” OR “mental disorders” OR “mental disease” OR “mental health” OR “suicide” OR “psychosis” OR “psychological” OR “psychiatric”) |
| Pacific Island Health Officers Association (PIHOA) | (“mental ill” OR “mental illness” OR “mental disorders” OR “mental disease” OR “mental health” OR “suicide” OR “psychosis” OR “psychological” OR “psychiatric”) AND (2015-2025) |
| Asian Development Bank | (“mental ill” OR “mental illness” OR “mental disorders” OR “mental disease” OR “mental health” OR “suicide” OR “psychosis” OR “psychological” OR “psychiatric”) AND (2015-2025) |

Consistent with our peer-reviewed literature search strategy, we will restrict grey literature searches to English-language documents for most PICTs. However, for New Caledonia, French Polynesia, Vanuatu, and Wallis and Futuna, where French is the official language, we will also search for and include grey literature in French to ensure the inclusion of all relevant national and regional publications.

##

## Study selection

Titles, abstracts, and full texts of retrieved records will be independently screened by two reviewers, with discrepancies resolved through discussion. Eligible studies will include quantitative and qualitative research reporting on mental health systems within PICTs. Excluded items will comprise conference abstracts, study protocols, pre-prints, studies conducted outside PICTs, and studies not reporting on outcomes of interest (e.g., leadership and governance, service delivery, health workforce, financing, access to essential medicines, health information systems). All reviewers will participate in a training session on inclusion and exclusion criteria. This session will enhance consistency in understanding criteria and screening process across the team.

**Data extraction**

To support standardised data collection, the review team will conduct interactive meetings to collaboratively develop a data extraction form, ensuring that all relevant domains and indicators are captured consistently. Data extraction will be conducted independently by two reviewers using an Excel spreadsheet (version 2408), capturing publication details (author, year, location, study design), participant characteristics (sample size, demographics), and the six domains of WHO Health Systems Building Blocks framework. Discrepancies will be resolved by consensus.

## Data synthesis and analysis

Findings will be synthesised using a narrative qualitative approach, allowing for a rich and descriptive presentation of the evidence. Results will be summarised at the country level, providing a tailored perspective on the mental health systems of each PICT. This approach will facilitate the identification of contextual factors, challenges, and opportunities specific to each setting, enhancing the relevance and applicability of the findings for policy and practice.

References

[1] Ali S, Williams O, Chang O, Shidhaye R, Hunter E, Charlson F. Mental health in the Pacific: Urgency and opportunity. Asia Pacific Viewpoint. 2020;61(3):537-50.

[2] Prasad N. Growth and social development in the Pacific Island countries. International Journal of Social Economics. 2008;35(12):930-50.

[3] Feeny S, Iamsiraroj S, McGillivray M. Growth and Foreign Direct Investment in the Pacific Island countries. Economic Modelling. 2014;37:332-9.

[4] Weir T, Dovey L, Orcherton D. Social and cultural issues raised by climate change in Pacific Island countries: an overview. Regional Environmental Change. 2017;17(4):1017-28.

[5] Tricco AC, Lillie E, Zarin W, O'Brien KK, Colquhoun H, Levac D, et al. PRISMA Extension for Scoping Reviews (PRISMA-ScR): Checklist and Explanation. Ann Intern Med. 2018;169(7):467-73.

**Supplementary Table 1** Preferred Reporting Items for Systematic reviews and Meta-Analyses extension for Scoping Reviews (PRISMA-ScR) Checklist

| **SECTION** | **ITEM** | **PRISMA-ScR CHECKLIST ITEM** | **REPORTED ON PAGE #** |
| --- | --- | --- | --- |
| **TITLE** | | | |
| Title | 1 | Identify the report as a scoping review. | 1 |
| **ABSTRACT** | | | |
| Structured summary | 2 | Provide a structured summary that includes (as applicable): background, objectives, eligibility criteria, sources of evidence, charting methods, results, and conclusions that relate to the review questions and objectives. | 3 |
| **INTRODUCTION** | | | |
| Rationale | 3 | Describe the rationale for the review in the context of what is already known. Explain why the review questions/objectives lend themselves to a scoping review approach. | 4-5 |
| Objectives | 4 | Provide an explicit statement of the questions and objectives being addressed with reference to their key elements (e.g., population or participants, concepts, and context) or other relevant key elements used to conceptualize the review questions and/or objectives. | 4-5 |
| **METHODS** | | | |
| Protocol and registration | 5 | Indicate whether a review protocol exists; state if and where it can be accessed (e.g., a Web address); and if available, provide registration information, including the registration number. | Study protocol can be found in the Supplementary File 1 |
| Eligibility criteria | 6 | Specify characteristics of the sources of evidence used as eligibility criteria (e.g., years considered, language, and publication status), and provide a rationale. | 5-6 |
| Information sources* | 7 | Describe all information sources in the search (e.g., databases with dates of coverage and contact with authors to identify additional sources), as well as the date the most recent search was executed. | 5-6 |
| Search | 8 | Present the full electronic search strategy for at least 1 database, including any limits used, such that it could be repeated. | 6-7, Supplementary Table 3 |
| Selection of sources of evidence† | 9 | State the process for selecting sources of evidence (i.e., screening and eligibility) included in the scoping review. | 6-7 |
| Data charting process‡ | 10 | Describe the methods of charting data from the included sources of evidence (e.g., calibrated forms or forms that have been tested by the team before their use, and whether data charting was done independently or in duplicate) and any processes for obtaining and confirming data from investigators. | 7, Supplementary Table 4 |
| Data items | 11 | List and define all variables for which data were sought and any assumptions and simplifications made. | 7, Supplementary Table 4 |
| Critical appraisal of individual sources of evidence§ | 12 | If done, provide a rationale for conducting a critical appraisal of included sources of evidence; describe the methods used and how this information was used in any data synthesis (if appropriate). | NA, 7 |
| Synthesis of results | 13 | Describe the methods of handling and summarizing the data that were charted. | 7-8 |
| **RESULTS** | | | |
| Selection of sources of evidence | 14 | Give numbers of sources of evidence screened, assessed for eligibility, and included in the review, with reasons for exclusions at each stage, ideally using a flow diagram. | 8, Figure 1 |
| Characteristics of sources of evidence | 15 | For each source of evidence, present characteristics for which data were charted and provide the citations. | 8-9, Table 1, Supplementary Table 5 |
| Critical appraisal within sources of evidence | 16 | If done, present data on critical appraisal of included sources of evidence (see item 12). | NA |
| Results of individual sources of evidence | 17 | For each included source of evidence, present the relevant data that were charted that relate to the review questions and objectives. | 8-9, Supplementary Tables 5-6 |
| Synthesis of results | 18 | Summarize and/or present the charting results as they relate to the review questions and objectives. | 9-13, Tables 1-2, Figures 2-3, Supplementary Tables 5-8 |
| **DISCUSSION** | | | |
| Summary of evidence | 19 | Summarize the main results (including an overview of concepts, themes, and types of evidence available), link to the review questions and objectives, and consider the relevance to key groups. | 13-18 |
| Limitations | 20 | Discuss the limitations of the scoping review process. | 18 |
| Conclusions | 21 | Provide a general interpretation of the results with respect to the review questions and objectives, as well as potential implications and/or next steps. | 18-19 |
| **FUNDING** | | | |
| Funding | 22 | Describe sources of funding for the included sources of evidence, as well as sources of funding for the scoping review. Describe the role of the funders of the scoping review. | 19 |

JBI = Joanna Briggs Institute; PRISMA-ScR = Preferred Reporting Items for Systematic reviews and Meta-Analyses extension for Scoping Reviews.

* Where *sources of evidence* (see second footnote) are compiled from, such as bibliographic databases, social media platforms, and Web sites.

† A more inclusive/heterogeneous term used to account for the different types of evidence or data sources (e.g., quantitative and/or qualitative research, expert opinion, and policy documents) that may be eligible in a scoping review as opposed to only studies. This is not to be confused with *information sources* (see first footnote).

‡ The frameworks by Arksey and O’Malley (6) and Levac and colleagues (7) and the JBI guidance (4, 5) refer to the process of data extraction in a scoping review as data charting*.*

§ The process of systematically examining research evidence to assess its validity, results, and relevance before using it to inform a decision. This term is used for items 12 and 19 instead of "risk of bias" (which is more applicable to systematic reviews of interventions) to include and acknowledge the various sources of evidence that may be used in a scoping review (e.g., quantitative and/or qualitative research, expert opinion, and policy document).

*From:* Tricco AC, Lillie E, Zarin W, O'Brien KK, Colquhoun H, Levac D, et al. PRISMA Extension for Scoping Reviews (PRISMAScR): Checklist and Explanation. Ann Intern Med. 2018;169:467–473. [doi: 10.7326/M18-0850](http://annals.org/aim/fullarticle/2700389/prisma-extension-scoping-reviews-prisma-scr-checklist-explanation).

**Supplementary Table 2** Country profile

| **Pacific island countries and territories** | **Political status** | **Income group** | **No. total population in 2024** | **Land area in 2023 (km^2^)** | **GDP per capita (USD)** | **Health expenditure in 2022 (% of GDP)** | **Human Development Index (HDI) rank in 2025** |
| --- | --- | --- | --- | --- | --- | --- | --- |
| American Samoa | Unincorporated territory of the United States | High | 46,765 | 200 | 13,709.1 (2022) | N/A | N/A |
| Cook Islands | Self-governing in free association with New Zealand | N/A | 13,729 | 240 | 25,750 (2023) | N/A | N/A |
| Federated States of Micronesia | Independent country in free association with the United States | Lower-middle | 113,160 | 700 | 2,930.3 (2024) | 10 | 149 |
| Fiji | Independent country | Upper-middle | 928,784 | 18,270 | 5,935.2 (2024) | 4 | 111 |
| French Polynesia | Overseas collectivity of France | High | 281,807 | 3,471 | 20,846.7 (2024) | N/A | N/A |
| Guam | Unincorporated territory of the United States | High | 167,777 | 540 | 34,628.0 (2022) | N/A | N/A |
| Kiribati | Independent country | Lower-middle | 134,518 | 810 | 2,041.9 (2024) | 10 | 140 |
| Marshall Islands | Independent country in free association with the United States | Upper-middle | 37,548 | 180 | 5,811.0 (2024) | 12 | 108 |
| Nauru | Independent country | High | 11,947 | 20 | 8,571.0 (2024) | 18 | 124 |
| New Caledonia | Special collectivity of France | High | 292,639 | 18,280 | 30,858.7 (2015) | N/A | N/A |
| Niue | Self-governing in free association with New Zealand | N/A | 1,819 | 260 | 16,589 (2024) | N/A | N/A |
| Northern Mariana Islands | Commonwealth in political union with the United States | High | 44,278 | 25,220 | 19,461.9 (2022) | N/A | N/A |
| Palau | Independent country in free association with the United States | High | 17,695 | 460 | 13,128.1 (2023) | 14 | 84 |
| Papua New Guinea | Independent country | Lower-middle | 10,576,502 | 452,860 | 2,564.7 (2024) | 3 | 160 |
| Pitcairn Islands | British overseas territory (United Kingdom) | N/A | 50 | 47 | N/A | N/A | N/A |
| Samoa | Independent country | Upper-middle | 218,019 | 2,780 | 4,880.5 (2024) | 6 | 122 |
| Solomon Islands | Independent country | Lower-middle | 819,198 | 27,990 | 1,806.4 (2024) | 5 | 156 |
| Tokelau | Dependent territory of New Zealand | N/A | 2,506 | 10 | 4,962 (2021) | N/A | N/A |
| Tonga | Independent country | Upper-middle | 104,175 | 720 | 4,680.3 (2023) | 8 | 92 |
| Tuvalu | Independent country | Upper-middle | 9,646 | 30 | 4,773.3 (2023) | 18 | 129 |
| Vanuatu | Independent country | Lower-middle | 327,777 | 12,190 | 2,697.1 (2024) | 4 | 146 |
| Wallis & Futuna | Overseas collectivity of France | N/A | 11,277 | 140 | 11,427 (2015) | N/A | N/A |

GDP, Gross Domestic Product; UDS, United States dollar.

Note: Political status was obtained from publicly available country and territory descriptions on Wikipedia: <https://www.wikipedia.org/>.

Country income groups follow the World Bank country and lending group classification (2025): <https://datahelpdesk.worldbank.org/knowledgebase/articles/906519-world-bank-country-and-lending-groups>.

Total population estimates for 2024 were obtained from the World Bank: <https://data.worldbank.org/indicator/SP.POP.TOTL>; where unavailable, data were supplemented using Worldometer for the Cook Islands, Niue, Tokelau, and Wallis & Futuna: <https://www.worldometers.info/population/>, and the Pacific Community (SPC) for the Pitcairn Islands: <https://www.spc.int/updates/blog/did-you-know/2025/07/stat-of-the-week-50-people-is-the-estimated-population-of-the>.

Land area (km^2^) was obtained from the World Bank: <https://data.worldbank.org/indicator/AG.LND.TOTL.K2>; where unavailable, data for the Cook Islands, Niue, Tokelau, and Wallis & Futuna were sourced from the Pacific Community (SPC): <https://www.spc.int/our-members/>.

GDP per capita in 2024 (constant 2015 USD) was obtained from the World Bank: <https://data.worldbank.org/indicator/NY.GDP.PCAP.KD>; where unavailable, estimates were supplemented using United Nations data for the Cook Islands: <https://data.un.org/Data.aspx?d=SNAAMA&f=grID:101;currID:USD;pcFlag:1;crID:184>, and SPC member profiles for Niue, Tokelau, and Wallis & Futuna: <https://www.spc.int/our-members/>.

Current health expenditure (% of GDP) for 2022 was obtained from the World Bank: <https://data.worldbank.org/indicator/SH.XPD.CHEX.GD.ZS>.

Human Development Index (HDI) rank was obtained from United Nations Development Programme: https://hdr.undp.org/data-center/country-insights#/ranks

**Supplementary Table 3** Search strategy

| **Data source** | **Language** | **Search strategy** |
| --- | --- | --- |
| PubMed | English | (“mental health”[MeSH Terms] OR “mental disorders”[MeSH Terms] OR “psychological well-being”[MeSH Terms] OR “anxiety”[MeSH Terms] OR “depression”[MeSH Terms] OR “euphoria”[MeSH Terms] OR “stress, psychological”[MeSH Terms] OR “self-Injurious behavior”[MeSH Terms] OR “epilepsy”[MeSH Terms] OR mental disorder*[tiab] OR mental disease*[tiab] OR mental illness*[tiab] OR mental health[tiab] OR mental well*being[tiab] OR psychiatric disorder*[tiab] OR psychiatric disease*[tiab] OR psychiatric illness*[tiab] OR psychiatric health[tiab] OR psychiatric well-being[tiab] OR psychosis[tiab] OR psychological disorder*[tiab] OR psychological disease*[tiab] OR psychological illness*[tiab] OR psychological health[tiab] OR psychological well*being[tiab] OR psychotic disorder*[tiab] OR psychotic disease*[tiab] OR psychotic illness*[tiab] OR psychotic health[tiab] OR psychotic well*being[tiab] OR developmental disorder*[tiab] OR developmental disease*[tiab] OR developmental illness*[tiab] OR developmental disabilit*[tiab] OR emotional disorder*[tiab] OR emotional disease*[tiab] OR emotional illness*[tiab] OR emotional health[tiab] OR behavioral disorder*[tiab] OR behavioral disease*[tiab] OR behavioral illness*[tiab] OR behavioral health[tiab] OR behavioral disabilit*[tiab] OR anxiety[tiab] OR depressive disorder*[tiab] OR depression[tiab] OR schizophrenia[tiab] OR bipolar disorder*[tiab] OR self-injur*[tiab] OR self-harm[tiab] OR suicide[tiab] OR autism[tiab] OR asperger[tiab] OR attention-deficit*hyperactivity disorder[tiab] OR ADHD[tiab] OR epilepsy[tiab] OR dementia[tiab]) AND (“pacific islands”[MeSH Terms] OR pacific island*[tiab] OR pacifik island*[tiab] OR south pacific[tiab] OR south pacifik[tiab] OR samoa[tiab] OR cook island*[tiab] OR micronesia[tiab] OR fiji[tiab] OR french polynesia[tiab] OR guam[tiab] OR i-kiribati[tiab] OR kiribati[tiab] OR marshall island*[tiab] OR nauru[tiab] OR niue[tiab] OR northern mariana island*[tiab] OR palau[tiab] OR solomon island*[tiab] OR tokelau[tiab] OR tonga[tiab] OR tuvalu[tiab] OR vanuatu[tiab] OR pitcairn island*[tiab] OR new caledonia[tiab] OR papua new guinea[tiab] OR melanesia[tiab] OR (wallis[tiab] AND futuna[tiab]) OR gilbertese[tiab] OR gilbert island*[tiab] OR makin[tiab] OR butaritari[tiab] OR marakei[tiab] OR abaiang[tiab] OR tarawa[tiab] OR maiana[tiab] OR abemama[tiab] OR kuria[tiab] OR aranuka[tiab] OR nonouti[tiab] OR tabiteuea[tiab] OR beru[tiab] OR nikunau[tiab] OR onotoa[tiab] OR tamana[tiab] OR arorae[tiab] OR marshallese*[tiab] OR marshall island*[tiab] OR papuan*[tiab] OR choiseul*[tiab] OR shortland island*[tiab] OR new georgia island*[tiab] OR russell island*[tiab] OR tulagi*[tiab] OR malaita[tiab] OR maramasike[tiab] OR ulawa[tiab] OR owaraha[tiab] OR makira[tiab] OR guadalcanal[tiab] OR tongan*[tiab] OR ellice island*[tiab] OR nanumanga[tiab] OR niutao[tiab] OR niulakita[tiab] OR vanuatu*[tiab]) AND (2015-2025) |
| EMBASE | English | (('mental health'/exp OR 'mental disease'/exp OR 'psychological well-being'/exp OR 'anxiety'/exp OR 'depression'/exp OR 'euphoria'/exp OR 'psychological stress'/exp OR 'self injurious behavior'/exp OR 'epilepsy'/exp) OR (mental disorder* OR mental disease* OR mental illness* OR mental health OR mental well*being OR psychiatric disorder* OR psychiatric disease* OR psychiatric illness* OR psychiatric health OR psychiatric well-being OR psychosis OR psychological disorder* OR psychological disease* OR psychological illness* OR psychological health OR psychological well*being OR psychotic disorder* OR psychotic disease* OR psychotic illness* OR psychotic health OR psychotic well*being OR developmental disorder* OR developmental disease* OR developmental illness* OR developmental disabilit* OR emotional disorder* OR emotional disease* OR emotional illness* OR emotional health OR behavioral disorder* OR behavioral disease* OR behavioral illness* OR behavioral health OR behavioral disabilit* OR anxiety OR depressive disorder* OR depression OR schizophrenia OR bipolar disorder* OR self-injur* OR self-harm OR suiciOR autism OR asperger OR 'attention deficit hyperactivity disorder' OR ADHD OR epilepsy OR dementia ").ti,ab,kw.) AND (('pacific islands'/exp) OR pacific island* OR pacifik island* OR south pacific OR south pacifik OR samoa OR cook island* OR micronesia OR fiji OR french polynesia OR guam OR kiribati OR marshall island* OR nauru OR niue OR northern mariana island* OR palau OR solomon island* OR tokelau OR tonga OR tuvalu OR vanuatu OR pitcairn island* OR new caledonia OR papua new guinea OR melanesia OR (wallis AND futuna) OR gilbertese OR gilbert island* OR makin OR butaritari OR marakei OR abaiang OR tarawa OR maiana OR abemama OR kuria OR aranuka OR nonouti OR tabiteuea OR beru OR nikunau OR onotoa OR tamana OR arorae OR marshallese* OR papuan* OR choiseul* OR shortland island* OR new georgia island* OR russell island* OR tulagi* OR malaita OR maramasike OR ulawa OR owaraha OR makira OR guadalcanal OR tongan* OR ellice island* OR nanumanga OR niutao OR niulakita OR vanuatu*").ti,ab,kw.) AND [2015-2025]/py |
| CINAHL | English | ((MH "Mental Health") OR (MH "Mental Disorders+") OR (MH "Anxiety Disorders+") OR (MH "Depression+") OR (MH "Psychological Stress") OR (MH "Self-Injurious Behavior") OR (MH "Epilepsy") OR TX ("mental disorder*" OR "mental disease*" OR "mental illness*" OR "mental health" OR "mental well*being" OR "psychiatric disorder*" OR "psychiatric disease*" OR "psychiatric illness*" OR "psychiatric health" OR "psychiatric well-being" OR psychosis OR "psychological disorder*" OR "psychological disease*" OR "psychological illness*" OR "psychological health" OR "psychological well*being" OR "psychotic disorder*" OR "psychotic disease*" OR "psychotic illness*" OR "psychotic health" OR "psychotic well*being" OR "developmental disorder*" OR "developmental disease*" OR "developmental illness*" OR "developmental disabilit*" OR "emotional disorder*" OR "emotional disease*" OR "emotional illness*" OR "emotional health" OR "behavioral disorder*" OR "behavioral disease*" OR "behavioral illness*" OR "behavioral health" OR "behavioral disabilit*" OR anxiety OR "depressive disorder*" OR depression OR schizophrenia OR "bipolar disorder*" OR self-injur* OR self-harm OR suiciOR autism OR asperger OR "attention-deficit*hyperactivity disorder" OR ADHD OR epilepsy OR dementia)) AND ((MH "Pacific Islands+") OR TX ("pacific island*" OR "pacifik island*" OR "south pacific" OR "south pacifik" OR samoa OR "cook island*" OR micronesia OR fiji OR "french polynesia" OR guam OR kiribati OR i-kiribati OR "marshall island*" OR nauru OR niue OR "northern mariana island*" OR palau OR "solomon island*" OR tokelau OR tonga OR tuvalu OR vanuatu OR "pitcairn island*" OR "new caledonia" OR "papua new guinea" OR melanesia OR (wallis AND futuna) OR gilbertese OR "gilbert island*" OR makin OR butaritari OR marakei OR abaiang OR tarawa OR maiana OR abemama OR kuria OR aranuka OR nonouti OR tabiteuea OR beru OR nikunau OR onotoa OR tamana OR arorae OR marshallese* OR papuan* OR choiseul* OR "shortland island*" OR "new georgia island*" OR "russell island*" OR tulagi* OR malaita OR maramasike OR ulawa OR owaraha OR makira OR guadalcanal OR tongan* OR "ellice island*" OR nanumanga OR niutao OR niulakita OR vanuatu*)) AND (2015-2025) |
| PsycINFO | English | (“mental disorder*” OR “mental disease*” OR “mental illness*” OR “mental health” OR “mental well*being” OR “psychiatric disorder*” OR “psychiatric disease*” OR “psychiatric illness*” OR “psychosis” OR “psychological disorder*” OR “psychological disease*” “psychological stress” OR OR “psychological illness*” OR “psychotic disorder*” OR “developmental disorder*” OR “emotional disorder*” OR “behavioral disorder*” OR “anxiety” OR “depression” OR “depressive disorder*” OR “bipolar disorder*” OR “autism” OR “asperger” OR “attention-deficit*hyperactivity disorder” OR ADHD OR “epilepsy” OR “dementia” OR “self-Injurious behavior”).ab,mh,ti. AND ( “pacific island*” OR “pacifik island*” OR “south pacific” OR “samoa” OR “cook island*” OR “micronesia” OR “fiji” OR “guam” OR “kiribati” OR “marshall island*” OR “nauru” OR “niue” OR “palau” OR “solomon island*” OR “tokelau” OR “tonga” OR “tuvalu” OR “vanuatu” OR “pitcairn island*” OR “new caledonia” OR “papua new guinea” OR “melanesia” OR “marshallese*” OR “wallis” AND "futuna").ab,mh,ti. AND (2015-2025) |
| PAIS Index | English | Searched for: ("mental health" OR "mental disorders" OR "psychological well-being" OR "anxiety" OR "depression" OR "euphoria" OR "stress, psychological" OR "self-Injurious behavior" OR "epilepsy" OR "mental disorder*" OR "mental disease*" OR "mental illness*" OR "mental well*being" OR "psychiatric disorder*" OR "psychiatric disease*" OR "psychiatric illness*" OR "psychiatric health" OR "psychiatric well-being" OR "psychosis" OR "psychological disorder*" OR "psychological disease*" OR "psychological illness*" OR "psychological health" OR "psychological well*being" OR "psychotic disorder*" OR "psychotic disease*" OR "psychotic illness*" OR "psychotic health" OR "psychotic well*being" OR "developmental disorder*" OR "developmental disease*" OR "developmental illness*" OR "developmental disabilit*" OR "emotional disorder*" OR "emotional disease*" OR "emotional illness*" OR "emotional health" OR "behavioral disorder*" OR "behavioral disease*" OR "behavioral illness*" OR "behavioral health" OR "behavioral disabilit*" OR "anxiety" OR "depressive disorder*" OR "depression" OR "schizophrenia" OR "bipolar disorder*" OR "self-injur*" OR "self-harm" OR "suicide" OR "autism" OR "asperger" OR "attention-deficit*hyperactivity disorder" OR "ADHD" OR "epilepsy" OR "dementia") "pacific islands" OR "pacific island*" OR "pacifik island*" OR "south pacific" OR "south pacifik" OR "samoa" OR "cook island*" OR "micronesia" OR "fiji" OR "french polynesia" OR "guam" OR "i-kiribati" OR "kiribati" OR "marshall island*" OR "nauru" OR "niue" OR "northern mariana island*" OR "palau" OR "solomon island*" OR "tokelau" OR "tonga" OR "tuvalu" OR "vanuatu" OR "pitcairn island*" OR "new caledonia" OR "papua new guinea" OR "melanesia" OR ("wallis" AND "futuna") OR "gilbertese" OR "gilbert island*" OR "makin" OR "butaritari" OR "marakei" OR "abaiang" OR "tarawa" OR "maiana" OR "abemama" OR "kuria" OR "aranuka" OR "nonouti" OR "tabiteuea" OR "beru" OR "nikunau" OR "onotoa" OR "tamana" OR "arorae" OR "marshallese*" OR "papuan*" OR "choiseul*" OR "shortland island*" OR "new georgia island*" OR "russell island*" OR "tulagi*" OR "malaita" OR "maramasike" OR "ulawa" OR "owaraha" OR "makira" OR "guadalcanal" OR "tongan*" OR "ellice island*" OR "nanumanga" OR "niutao" OR "niulakita" OR "vanuatu*") AND pd(>20150101) |
| Web of Science | English | TS=("mental disorder*" OR "mental disease*" OR "mental illness*" OR "mental health" OR "mental well*being" OR "psychiatric disorder*" OR "psychiatric disease*" OR "psychiatric illness*" OR "psychiatric health" OR "psychiatric well-being" OR "psychosis" OR "psychological disorder*" OR "psychological disease*" OR "psychological illness*" OR "psychological health" OR "psychological well*being" OR "psychotic disorder*" OR "psychotic disease*" OR "psychotic illness*" OR "psychotic health" OR "psychotic well*being" OR "developmental disorder*" OR "developmental disease*" OR "developmental illness*" OR "developmental disabilit*" OR "emotional disorder*" OR "emotional disease*" OR "emotional illness*" OR "emotional health" OR "behavioral disorder*" OR "behavioral disease*" OR "behavioral illness*" OR "behavioral health" OR "behavioral disabilit*" OR "anxiety" OR "depressive disorder*" OR "depression" OR "schizophrenia" OR "bipolar disorder*" OR "self-injur*" OR "self-harm" OR "suicide" OR "autism" OR "asperger" OR "attention-deficit*hyperactivity disorder" OR "ADHD" OR "epilepsy" OR "dementia"") AND TS=("pacific island*" OR "pacifik island*" OR "south pacific" OR "south pacifik" OR "samoa" OR "cook island*" OR "micronesia" OR "fiji" OR "french polynesia" OR "guam" OR "kiribati" OR "marshall island*" OR "nauru" OR "niue" OR "northern mariana island*" OR "palau" OR "solomon island*" OR "tokelau" OR "tonga" OR "tuvalu" OR "vanuatu" OR "pitcairn island*" OR "new caledonia" OR "papua new guinea" OR "melanesia" OR ("wallis" AND "futuna") OR "gilbert island*" OR "marshallese*" OR "papuan*" OR "choiseul*" OR "shortland island*" OR "new georgia island*" OR "russell island*" OR "tulagi*" OR "malaita" OR "maramasike" OR "ulawa"" OR "owaraha" OR "makira" OR "guadalcanal" OR "ellice island*" OR "nanumanga" OR "niutao" OR "niulakita") AND Timespan: 2015 to 2025 |
| WHO Western Pacific Region Index Medicus | English | ("mental health" OR "mental disorders" OR "psychological well-being" OR "anxiety" OR "depression" OR "euphoria" OR "stress, psychological" OR "self-Injurious behavior" OR "epilepsy" OR "mental disorder*" OR "mental disease*" OR "mental illness*" OR "mental health" OR "mental well*being" OR "psychiatric disorder*" OR "psychiatric disease*" OR "psychiatric illness*" OR "psychiatric health" OR "psychiatric well-being" OR psychosis OR "psychological disorder*" OR "psychological disease*" OR "psychological illness*" OR "psychological health" OR "psychological well*being" OR "psychotic disorder*" OR "psychotic disease*" OR "psychotic illness*" OR "psychotic health" OR "psychotic well*being" OR "developmental disorder*" OR "developmental disease*" OR "developmental illness*" OR "developmental disabilit*" OR "emotional disorder*" OR "emotional disease*" OR "emotional illness*" OR "emotional health" OR "behavioral disorder*" OR "behavioral disease*" OR "behavioral illness*" OR "behavioral health" OR "behavioral disabilit*" OR anxiety OR "depressive disorder*" OR depression OR schizophrenia OR "bipolar disorder*" OR self-injur* OR self-harm OR suicide OR autism OR asperger OR "attention-deficit*hyperactivity disorder" OR ADHD OR epilepsy OR dementia) AND ("pacific islands" OR "pacific island*" OR "pacifik island*" OR "south pacific" OR "south pacifik" OR samoa OR "cook island*" OR micronesia OR fiji OR "french polynesia" OR guam OR i-kiribati OR kiribati OR "marshall island*" OR nauru OR niue OR "northern mariana island*" OR palau OR "solomon island*" OR tokelau OR tonga OR tuvalu OR vanuatu OR "pitcairn island*" OR "new caledonia" OR "papua new guinea" OR melanesia OR (wallis AND futuna) OR gilbertese OR "gilbert island*" OR makin OR butaritari OR marakei OR abaiang OR tarawa OR maiana OR abemama OR kuria OR aranuka OR nonouti OR tabiteuea OR beru OR nikunau OR onotoa OR tamana OR arorae OR marshallese* OR "marshall island*" OR papuan* OR choiseul* OR "shortland island*" OR "new georgia island*" OR "russell island*" OR tulagi* OR malaita OR maramasike OR ulawa OR owaraha OR makira OR guadalcanal OR tongan* OR "ellice island*" OR nanumanga OR niutao OR niulakita OR vanuatu*) AND (year_cluster:[2015 TO 2025]) |
| HAL (Hyper Articles en Ligne) | English | (“mental health” OR “mental disorders” OR “psychological well-being” OR “anxiety” OR “depression” OR “euphoria” OR “stress, psychological” OR “self-Injurious behavior” OR “epilepsy” OR mental disorder* OR mental disease* OR mental illness* OR mental health OR mental well*being OR psychiatric disorder* OR psychiatric disease* OR psychiatric illness* OR psychiatric health OR psychiatric well-being OR psychosis OR psychological disorder* OR psychological disease* OR psychological illness* OR psychological health OR psychological well*being OR psychotic disorder* OR psychotic disease* OR psychotic illness* OR psychotic health OR psychotic well*being OR developmental disorder* OR developmental disease* OR developmental illness* OR developmental disabilit* OR emotional disorder* OR emotional disease* OR emotional illness* OR emotional health OR behavioral disorder* OR behavioral disease* OR behavioral illness* OR behavioral health OR behavioral disabilit* OR anxiety OR depressive disorder* OR depression OR schizophrenia OR bipolar disorder* OR self-injur* OR self-harm OR suiciOR autism OR asperger OR attention-deficit*hyperactivity disorder OR ADHD OR epilepsy OR dementia) AND (“pacific islands” OR pacific island* OR pacifik island* OR south pacific OR south pacifik OR samoa OR cook island* OR micronesia OR fiji OR french polynesia OR guam OR i-kiribati OR kiribati OR marshall island* OR nauru OR niue OR northern mariana island* OR palau OR solomon island* OR tokelau OR tonga OR tuvalu OR vanuatu OR pitcairn island* OR new caledonia OR papua new guinea OR melanesia OR (wallis AND futuna) OR gilbertese OR gilbert island* OR makin OR butaritari OR marakei OR abaiang OR tarawa OR maiana OR abemama OR kuria OR aranuka OR nonouti OR tabiteuea OR beru OR nikunau OR onotoa OR tamana OR arorae OR marshallese* OR marshall island* OR papuan* OR choiseul* OR shortland island* OR new georgia island* OR russell island* OR tulagi* OR malaita OR maramasike OR ulawa OR owaraha OR makira OR guadalcanal OR tongan* OR ellice island* OR nanumanga OR niutao OR niulakita OR vanuatu*) AND (Year: 2015-2025) |
| HAL (Hyper Articles en Ligne) | French | (« santé mentale » OR « troubles mentaux » OR « bien-être psychologique » OR « anxiété » OR « dépression » OR « manie » OR « hypomanie » OR « stress psychologique » OR « comportement autodestructeur » OR « épilepsie » OR « trouble mental* » OR « maladie mentale* » OR « trouble psychiatrique* » OR « maladie psychiatrique* » OR « santé mentale » OR « bien*être mental » OR « bien-être psychologique » OR psychose OR « trouble psychologique* » OR « maladie psychologique* » OR « trouble psychotique* » OR « maladie psychotique* » OR « santé psychologique » OR « bien*être psychologique » OR « trouble du développement* » OR « maladie du développement* » OR « handicap développemental* » OR « trouble émotionnel* » OR « maladie émotionnelle* » OR « santé émotionnelle » OR « trouble du comportement* ») AND (« îles du Pacifique » OR « îles du pacifique » OR « pacifik island* » OR « Pacifique Sud » OR samoa OR « îles cook » OR micronésie OR fidji OR « polynésie française » OR guam OR i-kiribati OR kiribati OR « îles marshall » OR nauru OR nioué OR « îles mariannes du nord » OR palaos OR « îles salomon » OR tokelau OR tonga OR tuvalu OR vanuatu OR « îles pitcairn » OR « nouvelle-calédonie » OR « papouasie-nouvelle-guinée » OR mélanésie OR (wallis AND futuna) OR gilbertin OR « îles gilbert » OR makin OR butaritari OR marakei OR abaiang OR tarawa OR maiana OR abemama OR kuria OR aranuka OR nonouti OR tabiteuea OR beru OR nikunau OR onotoa OR tamana OR arorae OR marshallaise* OR « îles marshall » OR papouan* OR choiseul* OR « îles shortland » OR « îles new georgia » OR « îles russell » OR tulagi* OR malaita OR maramasike OR ulawa OR owaraha OR makira OR guadalcanal OR tongien* OR « îles ellice » OR nanumanga OR niutao OR niulakita OR vanuatuan*) AND (Year: 2015-2025) |
| Google search | English | (mental OR psychological OR psychiatric OR psychosis OR suicide) (pacific island OR pacifik island OR samoa OR cook island OR micronesia OR fiji OR french polynesia OR guam OR kiribati OR marshall island OR nauru OR new caledonia OR niue OR northern mariana island OR palau OR papua new guinea OR pitcairn island OR solomon island OR tokelau OR tonga OR tuvalu OR vanuatu OR wallis OR futuna) after:2015-01-01 (filetype:pdf OR filetype:ppt OR filetype:pptx OR filetype:doc OR filetype:docx) |
| Google search | French | Search string: (mentale OR psychologique OR psychiatrique OR psychose OR suicide) (polynésie française OR vanuatu OR wallis-et-futuna OR nouvelle-calédonie) after:2015-01-01 (filetype:pdf OR filetype:ppt OR filetype:pptx OR filetype:doc OR filetype:docx) |
| Policy Commons | English | summary:(“mental ill” OR “mental illness” OR “mental disorders” OR “mental disease” OR “mental health” OR “suicide” OR “psychosis” OR “psychological” OR “psychiatric”) AND summary:(“American Samoa” OR “Cook Islands” OR “Federated States of Micronesia” OR “Fiji” OR “French Polynesia” OR “Guam” OR “Kiribati” OR “Marshall Islands” OR “Nauru” OR “Niue” OR “Northern Mariana Islands” OR “Palau” OR “Samoa” OR “Solomon Islands” OR “Tokelau” OR “Tonga” OR “Tuvalu” OR “Vanuatu” OR “Pitcairn Islands” OR “Wallis and Futuna” OR “New Caledonia” OR “Papua New Guinea”) AND (Year: 2015-2025) |
| Policy Commons | French | summary:("maladie mentale" OR "trouble mental" OR "troubles mentaux" OR "trouble psychiatrique" OR "santé mentale" OR "suicide" OR "psychose" OR "psychologique" OR "psychiatrique") AND summary:("Polynésie française" OR "Vanuatu" OR "Wallis-et-Futuna" OR "Nouvelle-Calédonie") AND (Year: 2015-2025) |
| Dimensions | English | (“mental disorder*” OR “mental disease*” OR “mental illness*” OR “mental health” OR “mental well*being” OR “psychiatric disorder*” OR “psychiatric disease*” OR “psychiatric illness*” OR “psychiatric health” OR “psychiatric well-being” OR “psychosis” OR “psychological disorder*” OR “psychological disease*” OR “psychological illness*” OR “psychological health” OR “psychological well*being” OR “psychotic disorder*” OR “psychotic disease*” OR “psychotic illness*” OR “psychotic health” OR “psychotic well*being” OR “developmental disorder*” OR “developmental disease*” OR “developmental illness*” OR “developmental disabilit*” OR “emotional disorder*” OR “emotional disease*” OR “emotional illness*” OR “emotional health” OR “behavioral disorder*” OR “behavioral disease*” OR “behavioral illness*” OR “behavioral health” OR “behavioral disabilit*” OR “anxiety” OR “depressive disorder*” OR “depression” OR “schizophrenia” OR “bipolar disorder*” OR “self-injur*” OR “self-harm” OR “suicide” OR “autism” OR “asperger” OR “attention-deficit*hyperactivity disorder” OR “ADHD” OR “epilepsy” OR “dementia”) AND (“pacific island*” OR “pacifik island*” OR “south pacific” OR “south pacifik” OR “samoa” OR “cook island*” OR “micronesia” OR “fiji” OR “french polynesia” OR “guam” OR “i-kiribati” OR “kiribati” OR “marshall island*” OR “nauru” OR “niue” OR “northern mariana island*” OR “palau” OR “solomon island*” OR “tokelau” OR “tonga” OR “tuvalu” OR “vanuatu” OR “pitcairn island*” OR “new caledonia” OR “papua new guinea” OR “melanesia” OR (“wallis” AND “futuna”) OR “gilbertese” OR “gilbert island*” OR “makin” OR “butaritari” OR “marakei” OR “abaiang” OR “tarawa” OR “maiana” OR “abemama” OR “kuria” OR “aranuka” OR “nonouti” OR “tabiteuea” OR “beru” OR “nikunau” OR “onotoa” OR “tamana” OR “arorae” OR “marshallese*” OR “marshall island*” OR “papuan*” OR “choiseul*” OR “shortland island*” OR “new georgia island*” OR “russell island*” OR “tulagi*” OR “malaita” OR “maramasike” OR “ulawa” OR “owaraha” OR “makira” OR “guadalcanal” OR “tongan*” OR “ellice island*” OR “nanumanga” OR “niutao” OR “niulakita” OR “vanuatu*”) AND (2025 OR 2024 OR 2023 OR 2022 OR 2021 OR 2020 OR 2019 OR 2018 OR 2017 OR 2016 OR 2015: Publication Year) |
| Dimensions | French | (“trouble mental*” OR “maladie mentale*” OR “maladie mentale*” OR “santé mentale” OR “bien-être mental” OR “trouble psychiatrique*” OR “maladie psychiatrique*” OR “maladie psychiatrique*” OR “santé psychiatrique” OR “bien-être psychiatrique” OR “psychose” OR “trouble psychologique*” OR “maladie psychologique*” OR “maladie psychologique*” OR “santé psychologique” OR “bien-être psychologique” OR “trouble psychotique*” OR “maladie psychotique*” OR “maladie psychotique*” OR “santé psychotique” OR “bien-être psychotique” OR “trouble du développement*” OR “maladie du développement*” OR “maladie du développement*” OR “déficience du développement*” OR “trouble émotionnel*” OR “maladie émotionnelle*” OR “maladie émotionnelle*” OR “santé émotionnelle” OR “trouble du comportement*” OR “maladie du comportement*” OR “maladie du comportement*” OR “trouble du comportement santé” OR “handicap comportemental*” OR “anxiété” OR “trouble dépressif*” OR “dépression” OR “schizophrénie” OR “trouble bipolaire*” OR “automutilation*” OR “automutilation” OR “suicide” OR “autisme” OR “asperger” OR “trouble déficitaire l’attention*avec hyperactivité” OR “TDAH” OR “épilepsie” OR “démence”) AND (“Polynésie française” OR “Vanuatu” OR “Wallis-et-Futuna” OR “Nouvelle-Calédonie”) AND (2025 OR 2024 OR 2023 OR 2022 OR 2021 OR 2020 OR 2019 OR 2018 OR 2017 OR 2016 OR 2015: Publication Year) |
| OECD iLibrary | English | (“mental ill” OR “mental illness” OR “mental disorders” OR “mental disease” OR “mental health” OR “suicide” OR “psychosis” OR “psychological” OR “psychiatric”) AND (“American Samoa” OR “Cook Islands” OR “Federated States of Micronesia” OR “Fiji” OR “French Polynesia” OR “Guam” OR “Kiribati” OR “Marshall Islands” OR “Nauru” OR “Niue” OR “Northern Mariana Islands” OR “Palau” OR “Samoa” OR “Solomon Islands” OR “Tokelau” OR “Tonga” OR “Tuvalu” OR “Vanuatu” OR “Pitcairn Islands” OR “Wallis and Futuna” OR “New Caledonia” OR “Papua New Guinea”) |
| OECD iLibrary | French | ("maladie mentale" OR "trouble mental" OR "troubles mentaux" OR "trouble psychiatrique" OR "santé mentale" OR "suicide" OR "psychose" OR "psychologique" OR "psychiatrique") AND ("Polynésie française" OR "Vanuatu" OR "Wallis-et-Futuna" OR "Nouvelle-Calédonie") |
| World Bank Documents and Reports | English | (“mental ill” OR “mental illness” OR “mental disorders” OR “mental disease” OR “mental health” OR “suicide” OR “psychosis” OR “psychological” OR “psychiatric”) AND (“American Samoa” OR “Cook Islands” OR “Federated States of Micronesia” OR “Fiji” OR “French Polynesia” OR “Guam” OR “Kiribati” OR “Marshall Islands” OR “Nauru” OR “Niue” OR “Northern Mariana Islands” OR “Palau” OR “Samoa” OR “Solomon Islands” OR “Tokelau” OR “Tonga” OR “Tuvalu” OR “Vanuatu” OR “Pitcairn Islands” OR “Wallis and Futuna” OR “New Caledonia” OR “Papua New Guinea”) AND Filter: (Language: English, French), (Years: 2015-2025), (Region: East Asia and Pacific) |
| World Bank Documents and Reports | French | ("maladie mentale" OR "trouble mental" OR "troubles mentaux" OR "trouble psychiatrique" OR "santé mentale" OR "suicide" OR "psychose" OR "psychologique" OR "psychiatrique") AND ("Polynésie française" OR "Vanuatu" OR "Wallis-et-Futuna" OR "Nouvelle-Calédonie") AND Filter: (Language: English, French), (Years: 2015-2025), (Region: East Asia and Pacific) |
| World Bank Open Knowledge Repository | English | (“mental ill” OR “mental illness” OR “mental disorders” OR “mental disease” OR “mental health” OR “suicide” OR “psychosis” OR “psychological” OR “psychiatric”) AND (“American Samoa” OR “Cook Islands” OR “Federated States of Micronesia” OR “Fiji” OR “French Polynesia” OR “Guam” OR “Kiribati” OR “Marshall Islands” OR “Nauru” OR “Niue” OR “Northern Mariana Islands” OR “Palau” OR “Samoa” OR “Solomon Islands” OR “Tokelau” OR “Tonga” OR “Tuvalu” OR “Vanuatu” OR “Pitcairn Islands” OR “Wallis and Futuna” OR “New Caledonia” OR “Papua New Guinea”) AND Filter: (Language: English, French), (Years: 2015-2025), (Region: East Asia and Pacific) |
| World Bank Open Knowledge Repository | French | ("maladie mentale" OR "trouble mental" OR "troubles mentaux" OR "trouble psychiatrique" OR "santé mentale" OR "suicide" OR "psychose" OR "psychologique" OR "psychiatrique") AND ("Polynésie française" OR "Vanuatu" OR "Wallis-et-Futuna" OR "Nouvelle-Calédonie") AND Filter: (Language: English, French), (Years: 2015-2025), (Region: East Asia and Pacific) |
| WHO IRIS | English | (Regional Office for the Western Pacific) AND [2015 TO 2025] AND English AND (“Mental Health” [MeSH] OR “Mental Disorders” [MeSH]) AND (“American Samoa” OR “Cook Islands” OR “Federated States of Micronesia” OR “Fiji” OR “French Polynesia” OR “Guam” OR “Kiribati” OR “Marshall Islands” OR “Nauru” OR “Niue” OR “Northern Mariana Islands” OR “Palau” OR “Samoa” OR “Solomon Islands” OR “Tokelau” OR “Tonga” OR “Tuvalu” OR “Vanuatu” OR “Pitcairn Islands” OR “Wallis and Futuna” OR “New Caledonia” OR “Papua New Guinea”) AND Filter: (year: 2015-2025) |
| WHO IRIS | French | (Regional Office for the Western Pacific) AND [2015 TO 2025] AND French AND (“santé mentale” [MeSH] OR “troubles mentaux” [MeSH]) AND ("Polynésie française" OR "Vanuatu" OR "Wallis-et-Futuna" OR "Nouvelle-Calédonie") AND Filter: (year: 2015-2025) |
| WHO Mental Health Atlas | English | Latest atlas reports after 2015-01-01 in PICs (“American Samoa” OR “Cook Islands” OR “Federated States of Micronesia” OR “Fiji” OR “French Polynesia” OR “Guam” OR “Kiribati” OR “Marshall Islands” OR “Nauru” OR “Niue” OR “Northern Mariana Islands” OR “Palau” OR “Samoa” OR “Solomon Islands” OR “Tokelau” OR “Tonga” OR “Tuvalu” OR “Vanuatu” OR “Pitcairn Islands” OR “Wallis and Futuna” OR “New Caledonia” OR “Papua New Guinea”) |
| UNICEF Pacific Islands | English | (“mental” OR “suicide” OR “psychosis” OR “psychological” OR “psychiatric”) after 2015-01-01 |
| UNICEF Pacific Islands | French | ("mental” OR “suicide” OR “psychose” OR psychologique” OR “psychiatrique") after 2015-01-01 |
| Pacific Community | English | (“mental ill” OR “mental illness” OR “mental disorders” OR “mental disease” OR “mental health” OR “suicide” OR “psychosis” OR “psychological” OR “psychiatric”) AND (2015-2025) |
| Pacific Community | French | ("maladie mentale" OR "trouble mental" OR "troubles mentaux" OR "trouble psychiatrique" OR "santé mentale" OR "suicide" OR "psychose" OR "psychologique" OR "psychiatrique") AND (2015-2025) |
| Pacific Island Health Officers Association | English | (“mental ill” OR “mental illness” OR “mental disorders” OR “mental disease” OR “mental health” OR “suicide” OR “psychosis” OR “psychological” OR “psychiatric”) AND (2015-2025) |
| Pacific Island Health Officers Association | French | ("maladie mentale" OR "trouble mental" OR "troubles mentaux" OR "trouble psychiatrique" OR "santé mentale" OR "suicide" OR "psychose" OR "psychologique" OR "psychiatrique") AND (2015-2025) |
| Asian Development Bank | English | (“mental ill” OR “mental illness” OR “mental disorders” OR “mental disease” OR “mental health” OR “suicide” OR “psychosis” OR “psychological” OR “psychiatric”) AND (2015-2025) |
| Asian Development Bank | French | ("maladie mentale" OR "trouble mental" OR "troubles mentaux" OR "trouble psychiatrique" OR "santé mentale" OR "suicide" OR "psychose" OR "psychologique" OR "psychiatrique") AND ("Polynésie française" OR "Vanuatu" OR "Wallis-et-Futuna" OR "Nouvelle-Calédonie") AND (2015-2025) |

**Supplementary Table 4** Data extraction form

| **Title** | **Last name of first author** | **Language** | **Study location** | **Study design** | **Leadership and governance** | | | | | **Financing** | | | | | **Health workforce** | | | | | |
| --- | --- | --- | --- | --- | --- | --- | --- | --- | --- | --- | --- | --- | --- | --- | --- | --- | --- | --- | --- | --- |
|  |  |  |  |  | **Policies, laws, legislation, regulations, strategies, plans, etc.** | **Stakeholders** | **Governance structure** | **Climate change or emergencies** | **Others** | **Total expenditure on mental health** | **Insurance coverage** | **Out-of-pocket payment** | **Climate change or emergencies** | **Others** | **Quantity and density** | **Distribution** | **Graduates of mental health professions** | **Health workforce capacity building** | **Climate change or emergencies** | **Others** |

| **Service delivery** | | | | | | | **Access to essential medicines** | | | | | **Health information systems** | | | | | |
| --- | --- | --- | --- | --- | --- | --- | --- | --- | --- | --- | --- | --- | --- | --- | --- | --- | --- |
| **Service availability: facilities** | **Service availability: inpatient beds** | **Service availability: outpatient department visits** | **Service readiness** | **Service quality** | **Climate change or emergencies** | **Others** | **Access to essential medicines (All content should be listed separately by country)** | **Affordability** | **Quality of use** | **Climate change or emergencies** | **Others** | **Data generation using core sources and methods** | **Monitoring and evaluation systems** | **Health reporting to general public** | **Country capacities for synthesis, analysis and validation of data** | **Climate change or emergencies** | **Others** |

**Supplementary Table 5** Included studies

| **Title** | **Last name of first author** | **Language** | **Study location** | **Study design** | **Leadership and governance** | **Financing** | **Health workforce** | **Service delivery** | **Access to essential medicines** | **Health information systems** |
| --- | --- | --- | --- | --- | --- | --- | --- | --- | --- | --- |
| Mental health in the Pacific: Urgency and opportunity | Ali 2020 | English | PICTs | Review and evidence synthesis | Yes |  | Yes | Yes |  | Yes |
| Integration of traditional and western treatment approaches in mental health care in Pacific Island Countries | Blignault 2020 | English | Cook Islands, Federated States of Micronesia, Fiji, Kiribati, Marshall Islands, Nauru, Niue, Palau, Papua New Guinea, Samoa, Solomon Islands, Tonga, Tuvalu, Vanuatu | Review and evidence synthesis | Yes |  | Yes | Yes |  |  |
| Capacity building for Pacific Island countries: the challenges and benefits of developing a postgraduate clinical training programme | Chang 2015 | English | Fiji | Report or position paper | Yes |  | Yes | Yes |  |  |
| Impact of a mobile-based (mHealth) tool to support community health nurses in early identification of depression and suicide risk in Pacific Island Countries | Chang 2021 | English | Fiji | Quasi-experiment study |  |  | Yes | Yes |  | Yes |
| Piloting online training in the Pacific-Ophelia project for child and adolescent mental health | Chang 2022 | English | Fiji, Marshall Islands, Papua New Guinea, Kiribati | Mixed Methods Study | Yes | Yes | Yes |  |  | Yes |
| The Rising Tide of Mental Disorders in the Pacific Region | Charlson 2015 | English | American Samoa, Cook Islands, Fiji, French Polynesia, Guam, Kiribati, Marshall Islands, Federated States of Micronesia, Nauru, New Caledonia, Niue, Northern Mariana Islands, Palau, Papua New Guinea, Pitcairn, Samoa, Solomon Islands, Tokelau, Tonga, Tuvalu, Vanuatu, Wallis and Futuna | Cohort study | Yes | Yes | Yes |  |  |  |
| Implementation of the mental health Gap Action Programme (mhGAP) within the Fijian Healthcare System: a mixed-methods evaluation | Charlson 2019 | English | Fiji | Mixed Methods Study | Yes | Yes | Yes | Yes | Yes | Yes |
| General and post-disaster mental health servicing in Vanuatu: A qualitative analysis | Dawes 2019 | English | Vanuatu | Qualitative study | Yes | Yes | Yes | Yes | Yes | Yes |
| The role of enculturation on the help-seeking attitudes among Filipino Americans in Guam | DeLuna 2020 | English | Guam | Cross-sectional study |  |  | Yes | Yes |  | Yes |
| Implementation of the Mental Health Gap Action Programme (mhGAP) in Kosrae State in the Federated States of Micronesia | Engelhard 2021 | English | Federated States of Micronesia | Qualitative study | Yes |  | Yes | Yes |  |  |
| Art making and the promotion of wellbeing in Samoa – participants’ lived experience of a recovery oriented intervention | Fenner 2018 | English | Samoa | Qualitative study | Yes |  | Yes | Yes |  | Yes |
| “I function when I’m painting” – Consumers, carers and staff experiences of an art and mental health recovery project in Fiji | Fenner 2022 | English | Fiji | Qualitative study | Yes | Yes | Yes | Yes |  | Yes |
| État des lieux des suicides et tentatives de suicide en Polynésie Française. Exemple de l’enquête “Santé Mentale en Population Générale: Images et Réalités”, réalisée à Tahiti et Moorea en 2015 | Fenni 2016 | French | French Polynesia | Mixed Methods Study | Yes |  |  | Yes |  | Yes |
| A qualitative evaluation of leadership development workshops for mental health workers from four Pacific Island Countries | Fung 2015 | English | Papua New Guinea, Federated States of Micronesia, Marshall Islands, Palau | Qualitative study | Yes |  | Yes | Yes |  | Yes |
| The Dementia Literacy Assessment (DeLA): A novel measure of Alzheimer's disease and related disorders health literacy in diverse populations | Galvin 2025 | English | American Samoa | Cross-sectional study |  | Yes | Yes | Yes |  | Yes |
| Child and adolescent psychiatric consultation in Micronesia | Ghiasuddin 2017 | English | Federated States of Micronesia | Qualitative study | Yes |  | Yes | Yes | Yes | Yes |
| Piloting a scalable, post-trauma psychosocial intervention in Tuvalu: The Skills for Life Adjustment and Resilience (SOLAR) program | Gibson 2021 | English | Tuvalu | Quasi-experiment study | Yes |  | Yes | Yes |  | Yes |
| Do clergy recognize and respond appropriately to the many themes in obsessive-compulsive disorder?: Data from a Pacific Island community | Gouniai 2022a | English | Guam | Cross-sectional study |  |  | Yes | Yes |  | Yes |
| Many common presentations of obsessive-compulsive disorder unrecognized by medical providers in a Pacific Island community | Gouniai 2022b | English | Guam | Cross-sectional study | Yes | Yes | Yes | Yes |  | Yes |
| Vanuatu tropical cyclones Judy and Kevin: post disaster needs assessment | Government of Vanuatu 2023 | English | Vanuatu | Report or position paper | Yes | Yes | Yes | Yes |  |  |
| Gender-based provisions in mental health legislation: a review of English language jurisdictions | Hoare 2025 | English | Fiji, Solomon Islands, Kiribati, Papua New Guinea | Review and evidence synthesis | Yes |  |  | Yes |  |  |
| Solomon Islands health system review | Hodge 2015 | English | Solomon Islands | Review and evidence synthesis | Yes | Yes | Yes | Yes | Yes | Yes |
| Development and usability of a mobile tool for identification of depression and suicide risk in Fiji | Iyengar 2021 | English | Fiji | Quasi-experiment study | Yes |  | Yes | Yes |  | Yes |
| Letting it be': a grounded theory about dementia care in Fiji | Johnston 2024 | English | Fĳi | Qualitative study | Yes |  | Yes | Yes |  | Yes |
| Building capacity for child and adolescent mental health and psychiatry in Papua New Guinea | Kowalenko 2020a | English | Papua New Guinea | Case study | Yes |  | Yes | Yes |  |  |
| Child and Adolescent Psychiatry International Relations (CAPIR): building bridges for psychiatry workforce capacity with Pacific Island nations | Kowalenko 2020b | English | Fiji, Samoa, Cook Islands, Tonga, Kiribati, Vanuatu, Solomon Islands, Papua New Guinea | Case study | Yes |  | Yes | Yes |  | Yes |
| Climate change, extreme events and mental health in the Pacific region | Leal 2022 | English | Tuvalu, Fiji, Samoa, Solomon Islands, Tonga, Federated States of Micronesia, Vanuatu | Mixed Methods Study | Yes |  |  | Yes |  | Yes |
| Social Change and Micronesian Suicide Mortality: A Test of Competing Hypotheses | Lowe 2019 | English | Federated States of Micronesia | Cross-sectional study | Yes |  |  |  |  | Yes |
| Suicide and suicide attempts in the Pacific Islands: A Systematic Literature Review | Mathieu 2021 | English | American Samoa, Cook Islands, Fiji, French Polynesia, Guam, Kiribati, Marshall Islands, Federated States of Micronesia, Nauru, New Caledonia, Niue, Northern Marianas, Palau, Papua New Guinea, Pitcairn Islands, Samoa, Solomon Islands, Tokelau, Tonga, Tuvalu, Vanuatu, Wallis and Futuna | Review and evidence synthesis | Yes |  | Yes | Yes |  | Yes |
| The mental health impact of climate change on Pacific Islanders: A systematic review focused on sea level rise and extreme weather events | Mengesha 2025 | English | Solomon Islands, Cook Islands, Tuvalu | Review and evidence synthesis | Yes |  | Yes | Yes |  | Yes |
| O le tagata ma lona aiga, o le tagata ma lona fa'asinomaga (Every person belongs to a family and every family belongs to a person): Development of a parenting framework for adolescent mental wellbeing in American Samoa | Mew 2024a | English | American Samoa | Qualitative study | Yes |  | Yes | Yes |  | Yes |
| "There are still broken or fragmented systems": Qualitative assessment of needs to strengthen adolescent mental health services in American Samoa | Mew 2024b | English | American Samoa | Qualitative study | Yes |  | Yes | Yes |  | Yes |
| 2017 Kiribati Annual Health Bulletin | Ministry of Health and Medical Services of Kiribati 2017 | English | Kiribati | Report or position paper | Yes | Yes | Yes | Yes | Yes | Yes |
| Suicide risk and prevention in Guam: Clinical and research considerations and a call to action (vol 83, 103546, 2023) | Monteith 2023 | English | Guam | Review and evidence synthesis | Yes | Yes | Yes | Yes |  | Yes |
| Rich Country, Poor People: the challenges of providing psychiatric services in the public and the private sectors in Papua New Guinea | Muga 2015 | English | Papua New Guinea | Qualitative study | Yes | Yes | Yes | Yes | Yes |  |
| Assessing the role of sustainability competencies in enhancing psychological first aid effectiveness for disaster responders in Fiji | Nair 2024 | English | Fiji | Cross-sectional study |  |  | Yes | Yes |  | Yes |
| Pervasive refusal syndrome in child asylum seekers on Nauru | Newman 2020 | English | Nauru | Case study | Yes |  | Yes | Yes | Yes | Yes |
| Content analysis of health-related subjects in the K12 school curricula of Japan, Indonesia, Philippines, Guam, Micronesia, Marshall Islands, Palau, and Fiji | Nishio 2023 | English | Federated States of Micronesia, Marshall Islands, Palau, Fiji, Guam | Cross-sectional study | Yes |  |  | Yes |  |  |
| Patterns of distress and psychosocial support 2 years post-displacement following a natural disaster in a lower middle income country | Nzayisenga 2022 | English | Vanuatu | Cross-sectional study | Yes |  | Yes | Yes |  |  |
| The Vanuatu Psychiatry Mentorship Programme: supporting the development of a fledgling mental health service in the Pacific | Obed 2020a | English | Vanuatu | Report or position paper | Yes | Yes | Yes | Yes | Yes | Yes |
| Vanuatu Psychiatry Mentorship Programme: a case illustrating cultural and clinical considerations | Obed 2020b | English | Vanuatu | Case study | Yes |  | Yes | Yes | Yes |  |
| Fijian adolescent emotional well-being and sexual and reproductive health-seeking behaviours | O'Connor 2019 | English | Fiji | Qualitative study | Yes |  | Yes | Yes |  | Yes |
| Mentoring: A Traditional Cook Island Approach to Support Men | Patterson 2023 | English | Cook Islands | Qualitative study | Yes |  | Yes | Yes |  |  |
| Anthropology, brokerage, and collaboration in the development of a Tongan public psychiatry: Local lessons for global mental health | Poltorak 2016 | English | Tonga | Qualitative study | Yes |  | Yes | Yes |  |  |
| CAMH in Primary Care Fiji: developing child and adolescent mental health in primary care | Robertson 2020 | English | Fiji | Report or position paper | Yes | Yes | Yes | Yes |  | Yes |
| Art and mental health in Samoa | Ryan 2015a | English | Samoa | Report or position paper | Yes |  | Yes | Yes |  |  |
| Mental health in the Solomon Islands: developing reforms and partnerships | Ryan 2015b | English | Solomon Islands | Report or position paper | Yes | Yes | Yes | Yes |  | Yes |
| Bridging the ocean: Kiribati Australia alliance in mental health | Ryan 2017 | English | Kiribati | Report or position paper | Yes |  | Yes | Yes |  |  |
| Art-making in mental health - A Fijian pilot study | Ryan 2021 | English | Fiji | Report or position paper | Yes |  | Yes | Yes |  | Yes |
| Mental health as a human right: challenges and opportunities in Fiji | Saxton 2018 | English | Fiji | Review and evidence synthesis | Yes | Yes | Yes | Yes |  | Yes |
| WHO Mental Health Gap Action Programme implementation in the Small Island Development States: experience from the Pacific and English-speaking Caribbean countries | Setoya 2018 | English | Niue, Tuvalu, Solomon Islands, Cook Islands, Samoa, Marshall Islands, Guam, Palau, Northern Marian Islands, Nauru, Vanuatu, Federated States of Micronesia, Fiji, Kiribati, Tokelau, Tonga | Review and evidence synthesis | Yes | Yes | Yes | Yes | Yes | Yes |
| Experience from mental health clinics held during medical service camps in Fiji | Sivakumaran 2015 | English | Fiji | Cross-sectional study | Yes |  | Yes | Yes | Yes |  |
| Asiasiga i A'oga ma Nu'u: a child and adolescent post-tsunami intervention based on Indigenous Samoan values | Tamasese 2020a | English | Samoa | Qualitative study | Yes |  | Yes | Yes |  | Yes |
| Asiasiga: a Samoan intervention to address the immediate mental health needs of Samoan communities after a tsunami | Tamasese 2020b | English | Samoa | Qualitative study | Yes |  | Yes | Yes | Yes | Yes |
| Lessons from COVID-19-free Vanuatu: intensive health operations for Phase 1 of repatriation and quarantine, May-July 2020 | Tapo 2021 | English | Vanuatu | Case study | Yes |  | Yes | Yes |  |  |
| The mental health and psychosocial impact of the Bougainville Crisis: A synthesis of available information | Tierney 2016 | English | Papua New Guinea | Review and evidence synthesis | Yes | Yes | Yes | Yes |  | Yes |
| Situation Analysis of Children in Fiji | UNICEF 2017a | English | Fiji | Report or position paper | Yes |  | Yes | Yes |  | Yes |
| Situation Analysis of Children in Kiribati | UNICEF 2017b | English | Kiribati | Report or position paper | Yes | Yes | Yes |  |  | Yes |
| Situation Analysis of Children in Nauru | UNICEF 2017c | English | Nauru | Report or position paper | Yes |  | Yes |  |  | Yes |
| Situation Analysis of Children in Niue | UNICEF 2017d | English | Niue | Report or position paper | Yes | Yes |  | Yes |  | Yes |
| Situation Analysis of Children in Palau | UNICEF 2017e | English | Palau | Report or position paper | Yes | Yes | Yes | Yes |  | Yes |
| Situation Analysis of Children in Samoa | UNICEF 2017f | English | Samoa | Report or position paper | Yes |  |  | Yes |  | Yes |
| Situation Analysis of Children in the Federated States of Micronesia | UNICEF 2017h | English | Federated States of Micronesia | Report or position paper | Yes | Yes | Yes | Yes |  | Yes |
| Situation Analysis of Children in the Marshall Islands | UNICEF 2017i | English | Marshall Islands | Report or position paper | Yes | Yes | Yes | Yes |  | Yes |
| Situation Analysis of Children in the Pacific Island Countries | UNICEF 2017j | English | Cook Islands, Federated States of Micronesia, Fiji, Kiribati, Marshall Islands, Nauru, Niue, Palau, Samoa, Solomon Islands, Tokelau, Tonga, Tuvalu and Vanuatu | Report or position paper |  |  | Yes | Yes |  | Yes |
| Situation Analysis of Children in the Solomon Islands | UNICEF 2017k | English | Solomon Islands | Report or position paper | Yes |  |  | Yes |  | Yes |
| Situation Analysis of Children in Tokelau | UNICEF 2017l | English | Tokelau | Report or position paper | Yes |  | Yes | Yes | Yes | Yes |
| Situation Analysis of Children in Tuvalu | UNICEF 2017n | English | Tuvalu | Report or position paper | Yes |  |  |  |  | Yes |
| Situation Analysis of Children in Vanuatu | UNICEF 2017o | English | Vanuatu | Report or position paper |  |  |  | Yes |  | Yes |
| Impact of COVID-19 on poor mental health in children and young people ‘tip of the iceberg’ – UNICEF | UNICEF 2021 | English | Fiji, Federated States of Micronesia, Vanuatu, Samoa, Solomon Islands | Report or position paper |  | Yes | Yes | Yes | Yes | Yes |
| The effectiveness of uloa as a model supporting Tongan people experiencing mental distress | Vaka 2022 | English | Tonga | Qualitative study |  |  | Yes | Yes | Yes | Yes |
| Human Health and Climate Change in Pacific Island Countries | WHO 2015a | English | American Samoa, Cook Islands, Federated States of Micronesia, Fiji, French Polynesia, Guam, Kiribati, Marshall Islands, Nauru, Niue, Palau, Papua New Guinea, Samoa, Solomon Islands, Tokelau, Tonga, Tuvalu, Vanuatu, New Caledonia | Report or position paper | Yes |  | Yes | Yes | Yes | Yes |
| WHO profile on mental health in development (WHO proMIND): Cook Islands | WHO 2015c | English | Cook Islands | Report or position paper | Yes | Yes | Yes | Yes |  | Yes |
| WHO proMIND: profiles on mental health in development: Republic of the Marshall Islands | WHO 2015d | English | Marshall Islands | Report or position paper | Yes | Yes | Yes | Yes | Yes | Yes |
| Independent State of Papua New Guinea Health System Review | WHO 2019a | English | Papua New Guinea | Review and evidence synthesis | Yes | Yes | Yes | Yes |  | Yes |
| Live life: an implementation guide for suicide prevention in countries | WHO 2021a | English | Fiji, Papua New Guinea | Report or position paper | Yes | Yes | Yes | Yes |  | Yes |
| Mental Health Atlas Cook Islands 2020 | WHO 2021b | English | Cook Islands | Report or position paper | Yes | Yes | Yes | Yes |  | Yes |
| Mental Health Atlas Fiji 2020 | WHO 2021c | English | Fiji | Report or position paper | Yes | Yes | Yes | Yes |  | Yes |
| Mental Health Atlas Kiribati 2020 | WHO 2021d | English | Kiribati | Report or position paper | Yes | Yes | Yes | Yes |  | Yes |
| Mental Health Atlas Marshall Islands 2020 | WHO 2021e | English | Marshall Islands | Report or position paper | Yes | Yes | Yes | Yes |  | Yes |
| Mental Health Atlas Micronesia (Federated States of ) 2020 | WHO 2021f | English | Federated States of Micronesia | Report or position paper | Yes | Yes | Yes | Yes |  | Yes |
| Mental Health Atlas Niue 2020 | WHO 2021g | English | Niue | Report or position paper | Yes | Yes | Yes |  |  | Yes |
| Mental Health Atlas Palau 2020 | WHO 2021h | English | Palau | Report or position paper | Yes | Yes | Yes | Yes |  | Yes |
| Mental Health Atlas Papua New Guinea 2020 | WHO 2021i | English | Papua New Guinea | Report or position paper | Yes | Yes | Yes | Yes |  | Yes |
| Mental Health Atlas Solomon Islands 2020 | WHO 2021j | English | Solomon Islands | Report or position paper | Yes | Yes | Yes | Yes |  | Yes |
| Mental Health Atlas Tonga 2020 | WHO 2021k | English | Tonga | Report or position paper | Yes | Yes | Yes | Yes |  | Yes |
| Mental Health Atlas Tuvalu 2020 | WHO 2021l | English | Tuvalu | Report or position paper | Yes | Yes | Yes | Yes |  | Yes |
| Mental Health Atlas Vanuatu 2020 | WHO 2021m | English | Vanuatu | Report or position paper | Yes | Yes | Yes | Yes |  |  |
| Regional framework for the future of mental health in the Western Pacific 2023-2030 | WHO 2023 | English | Fiji, Papua New Guinea, Samoa, Tonga | Report or position paper | Yes | Yes | Yes | Yes |  |  |
| Psychosocial support during displacement due to a natural disaster: relationships with distress in a lower-middle income country | Zahlawi 2019 | English | Vanuatu | Cross-sectional study | Yes | Yes | Yes | Yes |  |  |
| Psychoéducation familiale: connaissances et attentes des aidants familiaux des patients souffrant de schizophrénie en Polynésie française | Zumbiehl 2020 | French | French Polynesia | Mixed Methods Study |  | Yes |  | Yes | Yes | Yes |

**Supplementary Table 6** Mental health system performance across the six building blocks by Pacific Island countries and territories (PICTs)

| **PICT** | **Leadership and governance** | **Financing** | **Health workforce** | **Service delivery** | **Access to essential medicines** | **Health information systems** |
| --- | --- | --- | --- | --- | --- | --- |
| American Samoa | • Relies mainly on US law, no isolated information available  • Leadership structure: Department of Health, American Samoa Alliance Against Domestic & Sexual Violence, Empowering Pacific Island Communities (EPIC)^1^ • Governance emphasizes community participation (healthcare professionals, religious leaders, teachers, social workers)^1,2^ | • Significant gaps in resourcing and treatment coverage^3^ • No isolated information is available on funding details | • Only 4 doctoral-level clinical psychologists/psychiatrists; 20 mental health counselors/clinicians with varying training/licensing levels^4^ • Geographical isolation hinders professional support access and ongoing development^3^ | • Community-based services new; insufficient for adolescent needs^4^ • Difficult to quantify program effectiveness^4^ • Family-based interventions feasible^1^ • Cultural adaptation examples: Dementia Literacy Assessment (DeLA) translation^2^ • Community health education and psychosocial support strengthened through various initiatives^5^ • Expanded radio helpline campaigns (“you are not alone”)^4^ • Alzheimer's Disease and Related Dementias (ADRD) literacy improvement needed^2^ | • No information available | • No information available |
| Cook Islands | • Centralised governance by the Ministry of Health (Mental Health); no dedicated mental health authority^6,7^ • Draft mental health policy (2009) not adopted; under review to include WHO Action Plan 2013-2020^6^ • Stand-alone mental health policy/plan since 2020; mental health law since 2013^7^ • National suicide prevention program "Surviving the storm"^7^ • "mental health & Wellbeing Strategy 2016-2020" prioritizes disaster response capacity^8,9^ • Regional collaboration expanded since 2013 (professionals, NGOs, universities, multilateral agencies)^10^ • Royal Australian and New Zealand College of Psychiatrists (RANZCP) established Child and Adolescent Psychiatry International Relations (CAPIR) Subcommittee (2017) supports strategic planning^10^ | • Tax-based system (British model); government pays medical fees and medicines^5^  • Mental health budget integrated into overall health budget^6^ • Integrated budget funds: 1 senior position + 1 community nurse (Ministry); 1 psychiatric nurse (Te Kainga NGO)^6^ • Mental health expenditure: 1.0% of total health budget^7^ | • 1 medical officer (psychiatrist capacity) at Rarotonga Hospital; 1 psychiatric nurse at Te Kainga Clinic^6^ • No specialist-trained mental health personnel at other facilities across 11 inhabited islands^6^ • Per 100k: 0 psychiatrists, 22.8 mental health nurses, 5.7 psychologists, 5.7 social workers, 22.8 other specialists; Total: 56.99^7^ • Royal Australian and New Zealand College of Psychiatrists Capacity-building: leadership training, peer collaboration, culturally sensitive workforce development^10^ • Initiated mental health Gap Action Programme (mhGAP) training^5^  • WHO  proMIND :Training at or via distance learning (PEACESAT); Staff attend conferences/seminars, workshops/training^11^  • UNICEF supporting capacity strengthening for frontline workers^12^ | • No specialised mental health facilities; acute cases treated in primary care; violent patients in Aorangi Prison^6^ • Secondary care in private rooms at Rarotonga Hospital; primary care lacks privacy/security^6^ • 11.4 community-based mental health facilities per 100k^7^ • Traditional treatments: medicine, spells, rituals, counseling^13^ • Male mentoring program: 24-hour community service for domestic violence/mental health issues; prevents reoffending^14^ • Creating Futures Conferences & Leadership courses promote child/youth focus, primary care integration^10^ • Volunteer mentoring in Vanuatu is well-regarded^10^ • WHO/START study supports suicide surveillance, awareness campaigns, crisis helplines^15^ | • Services/medicines free at point of use (fully insured)^7^  • mhGAP ensures drug availability at target facilities^5^ • Supply chain to remote islands remains a barrier  • Need consistent psychiatric drug supply (e.g., antipsychotics) to outer islands^8^ | • Weak health information systems hinder monitoring/evaluation; need integration & core mental health indicator reporting^5^  • Monitoring or evaluation systems not well established^6^  • WHO/START study establishes suicide/self-harm surveillance infrastructure^15^ |
| Federated States of Micronesia | • Governance emphasises extended family & matrilineal clan groups; Yap maintains hierarchical traditional leadership^16^ • All cultures have formal/informal power bases for decision-making^17^ • Stand-alone mental health law since 1989^18^; This law is outdated/inadequate; allows detention of mentally ill in jail/penal institutions for "insanity"^19^ • As of 2014: no national mental health policy; National mental health Policy, Strategy & Action Plan underway^19^ • By 2015: stand-alone mental health policy/plan established; human/financial resources estimated & allocated^18^ • The mhGAP training (Kosrae) via collaboration: Kosrae Community Health Center + University of Hawaii Dept. of Psychiatry^20^ • Mental health is included in Sustainable Development Goals^20^ • WHO mhGAP highlights productive partnerships^17^ • Mental health education: FSM is the only country without mental health-related curriculum (other countries teach via morals/religion or health)^21^ | • Tax-based system (British model); government pays medical fees + medicines^9^ • Health insurance does not cover mental health/substance abuse treatment; no government funding is allocated specifically to mental health/substance abuse^19^ • Relies heavily on external development aid & US support via Compact of Free Association^19^ • Majority pay nothing at point of service for mental health services/psychotropic medicines (fully insured)^18^ • Too little government investment; wide gaps between mental health needs & funding^12^ | • 1 psychiatrist for the entire country (based in Chuuk); serves all 4 states^19^ • No child psychiatrists on Yap^16^ • Per 100k: 0.88 psychiatrists, 3.51 mental health nurses, 0 psychologists, 4.39 social workers, 0 other specialists; Total: 8.79^18^ • Workforce includes traditional healers & psychiatrists^13^ • Limited access: small remote islands, limited workforce^20^ • Lacks appropriate equipment & adequately trained professionals (health sectors)^19^ • mhGAP trained doctors/nurses^9,20^ • Workshops 1-2 times/year: clinical skills (assessment/management) & train-the-trainer programs^17^ • WHO proMIND: Training at or via distance learning (PEACESAT); Staff attend conferences/seminars, workshops/training^11^  • UNICEF supporting capacity strengthening for frontline workers^12^ | • As of 2014: no inpatient mental health facilities; only holding units in each of the 4 state hospitals^19^  • 4 mental health outpatient facilities attached to hospital^18^ • Mental health care largely community-based & integrated into primary care^19^ • Severely limited access: small scattered islands; mental health treatment gap >90%^20^ • Extreme weather events decrease health service access during disasters^22^ • Traditional treatments: medicine, spells, rituals, counseling^13^ • Traditional healers & mental health professionals autonomous but cooperative; fully integrated hybrid model^13^ • Educational approaches via school curricula^21^ • Psychological first aid after Tropical Cyclone Winston^22^ | • Most pay nothing at points of service for psychotropic medicines (government system)^18^ • Limited pharmaceutical options, especially specialized psychotropic medications • Clonidine is the only medication available for ADHD on Yap^16^ | • Significantly underdeveloped; major barriers to monitoring/evaluation/evidence-based planning^9^ • Health information system in most PICTs cannot precisely quantify mental disorder prevalence (general/adolescent population)^19^ |
| Fiji | • Healthcare system: 4 divisions (Central, Western, Northern, Eastern); Ministry of Health & Medical Services (MOHMS) oversees services^23^ • Centralised governance; MOHMS primary authority^24^  • MOHMS seeking mental health service enhancement via decentralization (WHO & district health manager support)^25^ • Mental Health Act (2010)/Mental Health Decree: directs deinstitutionalization & community services; modern human rights framework; promotes community treatment & least restrictive care in cultural context^23,26^ • Stand-alone mental health policy/plan since 2015; stand-alone mental health law since 2010^27^ • Strategic mental health & Suicide Prevention Plan 2007-2011; National mental health Strategic Plan 2012-2016^24^ • National Strategic Health Plan 2016-2020: localized, recovery-oriented approach; lacks clear implementation guidance^28^ • Legislation: female representation on mental health tribunals/boards/authorities; protections during restraint/search; separate facilities for men/women; protection for mothers receiving care^29^ • Committed to WHO mhGAP as backbone of national mental health program^23^ • Key stakeholders: Fiji MOHMS, WHO, Queensland Centre for Mental Health Research, FNU School of Medical Sciences^23,28^ • Collaboration with NGO Fiji Alliance for Mental Health for post-Cyclone Winston relief (2016) ^14^ • Partners: Fiji Alliance for Mental Health, Suva College Medicine/Nursing/Health Sciences, FNU^28^ • Child/adolescent mental health identified as public health priority^30^ • International frameworks: Convention on the Rights of Persons with Disabilities, WHO Comprehensive mental health Action Plan 2013-2030, Quality Rights Initiative reforming mental health law^29^ | • Tax-based system (British model); government pays medical fees + medicines^9^ • Majority pay nothing at point of service for mental health services/psychotropic medicines (fully insured)^27^ • Government mental health expenditure: 1% of total health budget^27^ • 96.8% of government mental health expenditure allocated to mental hospitals^27^ • COVID-19 imposed further limitations on over-stretched health service in Suva^28^ • Community programs centrally-based in Suva; heavily reliant on volunteer staff; no secured ongoing funding^26^ | • Few psychologists, social workers, occupational therapists^31^ • Per 100k: 0.56 psychiatrists, 5.17 mental health nurses, 0 psychologists, 0.34 other specialists; Total: 6.07^27^ • Current: 4 psychiatrists, 3 psychiatric registrars (postgraduate diploma), 50 nurses (postgraduate certificate mental health nursing), >200 nurses (WHO mhGAP training)^30^ • Child/adolescent psychiatrists: 0.30 per 100k; total child/adolescent mental health workers: 0.30 per 100k^27^  • Community health clinics throughout islands staffed by physicians/community health nurses^32^ • Small psychiatric workforce; lack of formal training pathway delays development^31^ • Main barrier: time pressures on health facility staff^23^ • Lack of medical specialists for abuse/mental health issues; no permanent psychiatrist^24^ • Dementia care: service providers need more education/training^33^ • Fiji National University developed 1-year full-time Postgraduate Diploma in Mental Health program for doctors, nurses, allied mental health workers to address suicide/substance abuse^30^ • 10-week pilot training for non-specialists/frontline staff: disaster mental health, violence/aggression, suicide management, developmental disorders, mentoring, child abuse, intellectual disability, family violence, clinician self-care • Child and Adolescent Mental Health in Primary Care Fiji (Lautoka): graduate doctors, nurses from divisional/subdivisional hospitals, medical centres, nursing stations; aims to develop self-sustained training expertise^31^ • St. Giles Hospital: regular art sessions by local art makers/mental health professionals for CROP/St. Giles patients^34^ • International medical service camps supplement workforce: doctors, dentists, ophthalmologists, gynecologists, pediatricians, GPs, nurses, pharmacists, clinical psychologist^35^ • UNICEF supporting capacity strengthening for frontline workers (identify/support/refer distressed children from COVID-19/violence/abuse/neglect/exploitation)^12^ • Disaster response: psychological first aid training for responders^36^  • WHO proMIND: Training at or via distance learning (PEACESAT); Staff attend conferences/seminars, workshops/training^11^ | • Services mainly hospital-based^23^ • 136 beds at National Psychiatric Hospital (St Giles Hospital, Suva): treats acute/chronic mental illness, intellectual disability, forensic cases^35^ • St Giles: colonial era psychiatric hospital; houses much of country's mental health capacity^31^ • Stress Management Wards in divisional hospitals: Eastern (Colonial War Memorial, Suva), Western (Lautoka), Northern (Labasa); no separate child/adolescent services^31^  • Formal dementia care via public mental health services: special outpatient clinics at health centres, sub-divisional/divisional hospitals, St Giles^33^ • Poor communication networks between mental health services (St. Giles, public health, mental health units, community health workers); challenges in referral pathways/information flow^23^ • 0.11 community-based mental health facilities per 100k^27^ • Per 100k: 10.79 mental hospital beds; 62.03 annual admissions^27^ • <10% of population requiring mental health care can readily access appropriate support^26^ • Lack of specialist services in rural areas^35^ • Absence of specialized services for older people, especially those with dementia & families^33^ • Adolescent services: counselling for sexual abuse/pregnancy (Medical Services Pacific, Empower Pacific, Youth Champs 4 Mental Health); national youth hotline^37^ • Innovative initiatives: occupational health program at St. Giles; Community Recovery Outreach Program (CROP, Suva)^34^ • Culturally adapted interventions: weekly art-making sessions^34^, public exhibition^28^ • Technology: mHealth tool for depression/suicide risk assessment by community health nurses^32,38^ • School-based mental health education^21^ • International medical service camps^35^ • Prevention/intervention programs: SADS (suicide prevention, National); Early Child Development Wellness Program (Maternal Child Health, National); School Health Team (Regional); Mental Health in Workplace (National); PFA through FEMAT (Fiji Emergency Medical Assistance Team, National)^27^ | • Psychotropic medicines included in national health insurance/reimbursement schemes^27^ • Policy provides financial protection but doesn't guarantee availability/timely access^27^ | • Very limited epidemiological research/data on mental health prevalence^35^  • Secure technology access & appropriate telehealth facilities not yet routinely available in mental health services^39^ • mHealth tools developed: depression identification^38^， suicide risk/depression assessment^32^  • Prospective injury surveillance systems in hospitals enable complete analyses of fatal/non-fatal intentional self-harm^40^ |
| French Polynesia | • Centralised governance structure^41^ • SOS Suicide association partnership with French Polynesia Hospital Center established^41^ • Suicide prevention center established 2012 in partnership with municipality of Punaauia^41^ • Stakeholders: SOS Suicide association, French Polynesia Hospital Center, patients/families, general practitioners/specialists, media, traditional healers/Taurumi practitioners, researchers/evaluators^41^  • Ministry plans: specialized rehabilitation teams, therapeutic education programs for patients^12,42^ | • Ministry plans: financial support to community organizations (e.g., Taputea Ora), direct financial assistance to caregivers^42,43^ • Primary cost: clinical staffing (medical/paramedical professionals); Secondary costs: administrative support, specialist facilitators, meeting spaces, educational materials^42,43^ • Family psychoeducation programs: financial breakeven within 1-2 years through reduced hospitalization^42,43^ | • Healthcare staff currently under significant strain from chronic patient overcrowding ^12,42^ | • Prevention measures: media campaigns, removing healthcare access barriers, improving treatment for mental health/substance abuse^41^  • Services: crisis hotline, mobile intervention, psychoeducation, voluntary listening sessions, body-mediated therapies, educational programs for patients/carers, follow-up calls by psychologists, communication technologies (SMS, internet, social media, telemedicine)^41^ • Structured family psychoeducation not currently part of standard schizophrenia care^12^ | • No information. | • "Mental Health in the General Population: Images and Realities" survey conducted 2015 with French WHO Collaborating Center ^41^. Data provides opportunity to estimate prevalence of mental disorders in general population aged 18+, especially suicide risk^41^ |
| Wallis & Futuna | • No information | • No information | • Very few MH specialists (psychiatrists, psychologists) in the Pacific^3^ | • No information | • No information | • No information |
| Guam | • US territory; governance within US federal system while addressing Pacific-specific mental health needs^15^ • WHO mhGAP framework applied regionally^5^ • Educational policy shows hybrid cultural–scientific approach; Guam in intermediate state^21^  • Healthcare access recognized as governance priority^15^ | • Operates within US healthcare system; faces Pacific-region resource constraints^15^ | • Few specialists (psychiatrists, psychologists) regionally^2^  • Only 35.8% of mental health needs met due to workforce shortages^42^ • Reliance on non-specialist medical providers^42^ • Clergy often used for mental health help-seeking, especially Filipino American & Pacific Islander groups^42,44^ • Clergy lack evidence-based training^42^ • Need for training on OCD recognition for nonpsychiatric providers^42^ | • Only 35.8% of mental health needs met^42^ • Cultural factors strongly shape help-seeking; informal religious systems function as de facto mental health services^44^ ^45^  • United States Department of Veterans Affairs (VA) community-based outpatient clinic provides MH services; nearest VA medical center in Hawaii^15^ | • WHO mhGAP framework includes ensuring drug availability^5^ | • No information |
| Kiribati | • Centralised system^46^; outdated Mental Health Act 1977 (under review; not CRPD-aligned)^47^  • No dedicated authority body^48^  • Stand-alone mental health policy/plan since 2016; stand-alone mental health law since 2020^48^  • No human resources or financial resources allocated for mental health plan^48^  • Governance structure: Ministry of Health and Medical Services operates at Central/District/Island/Community levels; 22 island councils^49^  • Strategic plans lack mental health indicators/targets^47^  • Child/adolescent mental health identified as priority^26^  • Climate pressures exacerbate health system burdens^49^ | • Free healthcare; minimal OOP spending^49^  • Publicly financed, government is main provider^49^ • 2017 health spending = 14.2% of recurrent budget; per capita AUD $282^49^  • No separate mental health budget; funding inadequate^46^  • Government mental health expenditure = 5% of health spending^48^  • 100% of mental health budget spent on mental hospitals^48^  • Mental health conditions covered under national insurance; fully insured at point of care^48^ | • Mental health workforce per 100k: Psychiatrists 0.85; mental health nurses 14.45; Psychologists 0.85; Social workers 0.85; Other mental health workers 18.71; Total 35.71^48^  • Workforce includes community workers, counsellors, pediatric doctors, nurses^26^  • Few mental health specialists in region^2^; minimal mental health training^46^  • Tungaru Central Hospital: medical specialists + Intern Medical Officers; mental ward staffing: 2 Medical Officers, 1 assistant, 12 nurses^49^; includes consultant psychiatrist, registrars, orderlies^47^  • Training initiatives (Leadership in Mental Health: Island Nations): 10-week pilot (disaster mental health, suicide, CAMH, violence, self-care etc.)^26^; 4-week governance/program-quality training (OPHELIA training program)^47^  • No formal mental health courses; rely on international partnerships (Fiji, Korea, Australia)^47^  Kiribati has implemented mhGAP and trained doctors/nurses^5^  • UNICEF supporting capacity strengthening for frontline workers^12^ | Te Meeria Ward is the sole mental health service^47^  • Mental hospital beds/admissions per 100k: 38.26 / 204.07^48^ • Outpatient visits per 100k: 1312.84^48^ | • Only a few registered pharmaceutical shops^49^  • Ensuring medicine availability a key mhGAP component^5^ | • Data collection difficult due to geography and limited internet^46^  • Health information system cannot quantify mental health prevalence; adolescent mental health a concern^46^  • Annual “Kiribati Health Bulletin” summarises ministry of health and medical services achievements^49^ |
| Marshall Islands | • Centralised mental health system^50^ • Mental Health Policy adopted 2011: mission to provide comprehensive, affordable, contextual, culturally acceptable services; access without discrimination^11^ • Stand-alone policy/plan for mental health since 2020^51^ • New mental health law being drafted to comply with Convention on the Rights of Persons with Disabilities^50^ • Public Health, Safety and Welfare Act 1966 (revised 2012): outdated, doesn't protect rights, uses stigmatizing language ("insane")^11^ • Public Health Administration oversees mental health governance^11^ • Maternal and Child Health department + Division of Human Services conduct community needs assessments for suicide prevention^11^ • Human resources estimated and allocated for policy/plan implementation^51^ • Stand-alone/integrated strategy for suicide prevention^51^ • National programmes: School & Community Outreach, Mental Health Awareness/Anti-stigma, Early Child Development Mental Health/MCH, School Outreach, Parental/Maternal mental health, Trainings and Workshops, Psychological and Psychosocial Rehabilitation for disaster preparedness^51^ | • Marshall Islands spends ~0.4% of total health budget (US$22.6 million in 2012) on mental health (2012)^50^ • US funds 80% of country's overall annual budget^11^  • Government total expenditure on mental health: 8% of total government health expenditure (2020)^51^ • Government expenditure on mental hospitals: 13.9% of total government mental health expenditure (2020)^51^ • Additional funding sources: Asian Development Bank, International Monetary Fund, World Bank, European Union, Japan, Taiwan, China governments for MDGs^11^ • Health insurance premiums from workers' salaries: $2.9-$4.3 million in recent years^11^ • Funding geared towards medications and staff, not psychosocial interventions^11^ • Care/treatment of mental health conditions (psychosis, bipolar, depression) not included in national health insurance/reimbursement schemes^51^ • Majority of persons with mental health conditions pay nothing at point of service for both services and medicines (fully insured)^51^ | • Per 100k: 1.70 psychiatrists, 5.10 mental health nurses, 0 psychologists, 0 social workers, 6.80 other specialized workers (e.g., occupational therapists); Total: 13.61^51^ • Workforce: traditional healers, psychiatrists [13], community workers, counselors, doctors (mental health, pediatrics), nurses^39^ • Health assistants with very limited mental health knowledge provide primary services in outer islands^11^ • Lack of appropriately trained mental health professionals, especially on Outer Islands^50^ • WHO  proMIND :Training at College of Marshall Islands or via distance learning (PEACESAT); Staff attend conferences/seminars, workshops/training^11^  • Nurses Registration Board endorses continuing professional education^11^ • No mental health trainings available in-country for mental health/general health professionals^11^ • 10-week pilot training for non-specialists/frontline staff^39^  Marshall islands has implemented mhGAP and trained doctors/nurses^5^  • UNICEF supporting capacity strengthening for frontline workers^12^  • Leadership Development Workshops 1-2 times yearly: clinical skills, train-the-trainer programmes^17^ | • General hospital psychiatric unit beds: 17.01 per 100k; annual admissions: 23.81 per 100k^51^ • Two hospitals provide inpatient care for mental health conditions but no specific designated beds^11^ • No specialist facilities/hospitals for mental health^11^  • 2 mental health outpatient facilities attached to hospital; 2 psychiatric units in general hospitals^51^ • Specialist care referrals: hospitals in Hawaii or Philippines^11^ • Home visits available for patients unable to travel; support for family members^11^ • Psychotropic medication assessment: doctor or advanced practice nurse under supervision; only psychiatrist/physician can prescribe; nurse under psychiatrist supervision can dispense^11^  • Health education/promotion campaigns in schools, community groups, churches, youth groups^11^ | • Some WHO-recommended psychotropic medications available^11^ • Not available: Fluoxetine, Chlorpromazine, Clomipromine, Sodium Valproate, methadone^11^ • Supply of psychotropic medicines sporadic, partly due to funding limitations; results in supply interruptions^11^ • When available, medicines supplied to Health Assistants in outer atolls for client provision^11^ • Drug availability at target facilities is mhGAP implementation priority^9^ | • Marshall Islands compiles mental health data in quarterly/annual reports of mental health program^11^ • As US territory in free association, US SAMHSA reports mental health outcome data in yearly reports (limited data)^11^ • Marshall Islands has not implemented Global School-based Student Health Survey (GSHS)^50^ |
| Nauru | • Centralised mental health system^52^ • Mental Health Policy established 2007^13^ • Alliance formed (Australian Medical Association, doctors, lawyers, refugee advocates, Church groups) to change Migration Act, depoliticize healthcare/treatment decisions for Pervasive Refusal Syndrome in child asylum seekers on Nauru^53^ | • 2012 situation analysis: no separate budget allocation for mental health, no medical staff dedicated to mental health^52^ | • Limited mental health services available only at one hospital (Republic of Nauru Hospital): 1 doctor, 1 locally trained nurse assistant with some mental health experience^52^ • Psychiatrist from Tonga visits ~3 times per year^52^ • Nauru has initiated mhGAP and trained doctors and nurses [10]  • WHO proMIND: Training at or via distance learning (PEACESAT); Staff attend conferences/seminars, workshops/training^11^  • UNICEF supporting capacity strengthening for frontline workers^12^ | • Provision of standard care difficult due to limited diagnostic, medical and psychiatric treatment options on Nauru^53^  • Nauru's capacity to provide adequate mental health services heavily reliant on expatriates and overseas assistance^52^  • Provision of effective psychiatric treatment limited by absence of specialist medical services for traumatized children on Nauru^53^ | • Drug availability at target facilities is mhGAP implementation priority^9^ | • Health information system does not allow precise quantification of mental disorder prevalence in general population or among adolescents^52^ • Lack of data highlighted in 2012 situation analysis of mental health in Nauru^52^ |
| New Caledonia | May be involved in some WHO pacific islands mental health programmes, no isolate information was found. | | | | | |
| Niue | • National Youth Policy (2009-2013): youth leadership and mental health promotion^54^ | • Mental health services and psychotropic medicines: fully covered, persons pay nothing^55^ • Treatment for psychosis, bipolar, depression included in national insurance^55^ • Mental health spending: 1% of total government health expenditure; Mental hospitals: 100% of mental health expenditure^55^ • New hospital funded by WHO, EU, New Zealand; site chosen inland to reduce cyclone risk^54^ | • Per 100k: 123.92 mental health nurses; Total: 123.92 professionals^55^ • Outpatient visits last year: 1,177.20^55^ • Lack sufficient population to sustain specialists; professionals work in isolation with limited support/development opportunities^39^  • WHO proMIND: Training at or via distance learning (PEACESAT); Staff attend conferences/seminars, workshops/training^11^  • UNICEF supporting capacity strengthening for frontline workers^12^ | • Mainly hospital-based^13^ | • Medicines fully covered by government, persons pay nothing^55^ • mhGAP objective: ensure availability of essential psychotropic medicines at target facilities^9^ | • No specific information system available^55^ |
| Northern Mariana Islands | May be involved in some WHO pacific islands mental health programmes (WHO proMIND, mhGAP can be identified), no isolate information was found. | | | | | |
| Palau | • Centralised governance structure^56^ • National Youth Policy: youth opportunities in national development^56^ • No officially approved mental health policy; mental health not mentioned in general health policy^56^ • Stand-alone policy/plan for mental health exists; dedicated authority body exists^57^ • Authority to assess compliance with international human rights exists but not functioning well^57^ • Government programmes: Project Kerreu (Mental Health Awareness/Anti-stigma), School-based prevention/promotion, Primary Care Integration (Parental/Maternal mental health), Work-related prevention/promotion, Critical Incident Stress Management (disaster preparedness)^57^ | • Mental health expenditure: 0.82% of total health budget as of 2011^56^ • Total mental health expenditure per person: 16.7 USD^57^ • Care/treatment for psychosis, bipolar, depression included in national health insurance/reimbursement schemes^57^ | • Per 100k: 11.11 psychiatrists, 50.00 mental health nurses, 0 psychologists, 11.11 social workers, 55.55 other specialists (e.g., occupational therapists); Total: 127.77^57^ • Most primary healthcare physicians/nurses have not received mental health training; official referral procedures not in place^56^ • Leadership Development Workshops 1-2 times yearly: clinical skills, train-the-trainer programmes^17^  • Hygiene-related curriculum: one page only, very limited^21^  • Palau has initiated mhGAP and trained doctors and nurses  • WHO  proMIND :Training at or via distance learning (PEACESAT); Staff attend conferences/seminars, workshops/training^11^  • UNICEF supporting capacity strengthening for frontline workers^12^ | • 80-bed Belau National Hospital in Koror; very centralised, increases health system vulnerability^56^ • 4 primary care super dispensaries, 4 community-based dispensaries on Outer Islands; 3 private primary healthcare clinics^56^ • Mental health service capacity at peripheral facilities limited^56^ • Facilities: 1 mental health outpatient facility attached to hospital, 1 psychiatric unit in general hospital, 1 other outpatient facility^57^ • Visits per 100k last year: Community-based 183.32; Other outpatient 1,388.81; Children/adolescents 15,277.78; Hospital-attached 1,738.79^57^ • Major challenge: high cost and administrative difficulty delivering services to population dispersed across remote islands with minimal infrastructure/transport^56^ | • Majority pay at least 20% towards cost of psychotropic medicines^57^ • Creates potential financial barriers to medication adherence, especially for long-term treatment^57^ • mhGAP includes ensuring drug availability at target facilities^9^ • High cost/administrative difficulty delivering services to dispersed islands likely extends to pharmaceutical supply chains^56^ | • Very little quantitative data on mental health; difficult to establish prevalence, incidence, profile of mental health problems^56^ • GSHS not yet implemented; would contain suicide attempt information^56^ • WHO planning comprehensive mapping of Palau's mental health system (WHO proMIND) to address data gaps^56^ |
| Papua New Guinea | • National Mental Health Policy 2010^13^; stand-alone law 2016^58^; lacks overarching integrated plan^59^ • National Health Services Standards: psychiatry/mental health/addiction as 1 of 5 core areas; each of 20 Provincial Hospitals should have 1 psychiatrist^60,61^ • National Mental Health Programme established 1962; no strategies outlined^61^ • Faculty of Community and Allied Health Professions established Community and Allied Health Professions Institutional Review Subcommittee 2017 ^60^ | • "Budgetary Cinderella service," low priority ^62^ • 1.0% of total health expenditure; 98% to mental hospitals ^58^ • Tiny minority have insurance; many insurers exclude mental illness or only cover initial episode^62^ • Majority pay nothing (fully insured through public system)^58^ • Large treatment gap; high stigma, discrimination, human rights violations^61^ • Services deteriorated due to insufficient finance/HR at district level^61^; insecure/intermittent funding^59^ | • Per 100k: 0.14 psychiatrists, 1.16 mental health nurses, 0.05 psychologists, 0.03 social workers, 0 other; Total: 1.38 ^58^ • 9 total psychiatrists (7 clinical) serving >7 million; 11 for 8 million^60,62^ • Maldistribution: 5 Port Moresby, 2 Lae; 6.76 million in 20 provinces without psychiatrist^62^ • Masters in Psychiatry at University of Papua New Guinea: only 13 graduated since 1990s; insufficient new graduates^60,61^ • Average 10 mental health nurses qualify yearly; absorbed into general disciplines^61^  • 4-day Centre for Addiction and Mental Health training at University of Papua New Guinea^60^ | • Only 1 psychiatric hospital (Laloki, 1967, 80 beds) outside capital; few provincial units; most offer only outpatient care by psychiatric nurses ^61,62^ • Facilities: 1 community-based, 3 other outpatient, 22 child/adolescent, 5 general hospital units, 1 mental hospital^58^ • Per 100k: General hospital 0.34 beds/1.22 admissions; Mental hospital 0.68 beds/1.49 admissions^58^ • Extremely low utilization per 100k: Community 0.23, Hospital-attached 57.90^58^ • 131 total admissions; 74.8% stay <1 year^58^ • Geographic barriers: 20 of 22 provinces require air travel (expensive, impossible for agitated patients)^62^  • Programmes: Suicide prevention, Papua New Guinea Hour of Hope TV (Anti-stigma), Susu Mamas (Early Child Development, NGO), Invest in Adolescence (School-based), Healthy Mind in Workplace, Disaster Response Plan ^58^ | • Majority pay nothing (fully insured) ^58^ • Drugs may be out of stock; patients purchase privately ^62^ • Limited-resource families: drugs only available payday, few days' supply; intermittent dosing increases relapse risk^62^ | • Mental health not systematically collected ^61^; data compiled only for general statistics last 2 years ^58^ • Dedicated authority conducts inspections yearly^58^; no formal Bougainville assessment^59^ • Major concerns: large-scale underreporting; urban-rural data collection disparities underrepresent rural majority^61^ |
| Pitcairn Islands | • Pacific Health Ministers Meeting (PHMM) commitment to multisectoral NCD plans and integrating mental health into climate adaptation plans^63^ • Pacific Islands Mental Health Network (PIMHNet) established in 2007; mental health declared a “top health priority” at 9th Pacific Health Ministers Meeting (2011)^63^ | • No information | • Human resource shortages consistently identified as a major systemic weakness^63^ • Significant capacity building and workforce development required to meet even minimum service delivery standards^63^ | • Many countries still rely on centralised psychiatric hospitals; others provide care via general hospitals in capital areas^63^ • Limited progress in decentralization; lack of community-based alternatives remains a major gap^63^ |  | • Lack of baseline mental health data is a major obstacle^63^ • Large gaps in epidemiology and comorbidity data; difficult to monitor trends or plan services^63^ • Absence of comprehensive data systems hinders evidence-based planning and evaluation^63^ • WHO/START initiative provides early progress toward improving suicide and self-harm surveillance^63^ |
| Samoa | • Policy development supported through focus groups involving staff, consumers, and researchers^64^ • National climate adaptation and disaster frameworks increasingly integrate mental health as a climate-sensitive issue^8,65^ | • Samoa operates a publicly funded, tax-based health system similar to the British model, covering medical care and medicines^9^ • Absence of dedicated mental health budgets limits sustainability; reliance on external or volunteer support remains high^12^ | • National Mental Health Service staffed by three mental health nurses and one part-time psychiatrist^66^ • Trained pastoral workers, youth facilitators, and childcare workers support psychosocial programs, including post-disaster interventions^66,67^ • Regionally, workforce shortages mirror Samoa’s situation—few psychiatrists/psychologists due to limited training capacity and professional isolation ^3,9^ • mhGAP has trained non-specialists in Samoa and across 14 countries to manage priority conditions; Samoan mental health nurses also serve as trainers ^9,25^ • UNICEF–WHO partnership trains child protection and education workers in psychological first aid and psychosocial support^12^ | • Services concentrated in Apia through the National Mental Health Service and NGOs such as SVSG and Goshen Trust^64,68^ • Community-based programs include art therapy initiatives (Tiapapata Art Centre) for trauma survivors and individuals with schizophrenia^64^ • Post-disaster interventions (Just Therapy, Tree of Life) delivered in schools and churches using storytelling, movement, and prayer^66,67^ • Household visits (asiasiga) reached 300+ families, providing screening, prayer, and referral support^66^ • Primary care uses mhGAP-trained staff for mild-to-moderate conditions; complex cases referred to central services^9,25^ • WHO/START supports suicide surveillance and awareness campaigns; provincial stress management units treat moderate–severe cases^25,40^ | • Persistent shortages of essential psychiatric medicines across the Pacific^25^ | • Existing data sources: 2013 Demographic and Health Survey, Child Protection Information System (CPIS), and the Global School-based Student Health Survey (GSHS)^65^ • Over 90% of people with mental disorders across the Pacific receive no treatment, yet reliable prevalence estimates are unavailable due to weak data systems^8,63^ |
| Solomon Islands | • Mental health governance centralised under Ministry of Health and Medical Services, acting as funder, regulator, and provider; minimal private sector role^69^ • National vision emphasizes community ownership and shift toward decentralised, community-based services^70^  • WHO reports a stand-alone mental health policy since 2009 and stand-alone legislation since 1970^71^ • Mental Treatment Act 1978 governs care, custody, and management of persons with mental disorders^69^ • National Mental Health Policy drafted in 2009 but reportedly never officially approved; mental health absent in general health policy^72^ • Integrated Mental Health Service aligned with WHO Comprehensive Mental Health Action Plan (2013–2020)^70^ | • Per capita health spending: SB$600; 13.5% of government spending; health expenditure = 5.45% of GDP (2015)^69,70^ • Funded by government, donors, and out-of-pocket payments; donors contribute ~35%, OOP ~7.3%^69^ • Tax-based British-style model: government pays fees and medicines; all meds free at point of care^69^ • Mental health services and psychotropic medicines fully insured and free of charge^71^ | • Mental health workforce: 2.09 professionals per 100,000 (0.30 psychiatrists, 1.34 nurses, 0.45 other specialists; no psychologists or social workers)^71^ • Geographic imbalances and severe shortages hinder service delivery^70^ • Training: Solomon Islands National University (SINU) provides 18 days of mental health training within nursing program^69^ • Community Mental Health & Psychosocial Rehabilitation course (4 weeks) strengthens leadership and service development skills^70^ • mhGAP training improved provider knowledge and confidence.  • 2013 Mental Health Disaster Preparedness Workshop strengthened post-disaster response capacity^70^ | • National services include: 20-bed National Psychiatric Unit (Auki); 4-bed Acute Care Unit (Honiara); community mental health team; provincial mental health coordinators^70^ • National Referral Hospital: 4 for mental health; provides outpatient care, counselling, referrals, outreach^69^ • One mental hospital with 2.99 beds/100,000; 131 admissions (74.8% <1 year duration)^71^ • Provincial psychiatric coordinators deliver education, clinical care, and referrals across six provinces^69^ | • All medicines—including psychotropic drugs—provided free of charge in the public system^69,71^ • All medicines imported; no domestic manufacturing^69^ • Pharmaceutical regulation governed by outdated Pharmacy and Poisons Act, creating enforcement challenges^69^ • Limited number of private pharmacies^69^ • Drug availability emphasized under mhGAP implementation | • No formal health technology assessment; reliance on WHO tools^69^ • No formal patient complaint/public feedback system; NGOs provide informal commentary^69^ • Administrative data not integrated across ministries; limits child protection monitoring^72^ • Beyond Global School-based Student Health Survey (GSHS), little quantitative data on adolescent or child mental health; no data for out-of-school youth^72^ • Very limited research output: 5 publications (2.03% of national research output; 0.1% of regional MH research output)^71^ |
| Tonga | • Slow policy development historically; lack of political champion despite WHO support (2001)^73^ • Significant progress: stand-alone mental health policy/plan (2020) and stand-alone mental health law (2020)^74^ • Mental Health Act 1992 grants psychiatrists authority to release involuntary patients; written appeal process strengthened due process^73^ • Suicide prevention programmes: Lifeline Tonga, regional hotline, anti-stigma initiatives^75^ | • Government mental health expenditure = 1.5% of total health spending^75^ • Most people pay nothing at point of service for mental health care and psychotropics (full public coverage)^75^ | • Relatively strong workforce for the Pacific: 17.23 mental health workers per 100,000 (0.96 psychiatrists, 15.31 mental health nurses, 0.96 psychologists, 0 social workers)^75^ • Workforce includes traditional healers, spirit healers, NGO community workers, and cultural workers^73,76^ • mhGAP training provided to non-specialist providers (GPs, nurses)^25^ | • 3 community mental health outpatient facilities; 1 day-treatment/outpatient facility; 1 general hospital psychiatric unit; 1 child/adolescent inpatient facility^75^ • 3.83 community-based mental health facilities per 100,000 population^75^ • 17.23 general-hospital psychiatric beds per 100,000; high annual admission rates^75^ • Child/adolescent beds: 6.26 per 100,000^75^ • Services also delivered through villages and churches; strong cultural acceptability enhances engagement^76^ • Traditional healing, talanoa (dialogue-based approach) culturally central^76^ | • Public system provides full coverage; psychotropic medicines free at point of use^75^ • mhGAP implementation prioritizes ensuring medication availability at service sites^9^ | • Dedicated authority conducts regular inspections, manages complaints, and reports annually^75^  • Mental health-specific data compiled within the last two years for public sector^75^ • Research output extremely limited: 0 mental health publications (0% of national and regional mental health research contribution)^75^ |
| Tokelau | • Highly decentralised health governance: since 2004, all health staff are employed by the taupulega (village councils); all policies and programmes must be approved by each taupulega^77^ • Decentralization has constrained system strengthening and national policy development^77^ • Lack of key national mental health policies and national-level schemes remains a major challenge^77^ | • Operates within a tax-based public health system, similar to the British model, with government-funded services and medicines^9^ | • Each atoll has a small hospital staffed by a medical officer, nurses, midwives, and hospital aides^77^ • No specialist mental health professionals (no psychiatrists, psychologists, or trained nurses)^77^ • Mental health is not included in standard health-worker training^77^ • Remote access to mental health specialists in New Zealand is available for severe acute cases^77^ • Tokelau has adopted mhGAP, training doctors and nurses in basic mental health care^9^ | • Health services delivered through one hospital per atoll; generally accessible^77^ • Major constraints: no X-ray or laboratory facilities, limiting diagnostic capacity^77^ • No on-island specialist mental health services; service delivery depends heavily on remote consultation from New Zealand^77^ • Climate change risks (storm surges, cyclones, drought) further disrupt access to care and essential services^77^ • Geographic isolation creates significant barriers to timely and continuous mental health care, despite remote support mechanisms^77^ | • WHO mhGAP emphasizes ensuring medication availability at local facilities^9^ • Persistent bottlenecks include irregular medicine supply, limited equipment, and poor access to specialized treatments^77^ • Patients repatriated from overseas using newer psychiatric medicines may need to switch temporarily if local supplies run out^77^ | • No prevalence data on mental illness in Tokelau^77^ • Limited adolescent MH data from the 2014 Global School-based Student Health Survey (GSHS)^77^ • Aside from GSHS and a 2012 WHO report, little quantitative mental health data exists for children or adolescents^77^ |
| Tuvalu | • Tuvalu has a stand-alone mental health law since 2008 and participates in the Pacific Islands Mental Health Network (PIMHNet)^78^ • An independent authority exists to monitor human-rights compliance of mental health legislation, but it is not functioning effectively^78^ • Government operates a Post-Disaster Rapid Assessment Team for mental health and psychosocial preparedness^78^ • SOLAR (Skills for Life Adjustment and Resilience) implemented with government–NGO–church collaboration (TANGO + EKT), demonstrating effective multisectoral leadership^79^ | • Tuvalu uses a tax-based public health system; government covers medical fees and medicines^9^ • Care and treatment for major mental health conditions (psychosis, bipolar disorder, depression) are included in national health insurance/reimbursement schemes^78^ | • Extremely limited mental health workforce: 0 psychiatrists, 0 mental health nurses, 3 psychologists (25.74/100,000), 1 social worker (8.58/100,000), and 0 other mental health specialists^78^ • Tuvalu has implemented mhGAP and trained doctors and nurses in basic mental health care • The SOLAR programme demonstrates a task-sharing model, showing that community-based workers can be trained to deliver evidence-based psychosocial interventions^79^ | • Mental health service availability is limited: 0 hospital-attached outpatient mental health facilities; 1 psychiatric unit in a general hospital (17.16 beds/100,000; 42.90 admissions/100,000) ^78^ • Total of 5 admissions to mental health hospitals, and 171.60 outpatient visits/100,000 in the past year^78^ • Strong reliance on informal supports (family, community, church) as part of usual care^79^ • SOLAR provides a low-intensity, scalable intervention for post-disaster distress, representing a key innovation in service delivery^79^ | • WHO mhGAP emphasizes ensuring psychotropic drug availability in Tuvalu^9^ • Government covers medication costs under the tax-based system^9^ | • Mental health data compiled only within general health statistics over the last two years^78^ • Six published mental health research articles: 7.32% of national research output and 0.2% of regional mental health research output^78^ • Major data gaps include: incomplete maternal/child health tracking, no data from the private sector, and inconsistent reporting on non-users of public services^80^ |
| Vanuatu | • Vanuatu has had an official mental health policy and plan since 2011^3^, a stand-alone mental health policy since 2016 and stand-alone law since 2017, with the Mental Health Act still under Ministry of Health review^81,82^ • The Mental Health Policy & Strategic Plan 2016–2020 emphasizes collaboration with churches, youth groups, women’s groups, and traditional healers to strengthen referral networks and culturally grounded care^83^ • Government programmes include Within Mental Health and Psychosocial Support (MHPSS), mhGAP, school-based prevention, maternal mental health, work-related prevention, and psychosocial components integrated into disaster preparedness and risk reduction^81^ • Key organizations include the National Mental Health Unit, VFHA, Wan Smol Bag, and Ministry of Health^3,83,84^ • During COVID-19, mandatory quarantine and a national health operations command system operated under the Public Health Act^85^ | • Vanuatu operates a tax-funded health system where government covers fees and medication costs^9^ • Treatment for psychosis, bipolar disorder and depression is covered by national insurance/reimbursement schemes; most service users pay nothing at the point of care^81^ • Only 1.0% of the total health budget is spent on mental health^81^ • Policy implementation is constrained by financial limitations, and external funding, especially post-disaster, is often short-term and inconsistent^84^ | • Total mental health workforce is 4.34 per 100,000: 0.33 psychiatrists, 2.67 mental health nurses, 0.85 psychologists, no social workers or other specialists, and 0.69 child/adolescent psychiatrists per 100,000^81^ • Historically, the country had no trained mental health doctors or nurses, and no NGOs dedicated to mental health^83^ • Current services include: 4 mental health beds, 1 psychiatrist and 3 MH nurses at Port Vila, 1 bed and 1 nurse at Luganville, plus 5 provincial hospitals each staffed by a mental health nurse with broader duties^82,83^  • Vanuatu has trained doctors and nurses through mhGAP^9^ • Vanuatu Psychiatry Mentorship Programme supports the sole psychiatrist and trained nurses, some trained via Fiji’s postgraduate programmes^82^ • Post-volcanic displacement response trained 101 volunteers in Psychological First Aid and psychosocial support^83^ | • Facilities include 6 outpatient mental health facilities attached to hospitals, 6 community-based mental health outpatient facilities, 2 child/adolescent outpatient facilities, 2 psychiatric units in general hospitals, and 2 child/adolescent inpatient units; total 2.0 community mental health facilities per 100,000^81^ • Psychiatric bed capacity: 1.00 per 100,000; child/adolescent inpatient beds: 2.07 per 100,000^81^ • Outpatient visits: 189.74 per 100,000 annually^81^ • Services are centralised in Port Vila and, to a lesser extent, Luganville; outer islands have minimal to no mental health capacity^84^ • Post-cyclone response delivered psychological first aid to 8,466 people; volunteers supported by NGOs and MH professionals^22,83^ • During COVID-19 quarantine, mental health assessments and psychosocial support were provided^85^ • Child & adolescent psychiatry volunteer mentoring (visits, online supervision, conjoint clinical care) is positively evaluated^14^ | • WHO mhGAP emphasizes ensuring drug availability at service points^9^ • Major barrier: inequitable access; psychotropic medication availability is limited and often inconsistent, sometimes in short supply^84^ • Only publicly funded antidepressant: amitriptyline; only antipsychotics: chlorpromazine, haloperidol, fluphenazine decanoate^86^ • Newer medications (e.g., risperidone) may be privately obtainable but are not included in the public system^82^ • Infrastructure and supply chain limitations affect health hardware and drug availability^86^ | • Very limited community-level mental health data; no systematic recording of prevalence or burden^84^ • No quantitative mental health data for adolescents or children beyond • No quantitative mental health data for adolescents or children beyond GSHS^87^ • Available data sources include WHO 2019 Mortality/Global Health Estimates, IHME 2019 GBD, and UNICEF *State of the World's Children 2021*^87^ |

**References**

1. J. Mew E, Hunt L, L.M. Toelupe R, et al. O le tagata ma lona aiga, o le tagata ma lona fa'asinomaga (Every person belongs to a family and every family belongs to a person): Development of a parenting framework for adolescent mental wellbeing in American Samoa. *Children & Youth Services Review* 2024; 160: N.PAG-N.PAG.

2. Galvin JE, Germain DM, Moore CP, Jeanty JA, Tofaeono V, Wiese LK. The Dementia Literacy Assessment (DeLA): A novel measure of Alzheimer's disease and related disorders health literacy in diverse populations. *ALZHEIMERS & DEMENTIA-TRANSLATIONAL RESEARCH & CLINICAL INTERVENTIONS* 2025; 11(1).

3. Charlson FJ, Diminic S, Whiteford HA. The Rising Tide of Mental Disorders in the Pacific Region. *Asia & the Pacific Policy Studies* 2015; 2(2): 280-92.

4. Mew EJ, Blas V, Winschel J, et al. "There are still broken or fragmented systems": Qualitative assessment of needs to strengthen adolescent mental health services in American Samoa. *Int J Ment Health Nurs* 2024; 33(1): 85-92.

5. WHO. Human Health and Climate Change in Pacific Island Countries. 2015. <http://iris.wpro.who.int/bitstream/handle/10665.1/12399/9789290617303_eng.pdf>.

6. Monteith LL, Holliday R, Iglesias CA, Sherrill A, Brenner LA, Hoffmire CA. Suicide risk and prevention in Guam: Clinical and research considerations and a call to action (vol 83, 103546, 2023). *Asian journal of psychiatry* 2023; 85.

7. Mengesha NA, Sarnyai Z. The mental health impact of climate change on Pacific Islanders: A systematic review focused on sea level rise and extreme weather events. *Australasian Psychiatry* 2025.

8. UNICEF. Situation Analysis of Children in the Pacific Island Countries. 2017. <https://www.unicef.org/pacificislands/reports/situation-analysis-children-pacific-island-countries>

9. Setoya Y, Kestel D. WHO Mental Health Gap Action Programme implementation in the Small Island Development States: experience from the Pacific and English-speaking Caribbean countries. *BJPsych Int* 2018; 15(2): 27-30.

10. WHO. WHO profile on mental health in development (WHO proMIND): Cook Islands. 2015. <https://iris.who.int/handle/10665/190410>

11. WHO. WHO proMIND: profiles on mental health in development: Republic of the Marshall Islands. 2015. <https://iris.who.int/handle/10665/185038>

12. UNICEF. Impact of COVID-19 on poor mental health in children and young people ‘tip of the iceberg’ – UNICEF. 2021. <https://www.unicef.org/pacificislands/press-releases/impact-covid-19-poor-mental-health-children-and-young-people-tip-iceberg-unicef>

13. Blignault I, Kaur A. Integration of traditional and western treatment approaches in mental health care in Pacific Island Countries. *Australasian Psychiatry* 2020; 28(1): 11-5.

14. Kowalenko N, Chang O, Hagali M, et al. Child and Adolescent Psychiatry International Relations (CAPIR): building bridges for psychiatry workforce capacity with Pacific Island nations. *Australasian Psychiatry* 2020; 28(1): 46-50.

15. Patterson T, Egan R, Gross J, Leov J, Hobbs L, La Rooy D. Mentoring: A Traditional Cook Island Approach to Support Men. *INTERNATIONAL JOURNAL OF OFFENDER THERAPY AND COMPARATIVE CRIMINOLOGY* 2023.

16. Ghiasuddin A. Child and adolescent psychiatric consultation in Micronesia. *Journal of Rural Mental Health* 2017; 41(4): 306-9.

17. Fung P, Montague R. A qualitative evaluation of leadership development workshops for mental health workers from four Pacific Island Countries. *Australasian Psychiatry* 2015; 23(3): 218-21.

18. WHO. Mental Health Atlas Micronesia (Federated States of ) 2020. 2021. <https://www.who.int/publications/m/item/mental-health-atlas-fsm-2020-country-profile>.

19. UNICEF. Situation Analysis of Children in the Federated States of Micronesia. 2017. <https://www.unicef.org/pacificislands/reports/situation-analysis-children-federated-states-micronesia>

20. Engelhard C, Haack S, Alik T. Implementation of the Mental Health Gap Action Programme (mhGAP) in Kosrae State in the Federated States of Micronesia. *Asia-Pacific journal of public health* 2021; 33(6-7): 784-5.

21. Nishio A, Shibuya F, de los Reyes CS, et al. Content analysis of health-related subjects in the K12 school curricula of Japan, Indonesia, Philippines, Guam, Micronesia, Marshall Islands, Palau, and Fiji. *TROPICAL MEDICINE AND HEALTH* 2023; 51(1).

22. Leal Filho W, Krishnapillai M, Minhas A, et al. Climate change, extreme events and mental health in the Pacific region. *International Journal of Climate Change Strategies and Management* 2022; 15(1): 20-40.

23. Charlson F, Chang O, Kubuabola I, et al. Implementation of the mental health Gap Action Programme (mhGAP) within the Fijian Healthcare System: a mixed-methods evaluation. *International journal of mental health systems* 2019; 13.

24. UNICEF. Situation Analysis of Children in Fiji. 2017. <https://www.unicef.org/pacificislands/reports/situation-analysis-children-fiji>

25. WHO Western Pacific. Regional framework for the future of mental health in the Western Pacific 2023-2030. 2023. <https://www.who.int/publications/i/item/9789290620075> (accessed 28 February 2025 2025).

26. Saxton K, Allan J, Gill N. Mental health as a human right: challenges and opportunities in Fiji. 2019.

27. WHO. Mental Health Atlas Fiji 2020. 2021. <https://www.who.int/publications/m/item/mental-health-atlas-fji-2020-country-profile>.

28. Fenner P, Ryan B, Nabukavou T, Chang O, Chetty S, Qaloewai S. “I function when I’m painting”— Consumers, carers and staff experiences of an art and mental health recovery project in Fiji. *The Arts in Psychotherapy* 2022; 77: 1-7.

29. Hoare F, Murphy N, O'Donoghue A, Allen S, Duffy RM. Gender-based provisions in mental health legislation: a review of English language jurisdictions. *Irish Journal of Psychological Medicine* 2025; 42(1): 50-6.

30. Chang OA, Allen MM, Pandit B. Capacity building for Pacific Island countries: the challenges and benefits of developing a postgraduate clinical training programme. *Australasian Psychiatry* 2015; 23(6): 32-4.

31. Robertson P, Paul A, Allen M. CAMH in Primary Care Fiji: developing child and adolescent mental health in primary care. *Australasian Psychiatry* 2020; 28(1): 37-41.

32. Iyengar MS, Chang O, Florez-Arango JF, Taria M, Patel VL. Development and usability of a mobile tool for identification of depression and suicide risk in Fiji. *TECHNOLOGY AND HEALTH CARE* 2021; 29(1): 143-53.

33. Johnston K, Qaloewai S, Rasavuka T, Preston R, Strivens E, Larkins S. 'Letting it be': a grounded theory about dementia care in Fiji. *Aging & mental health* 2024; 28(10): 1390-400.

34. Ryan B, Fenner P, Chang O, Qaloewai S, Nabukavou T, Chetty S. Art-making in mental health—A Fijian pilot study. *Australasian Psychiatry* 2021; 29(2): 204-6.

35. Sivakumaran H, George K, Naker G, Nadanachandran K. Experience from mental health clinics held during medical service camps in Fiji. *Australasian Psychiatry* 2015; 23(6): 667-9.

36. Nair M, Meirmanov S. Assessing the role of sustainability competencies in enhancing psychological first aid effectiveness for disaster responders in Fiji. *Frontiers in public health* 2024; 12.

37. O'Connor M, Rawstorne P, Iniakwala D, Razee H. Fijian adolescent emotional well-being and sexual and reproductive health-seeking behaviours. *Sexuality Research & Social Policy: A Journal of the NSRC* 2019; 16(3): 373-84.

38. Chang O, Patel VL, Iyengar S, May W. Impact of a mobile-based (mHealth) tool to support community health nurses in early identification of depression and suicide risk in Pacific Island Countries. *Australasian Psychiatry* 2021; 29(2): 200-3.

39. Chang O, Ryan B, Liebetrau E, Robertson P. Piloting online training in the Pacific-Ophelia project for child and adolescent mental health. *ASIA-PACIFIC PSYCHIATRY* 2022; 14(2).

40. Mathieu S, de Leo D, Koo YW, Leske S, Goodfellow B, Kolves K. Suicide and suicide attempts in the Pacific Islands: A Systematic Literature Review. *The Lancet regional health Western Pacific* 2021; 17: 100283.

41. Fenni A. État des lieux des suicides et tentatives de suicide en Polynésie Française. Exemple de l’enquête “Santé Mentale en Population Générale: Images et Réalités”, réalisée à Tahiti et Moorea en 2015. 2016. <https://policycommons.net/artifacts/15772350/state-of-knowledge-on-suicide-and-suicide-attempt-in-french-polynesia/16663150/>.

42. Zumbiehl L. Psychoéducation familiale : connaissances et attentes des aidants familiaux des patients souffrant de schizophrénie en Polynésie française. 2020. <https://policycommons.net/artifacts/15479186/family-psychoeducation/16372542/>

43. Zahlawi T, Roome AB, Chan CW, et al. Psychosocial support during displacement due to a natural disaster: relationships with distress in a lower-middle income country. *International health* 2019; 11(6): 472-9.

44. Gouniai JM, Smith KD, Leonte KG. Many common presentations of obsessive-compulsive disorder unrecognized by medical providers in a Pacific Island community. *JOURNAL OF MENTAL HEALTH TRAINING EDUCATION AND PRACTICE* 2022; 17(5): 419-28.

45. De Luna MJF, Kawabata Y. The role of enculturation on the help-seeking attitudes among Filipino Americans in Guam. *International Perspectives in Psychology: Research, Practice, Consultation* 2020; 9(2): 84-95.

46. Gouniai JM, Smith KD, Leonte KG. Do clergy recognize and respond appropriately to the many themes in obsessive-compulsive disorder?: Data from a Pacific Island community. *MENTAL HEALTH RELIGION & CULTURE* 2022; 25(1): 33-46.

47. UNICEF. Situation Analysis of Children in Kiribati. 2017. <https://www.unicef.org/pacificislands/reports/situation-analysis-children-kiribati>

48. Ministry of Health and Medical Services of Kiribati. 2017 Kiribati Annual Health Bulletin. 2017. <https://www.spc.int/DigitalLibrary/Doc/SDD/HEALTH/KI/2017_Kiribati_Annual_Health_Bulletin.pdf>

49. Ryan B, Viane M, Timmins F, Smith A, Anstey C. Bridging the ocean: Kiribati Australia alliance in mental health. *Australasian Psychiatry* 2017; 25(5): 474-7.

50. UNICEF. Situation Analysis of Children in the Marshall Islands. 2017. <https://www.unicef.org/pacificislands/reports/situation-analysis-children-marshall-islands>

51. WHO. Mental Health Atlas Marshall Islands 2020. 2021. <https://www.who.int/publications/m/item/mental-health-atlas-mhl-2020-country-profile>.

52. UNICEF. Situation Analysis of Children in Nauru. 2017. <https://www.unicef.org/pacificislands/reports/situation-analysis-children-nauru>

53. Newman L, O'Connor B, Reynolds V, Newhouse G. Pervasive refusal syndrome in child asylum seekers on Nauru. *Australasian Psychiatry* 2020; 28(5): 585-8.

54. UNICEF. Situation Analysis of Children in Niue. 2017. <https://www.unicef.org/pacificislands/reports/situation-analysis-children-niue>

55. WHO. Mental Health Atlas Niue 2020. 2021. <https://www.who.int/publications/m/item/mental-health-atlas-niu-2020-country-profile>.

56. UNICEF. Situation Analysis of Children in Palau. 2017. <https://www.unicef.org/pacificislands/reports/situation-analysis-children-palau>

57. WHO. Mental Health Atlas Palau 2020. 2021. <https://www.who.int/publications/m/item/mental-health-atlas-plw-2020-country-profile>.

58. WHO. Mental Health Atlas Papua New Guinea 2020. 2021. <https://www.who.int/publications/m/item/mental-health-atlas-png-2020-country-profile>.

59. Tierney D, Bolton P, Matanu B, Garasu L, Barnabas E, Silove D. The mental health and psychosocial impact of the Bougainville Crisis: A synthesis of available information. *International journal of mental health systems* 2016; 10.

60. Kowalenko N, Hagali M, Hoadley B. Building capacity for child and adolescent mental health and psychiatry in Papua New Guinea. *Australasian Psychiatry* 2020; 28(1): 51-4.

61. WHO. Independent State of Papua New Guinea Health System Review. 2019. <https://iris.who.int/handle/10665/280088>

62. Muga F. Rich Country, Poor People: the challenges of providing psychiatric services in the public and the private sectors in Papua New Guinea. *Australasian Psychiatry* 2015; 23(6): 29-31.

63. Ali S, Williams O, Chang O, Shidhaye R, Hunter E, Charlson F. Mental health in the Pacific: Urgency and opportunity. *Asia Pacific Viewpoint* 2020; 61(3): 537-50.

64. Fenner P, Ryan B, Latai L, Percival S. Art making and the promotion of wellbeing in Samoa—Participants’ lived experience of a recovery oriented intervention. *Arts & Health: An International Journal for Research, Policy and Practice* 2018; 10(2): 124-37.

65. UNICEF. Situation Analysis of Children in Samoa. 2017. <https://www.unicef.org/pacificislands/reports/situation-analysis-children-samoa>.

66. Tamasese TK, Parsons TL, Waldegrave C, Sawrey R, Bush A. Asiasiga: a Samoan intervention to address the immediate mental health needs of Samoan communities after a tsunami. *Australasian Psychiatry* 2020; 28(1): 31-3.

67. Tamasese TK, Bush A, Parsons TL, Sawrey R, Waldegrave C. Asiasiga i A'oga ma Nu'u: a child and adolescent post-tsunami intervention based on Indigenous Samoan values. *Australasian Psychiatry* 2020; 28(1): 34-6.

68. Ryan B, Goding M, Fenner P, et al. Art and mental health in Samoa. *Australasian Psychiatry* 2015; 23(6): 55-8.

69. Hodge N. Solomon Islands health system review. 2015. <https://iris.who.int/bitstream/10665/208212/1/9789290616931_eng.pdf>

70. Ryan B, Orotaloa P, Araitewa S, et al. Mental health in the Solomon Islands: developing reforms and partnerships. *Australasian Psychiatry* 2015; 23(6): 662-6.

71. WHO. Mental Health Atlas Solomon Islands 2020. 2021. <https://www.who.int/publications/m/item/mental-health-atlas-2020-country-profile--solomon-islands>.

72. UNICEF. Situation Analysis of Children in the Solomon Islands. 2017. <https://www.unicef.org/pacificislands/reports/situation-analysis-children-solomon-islands>

73. Poltorak M. Anthropology, brokerage, and collaboration in the development of a Tongan public psychiatry: Local lessons for global mental health. *Transcultural psychiatry* 2016; 53(6): 743-65.

74. UNICEF. Situation Analysis of Children in Tonga. 2017. <https://www.unicef.org/pacificislands/reports/situation-analysis-children-tonga>

75. WHO. Mental Health Atlas Tonga 2020. 2021. <https://www.who.int/publications/m/item/mental-health-atlas-ton-2020-country-profile>.

76. Vaka S, Hamer HP, Mesui-Henry A. The effectiveness of uloa as a model supporting Tongan people experiencing mental distress. *International Journal of Mental Health Nursing* 2022; 31(6): 1438-45.

77. UNICEF. Situation Analysis of Children in Tokelau. 2017. <https://www.unicef.org/pacificislands/reports/situation-analysis-children-tokelau>.

78. WHO. Mental Health Atlas Tuvalu 2020. 2021. <https://www.who.int/publications/m/item/mental-health-atlas-tuv-2020-country-profile>.

79. Gibson K, Little J, Cowlishaw S, Ipitoa Toromon T, Forbes D, O'Donnell M. Piloting a scalable, post-trauma psychosocial intervention in Tuvalu: The Skills for Life Adjustment and Resilience (SOLAR) program. *European Journal of Psychotraumatology* 2021; 12(1).

80. UNICEF. Situation Analysis of Children in Tuvalu. 2017. <https://www.unicef.org/pacificislands/reports/situation-analysis-children-tuvalu>

81. WHO. Mental Health Atlas Vanuatu 2020. 2021. <https://www.who.int/publications/m/item/mental-health-atlas-vut-2020-country-profile>.

82. Obed J, Bush A, Stathis S, Hunter E. The Vanuatu Psychiatry Mentorship Programme: supporting the development of a fledgling mental health service in the Pacific. *Australasian Psychiatry* 2020; 28(1): 24-6.

83. Nzayisenga E, Chan CW, Roome AB, et al. Patterns of distress and psychosocial support 2 years post-displacement following a natural disaster in a lower middle income country. *Frontiers in public health* 2022; 10.

84. Dawes N, Franklin RC, McIver L, Obed J. General and post-disaster mental health servicing in Vanuatu: A qualitative analysis. *INTERNATIONAL JOURNAL OF DISASTER RISK REDUCTION* 2019; 40.

85. Tapo PS, Knox TB, Van Gemert-Doyle C, et al. Lessons from COVID-19-free Vanuatu: intensive health operations for Phase 1 of repatriation and quarantine, May-July 2020. *Western Pacific Surveillance and Response* 2021; 12(1).

86. Obed J, Bush A, Stathis S, Hunter E. Vanuatu Psychiatry Mentorship Programme: a case illustrating cultural and clinical considerations. *Australasian Psychiatry* 2020; 28(1): 58-60.

87. UNICEF. Situation Analysis of Children in Vanuatu. 2017. <https://www.unicef.org/pacificislands/reports/situation-analysis-children-vanuatu>

**Supplementary Table 7** Mental health workforce interventions by Pacific Island countries and territories (PICTs)

| **PICTs** | **Interventions** |
| --- | --- |
| American Samoa | No information available |
| Cook Islands | RANZCP capacity-building; mhGAP training; proMIND training; Postgraduate Diploma in Mental Health programme; UNICEF-supported capacity building |
| Federated States of Micronesia | mhGAP training; proMIND training; Postgraduate Diploma in Mental Health programme; UNICEF-supported capacity building |
| Fiji | mhGAP training; proMIND training; Postgraduate Diploma in Mental Health programme; Master of Medicine in Psychiatry; Leadership in mental health training; Child and adolescent mental health training; St Giles Hospital in-service and arts-based programmes; UNICEF-supported capacity building; disaster response and preparedness training |
| French Polynesia | No information available |
| Guam | No information available |
| Kiribati | mhGAP training; proMIND training; UNICEF-supported capacity building; Leadership in mental health training; OPHELIA child and adolescent mental health training |
| Marshall Islands | mhGAP training; proMIND training; UNICEF-supported capacity building |
| Nauru | mhGAP training; proMIND training; UNICEF-supported capacity building |
| New Caledonia | No information available |
| Niue | proMIND training; UNICEF-supported capacity building |
| Northern Mariana Islands | mhGAP training; proMIND training |
| Palau | mhGAP training; proMIND training; UNICEF-supported capacity building |
| Papua New Guinea | WHO proMIND training; Master of Medicine in Psychiatry |
| Pitcairn Islands | No information available |
| Samoa | mhGAP training; proMIND training; UNICEF-supported capacity building |
| Solomon Islands | Solomon Islands National University mental health training; Community mental health and psychosocial rehabilitation training; mhGAP training; proMIND training; Mental health disaster preparedness workshops; UNICEF-supported capacity building |
| Tokelau | mhGAP training; proMIND training; UNICEF-supported capacity building |
| Tonga | mhGAP training; proMIND training; UNICEF-supported capacity building |
| Tuvalu | mhGAP training; proMIND training; UNICEF-supported capacity building; SOLAR psychosocial programme |
| Vanuatu | mhGAP training; proMIND training; UNICEF-supported capacity building |
| Wallis & Futuna | proMIND training |

mhGAP: Initiated mental health Gap Action Programme; RANZCP: Royal Australian and New Zealand College of Psychiatrists; SOLAR, Skills for Life Adjustment and Resilience; UNICEF: United Nations Children's Fund programme.

**Supplementary Table 8** Summary of mental health system performance across Pacific Island countries and territories (PICTs)

| **PICT** | **Overall system summary** |
| --- | --- |
| American Samoa | Centralised governance within the US system; small workforce, limited data, emerging community initiatives; overall low capacity with reliance on external support. |
| Cook Islands | Stand-alone mental health policy and legislation; workforce shortages; mhGAP implemented; services integrated into primary care; moderate capacity with progress in community-based care. |
| Federated States of Micronesia | Governance influenced by traditional structures; severe workforce shortages; limited facilities; partial mhGAP implementation; system largely underdeveloped. |
| Fiji | Emerging capacity; decentralised governance; mhGAP-trained workforce; community-based programmes; relatively strong policy framework; persistent rural and specialist service gaps. |
| French Polynesia | Centralised governance; active NGO and community partnerships; limited publicly available workforce and service data; moderately developed but without comprehensive regional coverage. |
| Wallis & Futuna | Extremely limited publicly available information; very small specialist workforce; overall system largely undocumented and likely minimal in capacity. |
| Guam | US territory context; mhGAP framework applied; workforce shortages persist; culturally hybrid service model; moderate capacity with reliance on federal resources. |
| Kiribati | Centralised system with outdated legislation; very few specialists; mhGAP initiated; underdeveloped service and information systems. |
| Marshall Islands | Centralised governance; limited local workforce; dependence on external funding; mhGAP implemented; low overall system capacity. |
| Nauru | Very small, centralised system; highly reliant on visiting specialists; minimal workforce; mhGAP initiated; extremely limited system capacity. |
| New Caledonia | Overall system largely undocumented. |
| Niue | Small population context; publicly funded services; mhGAP implemented; very limited specialist workforce; system constrained but functionally maintained due to scale. |
| Northern Mariana Islands | Limited publicly available information; engagement in WHO programmes (mhGAP/proMIND); overall system capacity appears low. |
| Palau | Centralised governance; some policy development and mhGAP-trained workforce; geographic dispersion constrains access; moderate overall performance. |
| Papua New Guinea | Large and geographically dispersed population; policy and legislation exist but implementation fragmented; severe workforce shortages; limited service coverage outside urban centres; system underdeveloped. |
| Pitcairn Islands | Extremely limited data; minimal health infrastructure and workforce; system capacity very low. |
| Samoa | Developing policy framework; mhGAP-trained workforce; active community and post-disaster initiatives; moderate capacity with ongoing workforce and medication constraints. |
| Solomon Islands | Centralised governance; limited workforce and service access; community-based services emerging; system underdeveloped. |
| Tonga | Relatively strong policy framework; culturally responsive services; mhGAP implemented; workforce and information system constraints remain; moderate capacity. |
| Tokelau | Highly decentralised governance; very small workforce and infrastructure; system capacity very low, reliant on local administration. |
| Tuvalu | Stand-alone legislation and active disaster-informed initiatives (e.g., SOLAR); extremely limited specialist workforce; single inpatient unit; strong community supports; moderate–emerging system constrained by capacity. |
| Vanuatu | Stand-alone policy and legislation; disaster-integrated programming; services centralised with limited rural access; small specialist workforce; restricted medicine availability; moderate-emerging system. |

mhGAP, WHO Mental Health Gap Action Programme; WHO, World Health Organization; NGO, non-governmental organisation; proMIND, Pacific Regional Mental Health Network Programme; US, United States; SOLAR, Skills for Life Adjustment and Resilience.
