# Supplementary material for: Mental health systems in Pacific island countries and territories: a scoping review of current evidence
Source: Lancet Reg Health West Pac. 2026 Apr 16;69:101856. doi: 10.1016/j.lanwpc.2026.101856 (PMC13101603; doi:10.1016/j.lanwpc.2026.101856)
Supplement: Study Protocol [file mmc2.docx]

**Supplementary File 1** Strengthening mental health systems in Pacific island countries and territories: A scoping review protocol

# INTRODUCTION

Mental health is a critical component of overall well-being, yet it remains a neglected area in many Pacific Island Countries and Territories (PICTs) [1]. The region is characterised by diverse cultures, languages, and socioeconomic contexts, which contribute to complex and varied mental health needs [2, 3]. Climate change further intensifies these challenges, as rising sea levels, extreme weather events, and other environmental disruptions displace communities, disrupt livelihoods, and threaten cultural continuity [4]. Such stressors increase psychological distress and place additional pressure on already limited mental health systems. While multiple, interconnected barriers to care exist, comprehensive information on system capacity, resources, and service delivery remains scarce.

Although efforts have been made to strengthen mental health systems across PICTs, progress has been uneven, and many countries remain underprepared to respond to emerging challenges, particularly during crises or emergencies. Limited data and research further constrain the development of effective policies and context-specific programmes, and formal health systems often operate alongside traditional leadership structures, extended family networks, and faith-based institutions, which play central roles in shaping community responses to mental health needs.

This scoping review synthesises available evidence on mental health systems across 22 PICTs, including American Samoa, Cook Islands, Federated States of Micronesia, Fiji, French Polynesia, Guam, Kiribati, Marshall Islands, Nauru, Niue, Northern Mariana Islands, Palau, Samoa, Solomon Islands, Tokelau, Tonga, Tuvalu, Vanuatu, Pitcairn Islands, Wallis and Futuna, New Caledonia, and Papua New Guinea. The review applies the World Health Organization (WHO) Health Systems Building Blocks framework to examine:

1. Leadership and governance – policies, strategic planning, regulation, and coordination of mental health services.
2. Service delivery – availability, quality, and accessibility of mental health care.
3. Health workforce – distribution, training, and competency of mental health personnel.
4. Financing – funding arrangements, resource allocation, and sustainability of services.
5. Medines – access to essential medications and therapeutic interventions.
6. Health information systems – availability and use of data to guide planning, monitoring, and evaluation.

The influence of climate change and emergencies will be considered across all building blocks to highlight their pervasive effect on mental health systems.

# METHODS

This review will be conducted adhere to the Preferred Reporting Items for Meta-Analysis and Systematic Reviews Extension for Scoping Reviews (PRISMA-ScR) statement [5].

##

## Search strategy

The review will identify both peer-reviewed and grey literature addressing the mental health systems in PICTs. Given the linguistic diversity of the region, the search will focus on English- and French-language publications. French is an official language in New Caledonia, French Polynesia, Vanuatu, and Wallis and Futuna; for these countries, English search terms will be adapted into French to capture relevant literature.

### Peer-review databases

A systematic search will be conducted across multiple bibliographic databases, including PubMed, CINAHL, EMBASE, Web of Science, PsycINFO, PAIS Index, and the WHO Western Pacific Region Index Medicus (WPRO). These databases were selected to cover a wide spectrum of mental health and health system literature relevant to PICTs. The search will include studies published from 2015 to the present, reflecting the period following the launch of the Sustainable Development Goals (SDGs) and capturing contemporary policies, challenges, and innovations in mental health care within the region.

A structured search strategy combining keywords and Medical Subject Headings (MeSH terms) will be applied to titles and abstracts (see Table 1). To ensure comprehensiveness, reference lists of included articles will also be hand-searched for additional relevant studies. Searches will be conducted in both English and French, with French-language searches applied to databases covering French-speaking PICTs. Additional French-language databases may be consulted in collaboration with French-speaking researchers to maximise coverage. All retrieved records will be managed using Covidence software for screening.

**Table 1 Search string for peer-review databases**

| **Search** | **Query** |
| --- | --- |
| #1 | “mental health”[MeSH Terms] OR “mental disorders”[MeSH Terms] OR “psychological well-being”[MeSH Terms] OR “anxiety”[MeSH Terms] OR “depression”[MeSH Terms] OR “euphoria”[MeSH Terms] OR “stress, psychological”[MeSH Terms] OR “self-Injurious behavior”[MeSH Terms] OR “epilepsy”[MeSH Terms] OR mental disorder*[tiab] OR mental disease*[tiab] OR mental illness*[tiab] OR mental health[tiab] OR mental well*being[tiab] OR psychiatric disorder*[tiab] OR psychiatric disease*[tiab] OR psychiatric illness*[tiab] OR psychiatric health[tiab] OR psychiatric well-being[tiab] OR psychosis[tiab] OR psychological disorder*[tiab] OR psychological disease*[tiab] OR psychological illness*[tiab] OR psychological health[tiab] OR psychological well*being[tiab] OR psychotic disorder*[tiab] OR psychotic disease*[tiab] OR psychotic illness*[tiab] OR psychotic health[tiab] OR psychotic well*being[tiab] OR developmental disorder*[tiab] OR developmental disease*[tiab] OR developmental illness*[tiab] OR developmental disabilit*[tiab] OR emotional disorder*[tiab] OR emotional disease*[tiab] OR emotional illness*[tiab] OR emotional health[tiab] OR behavioral disorder*[tiab] OR behavioral disease*[tiab] OR behavioral illness*[tiab] OR behavioral health[tiab] OR behavioral disabilit*[tiab] OR anxiety[tiab] OR depressive disorder*[tiab] OR depression[tiab] OR schizophrenia[tiab] OR bipolar disorder*[tiab] OR self-injur*[tiab] OR self-harm[tiab] OR suicide[tiab] OR autism[tiab] OR asperger[tiab] OR attention-deficit*hyperactivity disorder[tiab] OR ADHD[tiab] OR epilepsy[tiab] OR dementia[tiab] |
| #2 | “pacific islands”[MeSH Terms] OR pacific island*[tiab] OR pacifik island*[tiab] OR south pacific[tiab] OR south pacifik[tiab] OR samoa[tiab] OR cook island*[tiab] OR micronesia[tiab] OR fiji[tiab] OR french polynesia[tiab] OR guam[tiab] OR i-kiribati[tiab] OR kiribati[tiab] OR marshall island*[tiab] OR nauru[tiab] OR niue[tiab] OR northern mariana island*[tiab] OR palau[tiab] OR solomon island*[tiab] OR tokelau[tiab] OR tonga[tiab] OR tuvalu[tiab] OR vanuatu[tiab] OR pitcairn island*[tiab] OR new caledonia[tiab] OR papua new guinea[tiab] OR melanesia[tiab] OR (wallis[tiab] AND futuna[tiab]) OR gilbertese[tiab] OR gilbert island*[tiab] OR makin[tiab] OR butaritari[tiab] OR marakei[tiab] OR abaiang[tiab] OR tarawa[tiab] OR maiana[tiab] OR abemama[tiab] OR kuria[tiab] OR aranuka[tiab] OR nonouti[tiab] OR tabiteuea[tiab] OR beru[tiab] OR nikunau[tiab] OR onotoa[tiab] OR tamana[tiab] OR arorae[tiab] OR marshallese*[tiab] OR marshall island*[tiab] OR papuan*[tiab] OR choiseul*[tiab] OR shortland island*[tiab] OR new georgia island*[tiab] OR russell island*[tiab] OR tulagi*[tiab] OR malaita[tiab] OR maramasike[tiab] OR ulawa[tiab] OR owaraha[tiab] OR makira[tiab] OR guadalcanal[tiab] OR tongan*[tiab] OR ellice island*[tiab] OR nanumanga[tiab] OR niutao[tiab] OR niulakita[tiab] OR vanuatu*[tiab] |
| #3 | #1 AND #2 |
| #4 | Restrict to 2015 onwards |

### Grey literature

Grey literature will be identified through three complementary approaches: (1) Google search, (2) grey literature databases, and (3) targeted websites.

**Google search**

We will establish a search string for the PICTs of interest and put that string into the Google search box and then run (Table 2). As French is the official language in French Polynesia, New Caledonia, Vanuatu, and Wallis & Futuna, we will also use French key words to search for these four PICT.

**Table 2 Search string for Google search**

| (mental OR psychological OR psychiatric OR psychosis OR suicide) (pacific island OR pacifik island OR samoa OR cook island OR micronesia OR fiji OR french polynesia OR guam OR kiribati OR marshall island OR nauru OR new caledonia OR niue OR northern mariana island OR palau OR papua new guinea OR pitcairn island OR solomon island OR tokelau OR tonga OR tuvalu OR vanuatu OR wallis OR futuna) after:2015-01-01 (filetype:pdf OR filetype:ppt OR filetype:pptx OR filetype:doc OR filetype:docx) |
| --- |

**Grey literature databases**

We will systematically search grey literature databases that index policy reports, government documents, and institutional publications. The following databases and repositories will be included in our search: Policy Commons, Dimensions, OECD iLibrary, World Bank Documents and Reports, World Bank Open Knowledge Repository, and WHO Institutional Repository for Information Sharing (IRIS). To optimize search precision, we will develop tailored search strings specific to each database, ensuring that relevant documents related to mental health in PICTs are identified (see Table 3).

**Table 3 Search string for grey literature databases**

| **Grey literature databases** | **Search string** |
| --- | --- |
| Policy Commons | summary:("maladie mentale" OR "trouble mental" OR "troubles mentaux" OR "trouble psychiatrique" OR "santé mentale" OR "suicide" OR "psychose" OR "psychologique" OR "psychiatrique") AND summary:("Polynésie française" OR "Vanuatu" OR "Wallis-et-Futuna" OR "Nouvelle-Calédonie") |
| Dimensions | ("maladie mentale" OR "trouble mental" OR "troubles mentaux" OR "trouble psychiatrique" OR "santé mentale" OR "suicide" OR "psychose" OR "psychologique" OR "psychiatrique") AND ("Polynésie française" OR "Vanuatu" OR "Wallis-et-Futuna" OR "Nouvelle-Calédonie") |
| OECD iLibrary | ("maladie mentale" OR "trouble mental" OR "troubles mentaux" OR "trouble psychiatrique" OR "santé mentale" OR "suicide" OR "psychose" OR "psychologique" OR "psychiatrique") AND ("Polynésie française" OR "Vanuatu" OR "Wallis-et-Futuna" OR "Nouvelle-Calédonie") |
| World Bank Documents and Reports | ("maladie mentale" OR "trouble mental" OR "troubles mentaux" OR "trouble psychiatrique" OR "santé mentale" OR "suicide" OR "psychose" OR "psychologique" OR "psychiatrique") AND ("Polynésie française" OR "Vanuatu" OR "Wallis-et-Futuna" OR "Nouvelle-Calédonie") |
| World Bank Open Knowledge Repository | ("maladie mentale" OR "trouble mental" OR "troubles mentaux" OR "trouble psychiatrique" OR "santé mentale" OR "suicide" OR "psychose" OR "psychologique" OR "psychiatrique") AND ("Polynésie française" OR "Vanuatu" OR "Wallis-et-Futuna" OR "Nouvelle-Calédonie") |
| WHO IRIS | (Regional Office for the Western Pacific) AND [2015 TO 2025] AND French AND (“santé mentale” [MeSH] OR “troubles mentaux” [MeSH]) AND ("Polynésie française" OR "Vanuatu" OR "Wallis-et-Futuna" OR "Nouvelle-Calédonie") |

**Targeted website sources**

We will systematically search targeted website sources. The following sources will be included in our search: United Nations International Children's Emergency Fund (UNICEF) Pacific Islands, WHO Mental Health Atlas, Pacific Community, Pacific Island Health Officers Association (PIHOA), and Asian Development Bank. To optimize search precision, we will develop tailored search strings specific to each database, ensuring that relevant documents related to mental health in PICTs are identified (Table 4).

**Table 4 Search string for targeted website sources**

| **Targeted website sources** | **Search string** |
| --- | --- |
| WHO Mental Health Atlas | Latest atlas reports after 2015-01-01 in PICTs (“American Samoa” OR “Cook Islands” OR “Federated States of Micronesia” OR “Fiji” OR “French Polynesia” OR “Guam” OR “Kiribati” OR “Marshall Islands” OR “Nauru” OR “Niue” OR “Northern Mariana Islands” OR “Palau” OR “Samoa” OR “Solomon Islands” OR “Tokelau” OR “Tonga” OR “Tuvalu” OR “Vanuatu” OR “Pitcairn Islands” OR “Wallis and Futuna” OR “New Caledonia” OR “Papua New Guinea”) |
| UNICEF Pacific Islands | Mental health after 2015-01-01 |
| Pacific Community | (“mental ill” OR “mental illness” OR “mental disorders” OR “mental disease” OR “mental health” OR “suicide” OR “psychosis” OR “psychological” OR “psychiatric”) |
| Pacific Island Health Officers Association (PIHOA) | (“mental ill” OR “mental illness” OR “mental disorders” OR “mental disease” OR “mental health” OR “suicide” OR “psychosis” OR “psychological” OR “psychiatric”) AND (2015-2025) |
| Asian Development Bank | (“mental ill” OR “mental illness” OR “mental disorders” OR “mental disease” OR “mental health” OR “suicide” OR “psychosis” OR “psychological” OR “psychiatric”) AND (2015-2025) |

Consistent with our peer-reviewed literature search strategy, we will restrict grey literature searches to English-language documents for most PICTs. However, for New Caledonia, French Polynesia, Vanuatu, and Wallis and Futuna, where French is the official language, we will also search for and include grey literature in French to ensure the inclusion of all relevant national and regional publications.

##

## Study selection

Titles, abstracts, and full texts of retrieved records will be independently screened by two reviewers, with discrepancies resolved through discussion. Eligible studies will include quantitative and qualitative research reporting on mental health systems within PICTs. Excluded items will comprise conference abstracts, study protocols, pre-prints, studies conducted outside PICTs, and studies not reporting on outcomes of interest (e.g., leadership and governance, service delivery, health workforce, financing, access to essential medicines, health information systems). All reviewers will participate in a training session on inclusion and exclusion criteria. This session will enhance consistency in understanding criteria and screening process across the team.

**Data extraction**

To support standardised data collection, the review team will conduct interactive meetings to collaboratively develop a data extraction form, ensuring that all relevant domains and indicators are captured consistently. Data extraction will be conducted independently by two reviewers using an Excel spreadsheet (version 2408), capturing publication details (author, year, location, study design), participant characteristics (sample size, demographics), and the six domains of WHO Health Systems Building Blocks framework. Discrepancies will be resolved by consensus.

## Data synthesis and analysis

Findings will be synthesised using a narrative qualitative approach, allowing for a rich and descriptive presentation of the evidence. Results will be summarised at the country level, providing a tailored perspective on the mental health systems of each PICT. This approach will facilitate the identification of contextual factors, challenges, and opportunities specific to each setting, enhancing the relevance and applicability of the findings for policy and practice.

References

[1] Ali S, Williams O, Chang O, Shidhaye R, Hunter E, Charlson F. Mental health in the Pacific: Urgency and opportunity. Asia Pacific Viewpoint. 2020;61(3):537-50.

[2] Prasad N. Growth and social development in the Pacific Island countries. International Journal of Social Economics. 2008;35(12):930-50.

[3] Feeny S, Iamsiraroj S, McGillivray M. Growth and Foreign Direct Investment in the Pacific Island countries. Economic Modelling. 2014;37:332-9.

[4] Weir T, Dovey L, Orcherton D. Social and cultural issues raised by climate change in Pacific Island countries: an overview. Regional Environmental Change. 2017;17(4):1017-28.

[5] Tricco AC, Lillie E, Zarin W, O'Brien KK, Colquhoun H, Levac D, et al. PRISMA Extension for Scoping Reviews (PRISMA-ScR): Checklist and Explanation. Ann Intern Med. 2018;169(7):467-73.
